# A facile three-component route to powerful 5-aryldeazaalloxazine photocatalysts

**DOI:** 10.3762/bjoc.20.161

**Published:** 2024-07-31

**Authors:** Ivana Weisheitelová, Radek Cibulka, Marek Sikorski, Tetiana Pavlovska

**Affiliations:** 1 Department of Organic Chemistry, University of Chemistry and Technology, Prague, Czech Republic; 2 Faculty of Chemistry, Adam Mickiewicz University, Poznań, Poland

**Keywords:** catalysis, deazaalloxazine, flavin, multicomponent approach, one-pot reaction

## Abstract

Functionalized 5-aryldeazaalloxazines have been successfully synthesised through a one-pot, three-component reaction involving *N,N*-dimethylbarbituric acid, an aromatic aldehyde and aniline. By utilizing readily available reagents, this approach opens up the opportunity for the efficient formation of a variety of 5-aryldeazaalloxazines bearing electron-donating or halogen groups. This practical method is characterised by atom economy and offers a direct route to the introduction of an aryl moiety into the C(5)-position of deazaalloxazines, thereby generating novel catalysts for photoredox catalysis without the need for subsequent purification. Thus, it significantly improves existing approaches.

## Introduction

**Figure 1:**
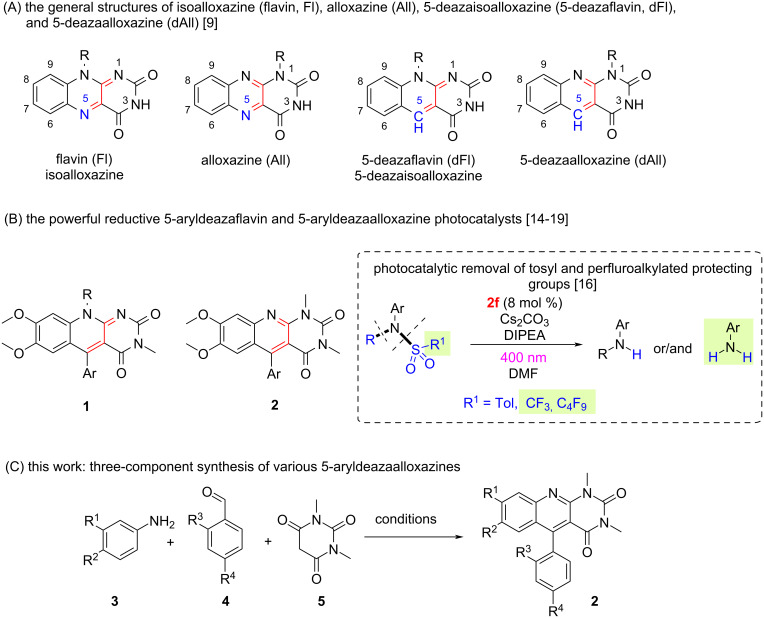
(A) The general structures of isoalloxazine (flavin, **Fl**), alloxazine (**All**), 5-deazaisoalloxazine (5-deazaflavin, **dFl**), and 5-deazaalloxazine (**dAll**). (B) The powerful reductive photocatalysts: 5-aryldeazaflavin (**1**) and 5-aryldeazaalloxazine (**2**). (C) This work, which describes an efficient three-component method for the synthesis of **2**.

Heterocyclic compounds containing pyrimidine and quinoline motifs in their structure, both of natural and synthetic origin, find a wide set of applications in medicinal chemistry, chemosensors, polymers and catalysis [1–8]. Among them, flavins (Fl) are essential redox-active natural compounds that act as enzyme cofactors in numerous biochemical processes [9]. Structurally related to flavins are isomeric alloxazines (All) and also 5-deazaflavins (dFl) and 5-deazaalloxazines (dAll), where the N(5) atom of the isoalloxazine/alloxazine core is replaced by a C–H moiety (Figure 1A). In particular, 5-deazaflavins have generated considerable interest from scientists to study flavin-catalysed reactions in enzymatic and artificial systems. Additionally, 5-deazaflavins have emerged as prospective antitumor agents [9–10]. Surprisingly, among the broad family of flavin derivatives, 5-deazaalloxazines have received less attention regarding their photophysical properties, despite their close similarity to the above-mentioned 5-deazaflavins [11–13]. Recently, it has been discovered that both 5-deazaflavins **1** and 5-deazaalloxazines **2**, which have an aryl substituent in position C(5), form stable radicals that act as powerful reductive photocatalysts with a reducing power comparable to that of lithium [E*(**1/1^•^**) = −3.3 V vs SCE, value for Ar = Ph] [14–18] (Figure 1B). 5-Aryldeazaalloxazines **2** have been found to be even more powerful reductants than **1** due to their more negative ground-state reduction potential by ca. 300 mV. Moreover, **2** exhibits higher photostability than **1**. Consequently, 5-aryldeazaalloxazine **2f** has been successfully applied as photoredox catalyst in the synthesis of secondary or primary anilines via light-dependent desulfonylation or desulfonylation/dealkylation procedures [19].

Thus, the design of novel and efficient routes for the synthesis of 5-aryldeazaalloxazines **2** has become a significant topic. Surprisingly, there has been limited information on 5-deazaalloxazines (dAll) synthesis [20–22]. Most known methods employ the cyclization of 6-(arylamino)uracils with one-carbon reagents such as triethyl orthoformate, dimethylformamide dimethylacetal, carbon disulfide, *N,N*-dimethyldichloromethyleniminium chloride and the Vilsmeier reagent, or condensations between *o*-aminobenzaldehydes and barbituric acid [20–21,23–27]. Neither of these methods allows for the introduction of an aryl substituent into C(5), which confers unique chemical and physical properties on 5-aryldeazaalloxazines **2**, as demonstrated in our previous studies with 5-aryldeazaflavins **1** [14–18].

Multicomponent reactions (MCRs) remain a powerful strategy in synthetic organic chemistry due to their widespread applications in drug discovery. By offering significant advantages over conventional, linear-type syntheses, MCRs have become helpful tools for more efficient preparation of chemical libraries with higher molecular diversity and complexity in fewer steps and less time [28–29].

Considering the limitations of existing methods, as well as the applications of 5-aryldeazaalloxazines **2**, and in the continuation of our work on the synthesis of powerful photoredox flavins [14–19], we developed an efficient, general, one-pot domino method for the synthesis of a series of novel 5-aryldeazaalloxazines **2** from readily available substrates via a three-component fashion (Figure 1C).

## Results and Discussion

Regarding the synthesis of 5-aryldeazaalloxazines **2** (5-arylpyrimido[4,5-*b*]quinoline-2,4(1*H*,3*H*)-diones), the data in the literature are quite limited, and the known methodology describes the dehydrogenation of initially formed 5,10-dihydro analogues (5-aryl-5,10-dihydropyrimido[4,5-*b*]quinoline-2,4(1*H*,3*H*)-dione) by refluxing with thionyl chloride [20,23]. However, the preparation of partially hydrogenated 5,10-dihydropyrimido[4,5-*b*]quinolinediones has been repeatedly reported by one-pot condensation of substituted anilines, aldehydes and barbituric acids, usually in protic solvents (alcohols, water), which has become a common method for the synthesis of these derivatives [3,22,25,30–33].

In our previous studies [14–19] we have shown that the 5-aryl and 7,8-substitutients of the pyrimido[4,5-*b*]quinoline core have a significant effect on the photocatalytic activity of photocatalysts by tuning their redox and photophysical properties. Thus, we successfully developed a one-pot, three-component synthetic method with those substituents in 5-aryldeazaflavins **1** on the deazaisoalloxazine core or on the phenyl ring by condensation of *N*-substituted anilines, aromatic aldehydes and *N*-methylbarbituric acid in AcOH/PPA (polyphosphoric acid). However, this method was not successful for the synthesis of 5-aryldeazaalloxazines **2**, and we never observed the formation of 5,10-dihydropyrimido[4,5-*b*]quinolinediones either. We noticed that the chemical nature of the solvent was highly important for the outcome of this reaction. Thus, we decided to significantly improve our approach by optimizing the reaction conditions of a three-component condensation of commercially available 3,4-dimethylaniline (**3a**, 1.0 mmol), benzaldehyde (**4a**, 1.0 mmol) and *N*,*N*-dimethylbarbituric acid (**5**, 1.0 mmol).

As illustrated in Table 1, DMSO was preferred as the optimal solvent, and 130 °C was chosen as the most suitable reaction temperature (Table 1, entry 1). Other solvents showed low yields, or the product was not isolated at all. However, we found that the yield of 5-phenyldeazaalloxazine **2a** was significantly enhanced when refluxing in DMF with a catalytic amount of AlCl_3_ compared to the reaction in clear DMF (Table 1, entries 7–9). Other Lewis acids were not as effective, except for a combination of DMSO/TMSOTf (Table 1, entry 4). The application of AcOH/PPA for the synthesis of 5-aryldeazaflavins did not improve the result of the reaction (Table 1, entry 10). We also found that the yield of **2a** may be slightly affected by the volume of solvent, with increasing amounts of impurities in larger volumes.

**Table 1:** Optimization of the reaction conditions for the synthesis of **2a**.

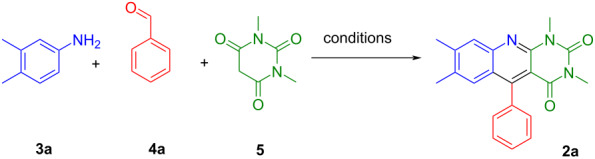						

Entry	Solvent	Time (h)	Volume (mL)	Additive	Conditions	Yield^a^ (%)

1	DMSO	15	**2**	–	CH	**57**
2	DMSO	15	**10**	–	CH	52
3	DMSO	15	2	**AlCl** **_3_**	CH	**55**
4	DMSO	15	2	**TMSOTf**	CH	**48**
5	DMSO	1	2	–	**MW**	32
6	DMSO	1	2	AlCl_3_	**MW**	39
7	DMF	15	2	–	CH	11
8	DMF	15	2	**AlCl** **_3_**	CH	**41**
9	DMF	1	2	AlCl_3_	**MW**	26
10	AcOH	15	2	PPA	CH	28
11	AcOH	15	2	H_2_SO_4_	CH	33
12	AcOH	1	2	H_2_SO_4_	MW	37^b^

^a^The isolated yields were calculated on the quantities of the starting materials **1–3**. ^b^The product was contaminated with resinificated impurities.

We also tested the response of the **2a** formation to MW irradiation and under conventional heating conditions (CH). Syntheses were performed in DMSO, DMSO/AlCl_3_, DMF/AlCl_3_ and AcOH/H_2_SO_4_ at 110 °C (Table 1, entries 5, 6, 9, and 12). Under these conditions, the reaction time was significantly reduced to 1 hour from the usual 15 hours, however, the yields stayed in the range of 30%, and the reaction mixtures contained a large number of impurities.

In summary, standard heating conditions exhibited distinct advantages over MW, and the reaction performance in DMSO was the most effective. Moreover, DMSO is considered an environmentally friendly solvent (for detailed information on reaction condition optimisation, see Supporting Information File 1).

**Scheme 1:**
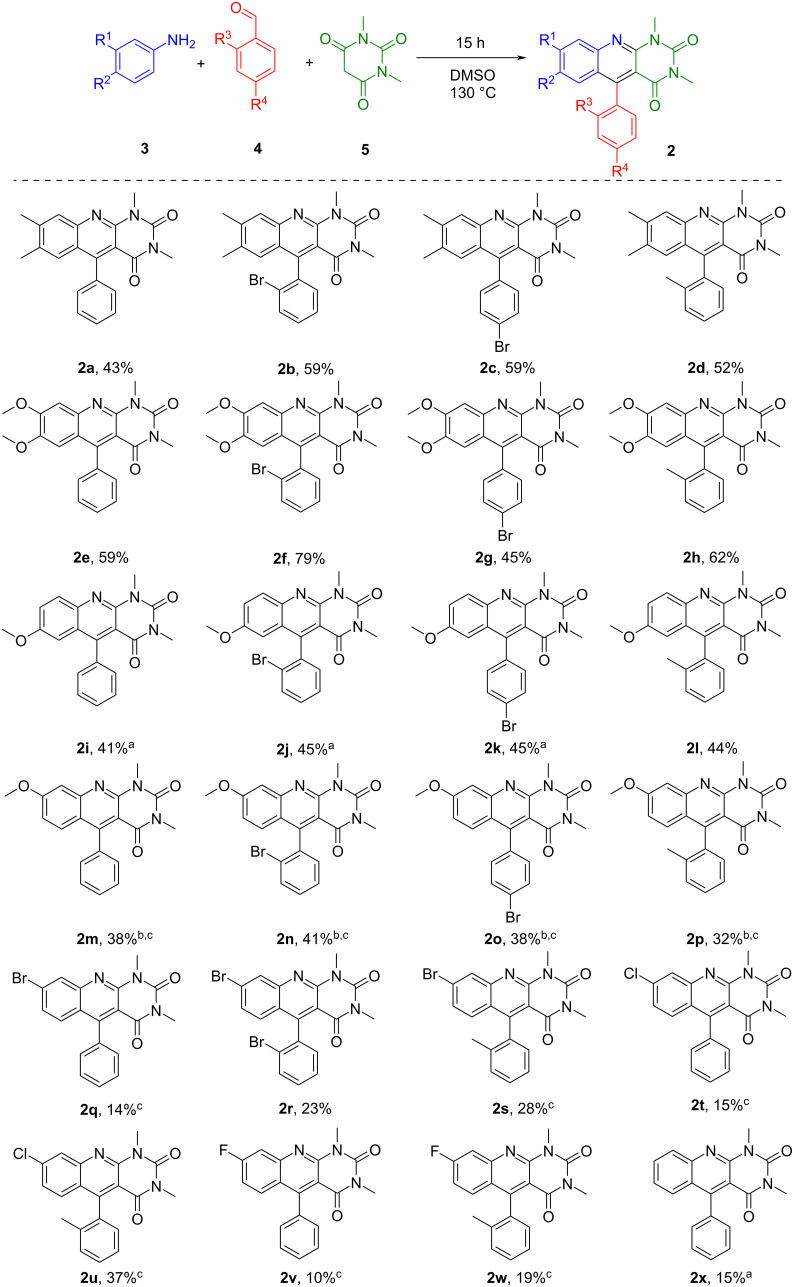
Three-component condensation of anilines, aldehydes and *N*,*N*-dimethylbarbituric acid. ^a^Reaction was done in DMF/AlCl_3_. ^b^Isolation requires precipitation with 2-propanol. ^c^2 days.

Under optimised reaction conditions (DMSO, 130 °C unless otherwise indicated, 15 h), a series of 5-aryldeazaalloxazines **2a**–**x** was synthesised in the 3.0 mmol scale with moderate to good yields (Scheme 1). Our protocol was successfully applied to various anilines and aromatic aldehydes with electron-donating groups or electron-withdrawing halogen atoms. However, the reaction yield was affected by the nature of the substituent on the aniline moiety. The results suggest that substrates bearing electron-donating groups on anilines have higher reactivity, thus giving higher yields than those bearing electron-withdrawing groups. The best reactivity was observed for 3,4-dimethylaniline (**3a**) and 3,4-dimethoxyaniline (**3b**) with the isolation of 5-aryldeazaalloxazines **2a**–**h,** with yields of 43–79%. Additionally, 5-aryldeazaalloxazines bearing methoxy substituents at positions 7 and 8 of the deazaalloxazine ring (see Figure 1 for numeration) showed the best reactivity as reductive photocatalysts. Such an outcome of MCR opens up the possibility of their production on a larger scale for commercial needs. However, the synthesis of the 7- and 8-methoxy derivatives, **2i**–**l** and **2m**–**p**, respectively, was accompanied by several complications, and the isolation of the products required precipitation with 2-propanol in several cases. The heating time was prolonged to two days for 8-methoxy derivatives **2m**–**p**, nevertheless giving moderate yields. For 7-methoxy derivatives **2i**–**k**, we changed the solvent to DMF/AlCl_3_, which increased the yields to 41–45%. However, the 7-methoxy derivative **2l** with *o*-methyl substituent in the aldehyde moiety precipitated in DMSO after two days of reaction time. Interestingly, we observed only traces of another possible regioisomer **2m’** with a methoxy group in position C(6) in the ^1^H NMR spectrum when using *m*-anisidine (**3c**) only with benzaldehyde (**4b**, for detailed information, see Supporting Information File 1). It should be mentioned that three-component condensation leading to the formation of 5-aryldeazaflavins **1** usually proceeds via Knoevenagel adduct formation as a first step, which may become a significant limitation of this reaction. However, we have never observed and isolated Knoevenagel adducts of aromatic aldehydes **4** and *N,N*-dimethylbarbituric acid (**5**) during the synthesis of 5-aryldeazaalloxazines **2a**–**x** in either of the described conditions.

The halogen groups on the anilines slightly decreased their reactivity, leading to the formation of products **2q**–**w** with lower yields. However, the 8-chlorine derivative **2u** with a *o*-tolyl aldehyde moiety was prepared with a yield of 37%. Aromatic aldehydes bearing bromine and methyl substituents were chosen to enhance the photophysical properties and photostability of the desired photocatalysts. It should be noted that our method encountered limitations when applying strongly deactivated anilines substituted with CF_3_ or acetyl groups, with no formation of 5-aryldeazaalloxazines in any case. Finally, the unsubstituted derivative **2x** was formed with a yield of only 15% using DMF/AlCl_3_.

The isolated products **2a**–**x** were characterized by ^1^H, ^13^C NMR and mass-spectral methods. The ^1^H NMR spectra of 5-aryldeazaalloxazines **2a**–**x**, along with protons of aryl substituents of aldehyde moiety and pyrimido[4,5-*b*]quinoline core, contained singlets with 3H intensity of the N(1)–CH_3_ and N(3)–CH_3_ groups of the barbituric acid fragment at 3.20–3.56 ppm. The ^13^C NMR spectra of 5-aryldeazaalloxazines **2a**–**x** were represented by groups of singlets at 27.9–57.2 and 100.0–160.5 ppm. The characteristic signals of the carbonyl atoms were seen as singlets at 155.6–160.5 ppm. Signals of the carbon atoms of the pyrimido[4,5-*b*]quinoline ring were located in the resonance region of the carbon atoms of the aryl substituents. Taken together, these data indicate the formation of the 5-arylpyrimido[4,5-*b*]quinoline-2,4(1*H*,3*H*)-dione cyclic system.

**Figure 2:**
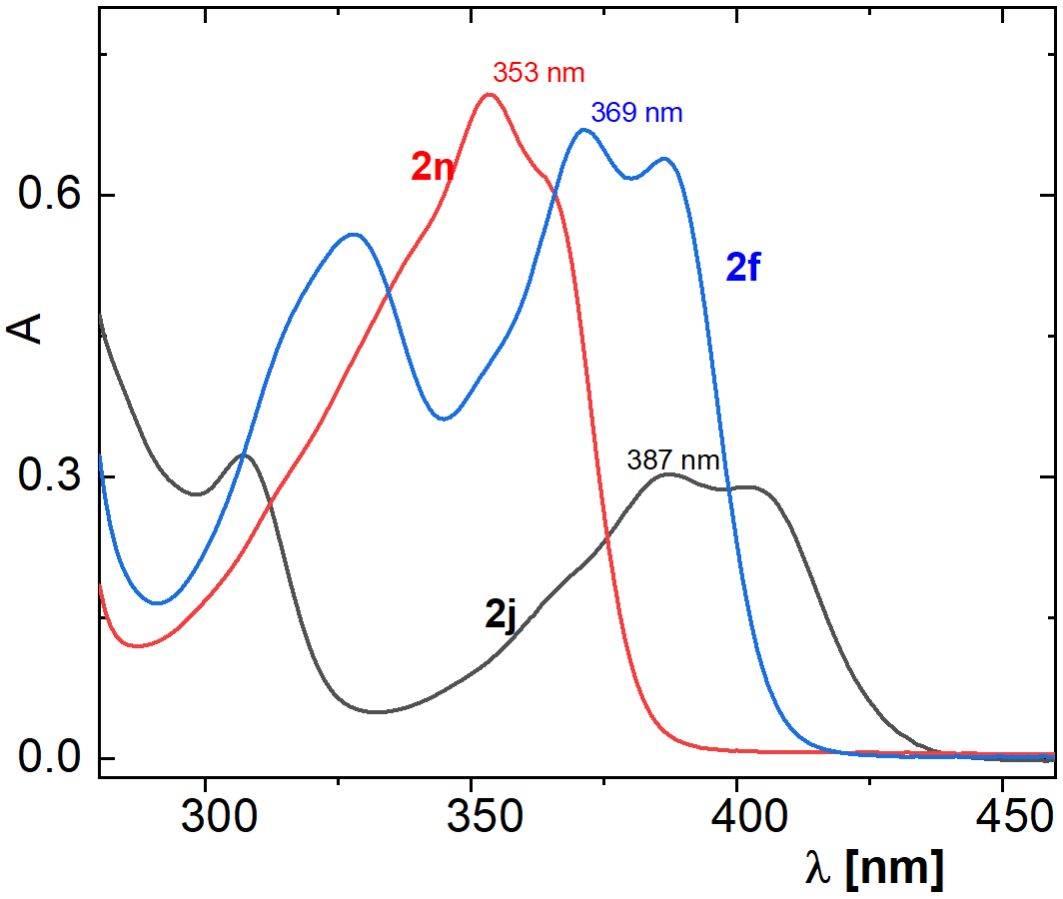
UV–vis absorption spectra of 5-arydeazaalloxazines **2f**, **2j** and **2n** in DMF (*l* = 1 cm, *c* = 2.50 × 10^−5^ mol·L^−1^).

The absorption spectra of the methoxy derivatives **2f**, **2j** and **2n** in DMF provide interesting information on the effect of the position of the methoxy group in the core of deazaalloxazine on the absorption maxima (Figure 2). The 8-methoxydeazaalloxazine **2n** exhibited a characteristic band at 353 nm (ε = 24.4 × 10^3^ M^−1.^cm^−1^), while it moved to 370 nm for 7,8-dimethoxydeazaalloxazine **2f** (ε = 26.2 × 10^3^ M^−1.^cm^−1^) and even to 387 nm (ε = 12.2 × 10^3^ M^−1.^cm^−1^) for 7-methoxydeazaalloxazine **2j**. This indicates that, besides the 7,8-dimethoxy derivatives usually studied in deazaflavin/alloxazine photoredox catalysis [9,14–19], 7-methoxyderivatives should also be considered due to their absorption closer to the visible light region. This allows longer wavelength LEDs with lower energy photons to be applied, potentially contributing to avoiding undesired reactions [14–17,34].

**Scheme 2:**
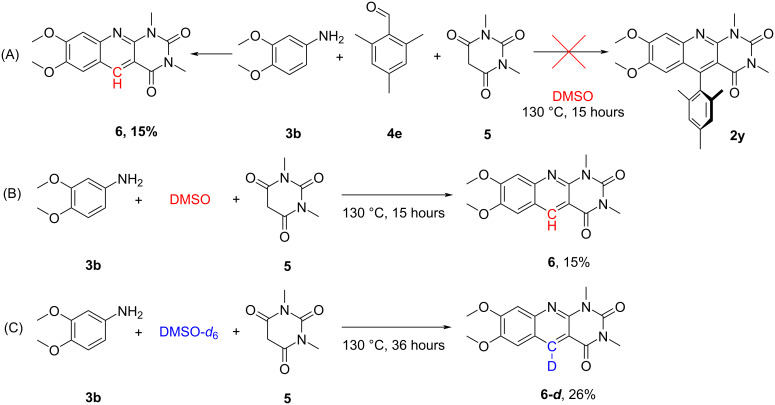
Control experiments related to bulky substituted aldehydes.

When investigating the possibility of the introduction of bulky aldehyde fragments (for example, with mesitaldehyde (**4e**)), we did not observe the formation of 5-aryldeazaalloxazine **2y**; however, we discovered the formation of 5-deazaalloxazine **6** with DMSO as a solvent (Scheme 2A). The unexpected structure of product **6** was confirmed by ^1^H, ^13^C NMR and mass spectrometry. The main feature of the ^1^H NMR spectrum of compound **6** is the absence of the signals of the aromatic aldehyde moiety and the appearance of the singlet of the 5-CH methyne group at 9.56 ppm and the singlets of the protons of the benzene ring of the 5-deazaalloxazine core at 7.64 and 7.71 ppm. To support this theory, we conducted a control experiment between 3,4-dimethoxyaniline (**3b**) and *N*,*N*-dimethylbarbituric acid (**5**) in DMSO. To our delight, 5-deazaalloxazine **6** was formed (Scheme 2B). To prove that DMSO was the methylene source in this reaction, a deuterium labelling experiment was conducted (Scheme 2C). Indeed, the deazaalloxazine derivative **6-*d*** with quantitative incorporation of deuterium in C(5) position, was isolated and confirmed by ^1^H NMR analysis and mass spectrometry (for more details on possible reaction mechanism, see Supporting Information File 1).

Such results with previous reports on DMSO acting as a methine source in the synthesis of heterocyclic compounds [35–36] are opening a new avenue for the green synthesis of non-substituted 5-deazaalloxazines in a pseudo MCR fashion.

## Conclusion

In summary, we have developed a facile, efficient and environmentally friendly method for the synthesis of 5-aryldeazaalloxazine (5-arylpyrimido[4,5-*b*]quinoline-2,4(1*H*,3*H*)-dione) derivatives via three-component reactions in DMSO, with the possibility of varying reaction conditions. Although the yields of the reaction vary from low to moderate, the reaction starts from commercially available substances and leads to valuable compounds. The procedure is low cost and operationally easy with no need for further purification and opens a new route to the synthesis of powerful, phoredox, flavin-like catalysts, as well as potent, biologically active compounds. Interestingly, the introduction of a strong methoxy group at position 7 of the 5-aryldeazaalloxazine core led to a bathochromic shift in the absorption spectra of the synthesised molecules, making them more suitable for visible light photocatalysis.

## Experimental

**Reagents and analytics:** Starting materials were purchased from Sigma-Aldrich and Fluorochem. The solvents were purified and dried using standard procedures. Commercially obtained reagents were used as received without further purification unless otherwise stated. The compound structures were drawn and named using ChemDraw. Nuclear magnetic resonance (NMR) spectra were recorded on an Agilent 400-MR DDR2 (399.94 MHz for ^1^H, 100.58 MHz for ^13^C, 376.50 MHz for ^19^F) or on a JNM-ECZ500R NMR spectrometer, JEOL Resonance, (500.16 MHz for ^1^H, 125.77 MHz for ^13^C, 470.60 MHz for ^19^F) at 298 K unless otherwise indicated. Data for ^1^H NMR are reported as follows: chemical shift (δ ppm), multiplicity (s = singlet, d = doublet, t = triplet, q = quartet, m = multiplet, dd = doublet of doublets, dt = doublet of triplets, br = broad etc.), coupling constant (Hz), and integration. All NMR spectra were processed and assigned using MestreNova. High-resolution mass spectra were obtained on Q-Tof Micro (Waters), equipped with a quadrupole and time-of-flight (TOF) analyser and a multichannel plate (MCP) detector. The melting points were measured on a Boetilus melting point apparatus and are not corrected.

UV–vis spectra were recorded on Agilent Cary 8454 spectrophotometer at 25 °C in analytical-grade DMF. Absorption spectra were processed by using Microsoft Excel and Origin 2018 (OriginLab).

**Typical procedure for the synthesis of 5-aryldeazaalloxazines 2:** An equimolar mixture (3.0 mmol) of the corresponding aniline **3**, aromatic aldehyde **4**, and *N*,*N*-dimethylbarbituric acid (**5**) was dissolved in 6 mL DMSO or DMF/AlCl_3_ (cat.) and heated at 130 °C until a precipitate was formed (ca. 15 h). After completion, the precipitated product was filtered, washed with 2-propanol and dried under vacuum. **2a**: white solid, 43%; mp 325–327 °C; ^1^H NMR (400 MHz, DMSO-*d_6_*) δ 7.82 (s, 1H), 7.55–7.46 (m, 3H), 7.23–7.17 (m, 2H), 6.97 (s, 1H), 3.71 (s, 3H), 3.17 (s, 3H), 2.44 (s, 3H), 2.23 (s, 3H); ^13^C NMR (126 MHz, TFA-*d*) δ 166.0, 159.4, 153.7, 149.7, 146.5, 141.3, 136.1, 133.3, 129.8, 129.4, 128.5, 126.4, 124.1, 118.3, 106.9, 30.5, 28.8, 19.6, 18.1; HRMS (APCI^+^) *m*/*z*: [M + H^+^] calcd for C_21_H_19_N_3_O_2_, 346.1477; found, 346.1547.

## Supporting Information

File 1 Spectroscopic and analytical data.

## Data Availability

All data that supports the findings of this study is available in the published article and/or the supporting information to this article.
