# Supplementary material for: A facile three-component route to powerful 5-aryldeazaalloxazine photocatalysts
Source: Beilstein J Org Chem. 2024 Jul 31;20:1831–8. doi: 10.3762/bjoc.20.161 (PMC11301046; doi:10.3762/bjoc.20.161)
Supplement: File 1 — Spectroscopic and analytical data. [file Beilstein_J_Org_Chem-20-1831-s001.pdf]

BEILSTEIN JOURNAL OF ORGANIC CHEMISTRY

# Supporting Information

for

# A facile three-component route to powerful 5-aryldeazaalloxazine photocatalysts

Ivana Weisheitelová, Radek Cibulka, Marek Sikorski and Tetiana Pavlovska

*Beilstein J. Org. Chem.* **2024,** *20,* 1831–1838. doi:10.3762/bjoc.20.161

# Spectroscopic and analytical data

License and Terms: This is a supporting information file under the terms of the Creative Commons Attribution License (https://creativecommons.org/licenses/by/4.0). Please note that the reuse, redistribution and reproduction in particular requires that the author(s) and source are credited and that individual graphics may be subject to special legal provisions.
The license is subject to the *Beilstein Journal of Organic Chemistry* terms and conditions: (https://www.beilstein-journals.org/bjoc/terms)

## Spectroscopic and analytical data

**Table of contents**

**Reagents and analytics**……………………………………………………………..……..S2

**Optimization of reaction conditions** ………………………………………………..S3

**Synthetic procedures and characterization data for the obtained compounds**..……………………………………………………………………..……S4

**Extended analysis**………………………………………………………….………S16

**NMR spectra of isolated products** ……………........................................………S20

**References**………………………………………………………………….…..S69

**Reagents and analytics:** Starting materials were purchased from Sigma-Aldrich and Fluorochem. The solvents were purified and dried using standard procedures. Commercially obtained reagents were used as received without further purification unless otherwise stated. The compound structures were drawn and named using ChemDraw. Nuclear magnetic resonance (NMR) spectra were recorded in DMSO-$d_6$ and TFA-*d* on an Agilent 400-MR DDR2 (399.94 MHz for $^{1}$H, 100.58 MHz for $^{13}$C, 376.50 MHz for $^{19}$F), or JNM-ECZ500R NMR spectrometer (JEOL Resonance) (500.16 MHz for $^{1}$H, 125.77 MHz for $^{13}$C, 470.60 MHz for $^{19}$F) at 298 K unless otherwise indicated. Data for $^{1}$H NMR are reported as follows: chemical shift (δ ppm), multiplicity (s = singlet, d = doublet, t = triplet, q = quartet, m = multiplet, dd = doublet of doublets, dt = doublet of triplets, br = broad etc.), coupling constant (Hz), and integration. All NMR spectra were processed and assigned using MestreNova. High-resolution mass spectra were obtained on Q-Tof Micro (Waters), equipped with a quadrupole and time-of-flight (TOF) analyser and a multichannel plate (MCP) detector. The melting points were measured on a Boetilus melting point apparatus and are not corrected.

UV–vis spectra were recorded on Agilent Cary 8454 spectrophotometer at 25 °C in analytical-grade DMF. Absorption spectra were processed by using Microsoft Excel and Origin 2018 (OriginLab).

# Optimization of reaction conditions

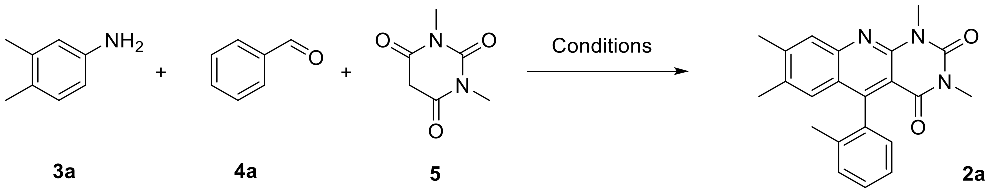


3a 4a 5 2a

| Entry | Solvent | Additive | Volume [mL] | Time [h] | Yield[ %] |
|---|---|---|---|---|---|
| 1 | | – | 2 | 15 h | 11 |
| 2 | | – | 10 | 15 h | 0 |
| 3 | | $Et_3N$ | 2 | 15 h | 8 |
| 4 | DMF | $AlCl_3$ | 2 | 15 h | 41 |
| 5 | 130 °C | ScOTf | 2 | 15 h | 15 |
| 6 | | TMSOTf | 2 | 15 h | 30 |
| 7 | | $AlCl_3$ | 2 | 5 h | 30 |
| 8 | | $AlCl_3$ | 2 | 1 h | 26[a] |
| 9 | | – | 2 | 15 h | 57 |
| 10 | | – | 10 | 15 h | 52[b] |
| 11 | | $Et_3N$ | 2 | 15 h | 25 |
| 12 | | $AlCl_3$ | 2 | 15 h | 55 |
| 13 | | ScOTf | 2 | 15 h | 38 |
| 14 | DMSO | TMSOTf | 2 | 15 h | 48 |
| 15 | 130 °C | – | 2 | 5 h | 8 |
| 16 | | $AlCl_3$ | 2 | 5 h | 26 |
| 17 | | TMSOTf | 2 | 5 h | 20 |
| 18 | | – | 2 | 1 h | 33[a] |
| 19 | | $AlCl_3$ | 2 | 1 h | 39[a] |
| 20 | | TMSOTf | 2 | 1 h | 32[a] |
| 21 | | – | 2 | 15 h | 11 |
| 22 | | – | 10 | 15 h | 6 |
| 23 | | $Et_3N$ | 2 | 15 h | 22 |
| 24 | | $AlCl_3$ | 2 | 15 h | 23 |
| 25 | AcOH | ScOTf | 2 | 15 h | 22 |
| 26 | 110 °C | TMSOTf | 2 | 15 h | 24 |
| 27 | | $H_2SO_4$ | 2 | 15 h | 33 |
| 28 | | PPA | 2 | 15 h | 28 |
| 29 | | $H_2SO_4$ | 2 | 5 h | 26 |
| 30 | | $H_2SO_4$ | 2 | 1 h | 37[a,b] |
| 31 | iPrOH | – | 2 | 15 h | – |
| 32 | 70 °C | – | 10 | 15 h | – |
| 33 | ACN | – | 2 | 15 h | – |
| 34 | 70 °C | – | 10 | 15 h | – |

[a]Reaction done under MW irradiation, [b]The product contaminated with impurities.

# Synthetic procedures and characterization data for the obtained compounds

## Synthesis of 5-aryldeazaalloxazines. General procedure

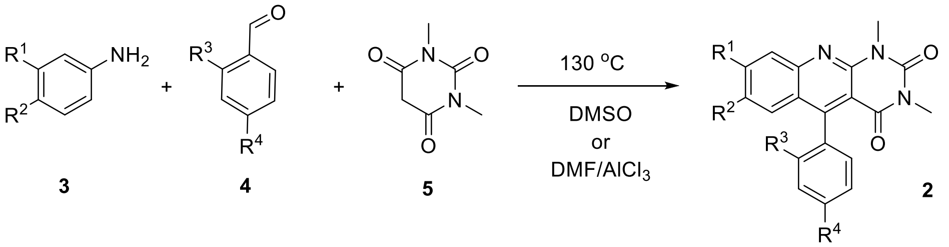


A mixture of substituted aniline **3a**–**h** (1 equiv), aromatic aldehyde **4a**–**d** (1 equiv), and *N,N*-dimethylbarbituric acid (**5**, 1 equiv) was dissolved in dry solvent (6 mL). The solution was heated from 15 hours to 2 days at 130 °C. After cooling mixture to the room temperature, the precipitated product **2** was filtered and washed by 2-propanol.

*1,3,7,8-Tetramethyl-5-phenylpyrimido[4,5-b]quinoline-2,4(1H,3H)-dione* ***(2a)***

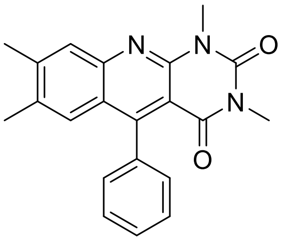

Prepared according to a general procedure from 3,4-dimethylaniline (**3a**, 0.36 g, 3 mmol), benzaldehyde (**4a**, 0.32 g, 3 mmol), and *N,N*-dimethylbarbituric acid (**5**, 0.47 g, 3 mmol) in 6 mL of dry DMSO (reaction time 15 hours). The product was a white crystalline powder with a yield of 43% (0.44 g), m.p.: 325 – 327 °C (from 2-propanol).

$^{1}$H NMR (400 MHz, DMSO-$d_6$) δ 7.82 (s, 1H), 7.55 – 7.46 (m, 3H), 7.23 – 7.17 (m, 2H), 6.97 (s, 1H), 3.71 (s, 3H), 3.17 (s, 3H), 2.44 (s, 3H), 2.23 (s, 3H).

$^{1}$H NMR (500 MHz, TFA-*d*) δ 7.97 (s, 1H), 7.57-7.46 (m, 3H), 7.38 (s, 1H), 7.21 – 7.10 (m, 2H), 3.98 (s, 3H), 3.37 (s, 3H), 2.51 (s, 3H), 2.28 (s, 3H).

$^{13}$C NMR (126 MHz, TFA-*d*) δ 166.0, 159.4, 153.7, 149.7, 146.5, 141.3, 136.1, 133.3, 129.8, 129.4, 128.5, 126.4, 124.1, 118.3, 106.9, 30.5, 28.8, 19.6, 18.1.

HR-MS (APCI+) calculated for $C_{21}H_{19}N_3O_2$ [M+H$^+$]: 346.1555; **observed**: 346.1547.

*5-(2-Bromophenyl)-1,3,7,8-tetramethylpyrimido[4,5-b]quinoline-2,4(1H,3H)-dione* ***(2b)***

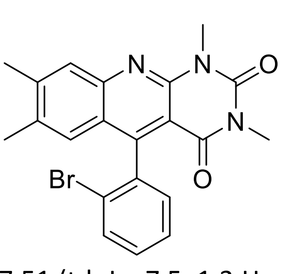


Prepared according to a general procedure from 3,4-dimethylaniline (**3a**, 0.36 g, 3 mmol), 2-brombenzaldehyde (**4b**, 0.56 g, 3 mmol), and *N,N*-dimethylbarbituric acid (**5**, 0.47 g, 3 mmol) in 6 mL of dry DMSO (reaction time 15 hours). The product was a pale yellow crystalline powder with a yield of 59% (0.76 g), m.p.: 289 – 291 °C (from 2-propanol).

$^{1}$H NMR (400 MHz, DMSO-$d_6$) δ 7.82 (s, 1H), 7.77 (dd, J = 7.9, 1.2 Hz, 1H), 7.51 (td, J = 7.5, 1.3 Hz, 1H), 7.43 (td, J = 7.5, 1.8 Hz, 1H), 7.20 (dd, J = 7.5, 1.7 Hz, 1H), 6.86 (d, J = 1.2 Hz, 1H), 3.71 (s, 3H), 3.19 (s, 3H), 2.45 (s, 3H), 2.25 (s, 3H).

$^1$H NMR (500 MHz, TFA-*d*) δ 7.96 (s, 1H), 7.67 (dq, *J* = 8.1, 1.6 Hz, 1H), 7.50 – 7.32 (m, 2H), 7.22 (s, 1H), 7.02 (dt, *J* = 7.6, 1.6 Hz, 1H), 3.94 (s, 3H), 3.35 (s, 3H), 2.48 (s, 3H), 2.25 (s, 3H).

$^{13}$C NMR (126 MHz, TFA-*d*) δ 163.4, 158.8, 154.0, 149.5, 146.5, 141.7, 136.4, 134.7, 132.9, 131.1, 128.4, 127.7, 123.2, 119.5, 118.5, 107.5, 30.5, 28.8, 19.7, 18.2.

HR-MS (APCI+) calculated for $C_{21}H_{18}BrN_3O_2$ [M+H$^+$]: 424.0660; **observed**: 424.0662.

*5-(4-Bromophenyl)-1,3,7,8-tetramethylpyrimido[4,5-b]quinoline-2,4(1H,3H)-dione* ***(2c)***

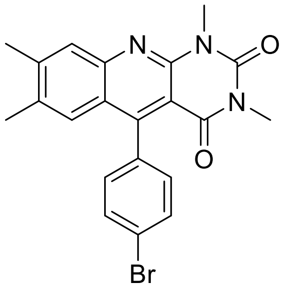


Prepared according to a general procedure from 3,4-dimethylaniline (**3a**, 0.36 g, 3 mmol), 4-brombenzaldehyde (**4c**, 0.56 g, 3 mmol), and *N,N*-dimethylbarbituric acid (**5**, 0.47 g, 3 mmol) in 6 mL of dry DMSO (reaction time 15 hours). The product was a pale yellow crystalline powder with a yield of 59% (0.76 g), m.p.: 285 – 289 °C (from 2-propanol).

$^1$H NMR (400 MHz, DMSO-*d$_6$*) δ 7.82 (s, 1H), 7.68 (d, *J* = 8.4 Hz, 2H), 7.14 (d, *J* = 8.4 Hz, 2H), 7.00 (s, 1H), 3.72 (s, 3H), 3.17 (s, 3H), 2.45 (s, 3H), 2.26 (s, 3H).

$^1$H NMR (500 MHz, TFA-d) δ 7.98 (s, 1H), 7.65 (d, *J* = 8.4 Hz, 2H), 7.39 (s, 1H), 7.03 (d, *J* = 8.6 Hz, 2H), 3.97 (s, 3H), 3.37 (s, 3H), 2.52 (s, 3H), 2.30 (s, 3H).

$^{13}$C NMR (126 MHz, TFA-*d*) δ 164.4, 159.0, 153.9, 149.6, 146.5, 141.5, 136.2, 132.1, 132.0, 128.9, 128.0, 124.4, 123.7, 118.4, 107.1, 30.6, 28.9, 19.7, 18.2.

HR-MS (APCI+) calculated for $C_{21}H_{18}BrN_3O_2$ [M+H$^+$]: 424.0660; **observed**: 424.0665.

*1,3,7,8-Tetramethyl-5-(o-tolyl)pyrimido[4,5-b]quinoline-2,4(1H,3H)-dione* ***(2d)***

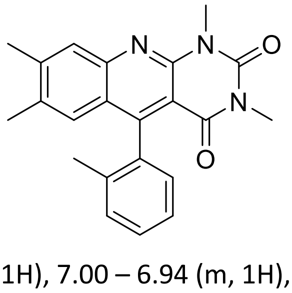


Prepared according to a general procedure from 3,4-dimethylaniline (**3a**, 0.36 g, 3 mmol), 2-methylbenzaldehyde (**4d**, 0.36 g, 3 mmol), and *N,N*-dimethylbarbituric acid (**5**, 0.47 g, 3 mmol) in 6 mL of dry DMSO (reaction time 15 hours). The product was a pale yellow crystalline powder with a yield of 52% (0.57 g), m.p.: 289 – 290 °C (from 2-propanol).

$^1$H NMR (400 MHz, DMSO-*d$_6$*) δ 7.82 (s, 1H), 7.42 – 7.34 (m, 2H), 7.29 (m, 1H), 7.00 – 6.94 (m, 1H), 6.85 (s, 1H), 3.71 (s, 3H), 3.17 (s, 3H), 2.44 (s, 3H), 2.22 (s, 3H), 1.83 (s, 3H).

$^1$H NMR (500 MHz, TFA-*d*) δ 8.00 (s, 1H), 7.43 (td, *J* = 7.6, 1.3 Hz, 1H), 7.36 (d, *J* = 7.7 Hz, 1H), 7.34 – 7.27 (m, 2H), 6.90 (dd, *J* = 7.7, 1.3 Hz, 1H), 3.99 (s, 3H), 3.39 (s, 3H), 2.53 (s, 3H), 2.28 (s, 3H), 1.86 (s, 3H).

$^{13}$C NMR (126 MHz, TFA-*d*) δ 166.4, 159.1, 153.9, 149.7, 146.6, 141.6, 136.2, 134.1, 133.2, 130.4, 129.9, 128.8, 126.1, 125.8, 123.7, 118.4, 107.4, 30.5, 28.8, 19.7, 18.1, 17.8.

HR-MS (APCI+) calculated for $C_{22}H_{21}N_3O_2$ [M+H$^+$]: 360.1712; **observed**: 360.1713.

*7,8-Dimethoxy-1,3-dimethyl-5-phenylpyrimido[4,5-b]quinoline-2,4(1H,3H)-dione* ***(2e)***

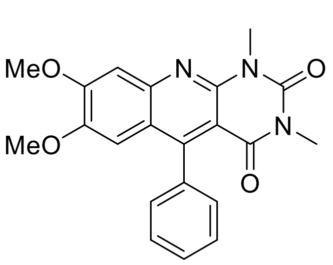

Prepared according to a general procedure from 3,4-dimethoxyaniline (**3b**, 0.46 g, 3 mmol), benzaldehyde (**4a**, 0.32 g, 3 mmol), and *N,N*-dimethylbarbituric acid (**5**, 0.47 g, 3 mmol) in 6 mL of dry DMSO (reaction time 15 hours). The product was a pale yellow crystalline powder with a yield of 59% (0.66 g), m.p.: 308 – 310 °C (from 2-propanol).

$^{1}$H NMR (400 MHz, TFA-*d*) δ 7.62 (s, 1H), 7.59 – 7.49 (m, 3H), 7.22 – 7.14 (m, 2H), 6.87 (s, 1H), 4.09 (s, 3H), 3.99 (s, 3H), 3.69 (s, 3H), 3.38 (s, 3H).

$^{13}$C NMR (101 MHz, TFA-*d*) δ 163.0, 160.2, 159.3, 150.7, 149.8, 145.8, 136.4, 133.6, 129.7, 128.8, 126.2, 121.5, 107.5, 105.8, 98.8, 56.6, 55.2, 30.5, 28.8.

HR-MS (APCI+) calculated for $C_{21}H_{20}N_3O_4$ [M+H$^+$]: 378.1454; **observed**: 378.1448.
Spectra data were in agreement with previously reported values [1]

*5-(2-Bromophenyl)-7,8-dimethoxy-1,3-dimethylpyrimido[4,5-b]quinoline-2,4(1H,3H)-dione* ***(2f)***

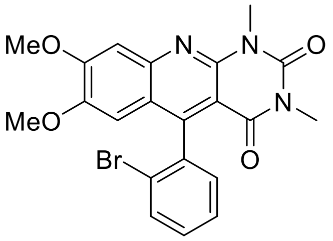

Prepared according to a general procedure from 3,4-dimethoxyaniline (**3b**, 0.46 g, 3 mmol), 2-brombenzaldehyde (**4b**, 0.56 g, 3 mmol), and *N,N*-dimethylbarbituric acid (**5**, 0.47 g, 3 mmol) in 6 mL of dry DMSO (reaction time 15 hours). The product was a pale yellow crystalline powder with a yield of 79% (1.07 g), m.p.: 300 – 302 °C (from 2-propanol).

$^{1}$H NMR (400 MHz, TFA-*d*) δ 7.76 (d, *J* = 9.6 Hz, 1H), 7.64 (s, 1H), 7.55 – 7.39 (m, 2H), 7.10 (d, *J* = 6.9 Hz, 1H), 6.73 (s, 1H), 4.10 (s, 3H), 3.99 (s, 3H), 3.70 (s, 3H), 3.40 (s, 3H).

$^{13}$C NMR (101 MHz, TFA-*d*) δ 160.5, 159.1, 151.2, 1492.8, 145.8, 136.9, 134.9, 133.1, 131.3, 127.9, 127.6, 120.8, 119.6, 106.5, 106.2, 98.9, 56.7, 55.3, 30.6, 28.9.

HR-MS (APCI+) calculated for $C_{21}H_{19}BrN_3O_4$ [M+H$^+$]: 456.0559; **observed**: 456.0553.
Spectra data were in agreement with previously reported values [1]

*5-(4-Bromophenyl)-7,8-dimethoxy-1,3-dimethylpyrimido[4,5-b]quinoline-2,4(1H,3H)-dione* ***(2g)***

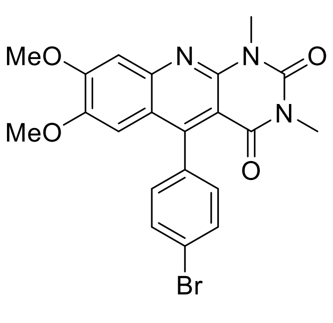

Prepared according to a general procedure from 3,4-dimethoxyaniline (**3b**, 0.46 g, 3 mmol), 4-brombenzaldehyde (**4c**, 0.56 g, 3 mmol), and *N,N*-dimethylbarbituric acid (**5**, 0.47 g, 3 mmol) in 6 mL of dry DMSO (reaction time 15 hours). The product was a pale yellow crystalline powder with a yield of 45% (0.61 g), m.p.: 317 – 319 °C (from 2-propanol).

$^{1}$H NMR (400 MHz, DMSO-*d$_6$*) δ 7.68 (d, J = 8.5, 2H), 7.36 (s, 1H), 7.19 (d, J = 8.3,, 2H), 6.47 (s, 1H), 3.99 (s, 3H), 3.70 (s, 3H), 3.59 (s, 3H), 3.19 (s, 3H).

$^{1}$H NMR (500 MHz, TFA-*d*) δ 7.68 (d, *J* = 8.4, 2H), 7.63 (s, 1H), 7.06 (dt, *J* = 8.4, 1.5 Hz, 2H), 6.87 (s, 1H), 4.09 (s, 3H), 3.97 (s, 3H), 3.73 (s, 3H), 3.38 (s, 3H).

$^{13}C$ NMR (126 MHz, TFA-*d*) δ 161.4, 160.4, 159.2, 151.0, 149.7, 145.8, 136.5, 132.4, 132.2, 127.9, 124.5, 121.1, 107.0, 105.8, 98.9, 56.7, 55.4, 30.5, 28.9.

HR-MS (APCI+) calculated for $C_{21}H_{19}BrN_3O_4$ [M+H$^+$]: 456.0559; **observed**: 456.0559.

*7,8-Dimethoxy-1,3-dimethyl-5-(o-tolyl)pyrimido[4,5-b]quinoline-2,4(1H,3H)-dione* ***(2h)***

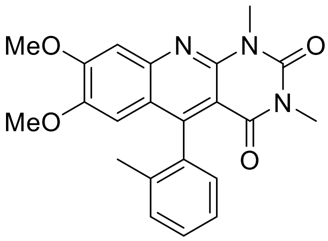


Prepared according to a general procedure from 3,4-dimethoxyaniline (**3b**, 0.46 g, 3 mmol), 2-methylbenzaldehyde (**4d**, 0.36 g, 3 mmol), and *N,N*-dimethylbarbituric acid (**5**, 0.47 g, 3 mmol) in 6 mL of dry DMSO (reaction time 15 hours). The product was a pale yellow crystalline powder with a yield of 62% (0.73 g), m.p.: 287 – 290 °C (from 2-propanol).

$^1H$ NMR (400 MHz, DMSO-*d*$_6$) δ 7.42 – 7.34 (m, 3H), 7.30-7.25 (m, 1H), 7.00 (d, *J* = 7.4 Hz, 1H), 6.32 (s, 1H), 3.99 (s, 3H), 3.71 (s, 3H), 3.51 (s, 3H), 3.17 (s, 3H), 1.85 (s, 3H).

$^1H$ NMR (500 MHz, TFA-*d*) δ 7.63 (s, 1H), 7.44 (td, *J* = 7.6, 1.3 Hz, 1H), 7.38 (d, *J* = 7.7 Hz, 1H), 7.33 (t, *J* = 7.5 Hz, 1H), 6.93 (dd, *J* = 7.7, 1.3 Hz, 1H), 6.74 (s, 1H), 4.09 (s, 3H), 3.98 (s, 3H), 3.66 (s, 3H), 3.39 (s, 3H), 1.88 (s, 3H).

$^{13}C$ NMR (126 MHz, TFA-*d*) δ 163.3, 160.3, 159.2, 151.0, 149.9, 145.9, 136.4, 134.0, 133.4, 130.5, 130.0, 126.4, 125.7, 121.1, 106.9, 106.1, 98.9, 56.6, 55.3, 30.5, 28.8, 17.7.

HR-MS (APCI+) calculated for $C_{22}H_{21}N_3O_4$ [M+H$^+$]: 392.1610; **observed**: 392.1601.

*7-Methoxy-1,3-dimethyl-5-phenylpyrimido[4,5-b]quinoline-2,4(1H,3H)-dione* ***(2i)***

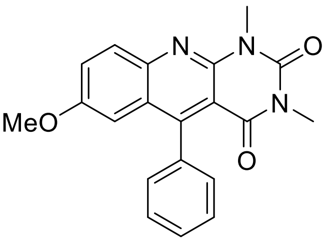


Prepared according to a general procedure from 4-methoxyaniline (**3c**, 0.37 g, 3 mmol), benzaldehyde (**4a**, 0.32 g, 3 mmol), and *N,N*-dimethylbarbituric acid (**5**, 0.47 g, 3 mmol) in 6 mL of dry DMF with catalytic amount of $AlCl_3$ (reaction time 15 hours). The product was a yellow crystalline powder with a yield of 41% (0.43 g), m.p.: 302 – 305 °C (from 2-propanol).

$^1H$ NMR (400 MHz, DMSO-*d*$_6$) δ 7.92 (d, *J* = 9.3 Hz, 1H), 7.56 – 7.44 (m, 4H), 7.22 (d, *J* = 2.7 Hz, 2H), 6.49 (d, *J* = 2.9 Hz, 1H), 3.70 (s, 3H), 3.59 (s, 3H), 3.17 (s, 3H).

$^1H$ NMR (500 MHz, TFA-*d*) δ 8.18 (d, *J* = 9.3 Hz, 1H), 7.78 (dd, *J* = 9.3, 2.7 Hz, 1H), 7.65 – 7.52 (m, 3H), 7.24 – 7.15 (m, 2H), 6.91 (d, *J* = 2.8 Hz, 1H), 4.00 (s, 3H), 3.65 (s, 3H), 3.40 (s, 3H).

$^{13}C$ NMR (126 MHz, TFA-*d*) δ 165.2, 160.1, 159.0, 149.6, 145.6, 133.4, 132.7, 131.0, 130.0, 128.8, 127.3, 126.2, 120.3, 108.4, 108.3, 54.8, 30.5, 28.9.

HR-MS (APCI+) calculated for $C_{20}H_{17}N_3O_3$ [M+H$^+$]: 348.1348; **observed**: 348.1345.

*5-(2-Bromophenyl)-7-methoxy-1,3-dimethylpyrimido[4,5-b]quinoline-2,4(1H,3H)-dione* ***(2j)***

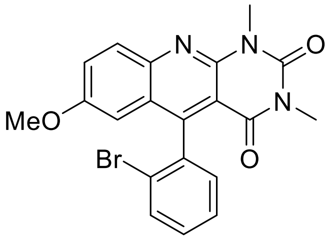

Prepared according to a general procedure from 4-methoxyaniline (**3c**, 0.37 g, 3 mmol), 2-brombenzaldehyde (**4b**, 0.56 g, 3 mmol), and *N,N*-dimethylbarbituric acid (**5**, 0.47 g, 3 mmol) in and 6 mL of dry DMF with catalytic amount of $AlCl_3$ (reaction time 15 hours). The product was a yellow crystalline powder with a yield of 45% (0.58 g), m.p.: 205 – 207 °C (from 2-propanol).

$^1$H NMR (400 MHz, DMSO-$d_6$) δ 7.97 (d, *J* = 9.2 Hz, 1H), 7.79 (dd, *J* = 8.0, 1.2 Hz, 1H), 7.60 (dd, *J* = 9.2, 2.8 Hz, 1H), 7.53 (td, *J* = 7.5, 1.2 Hz, 1H), 7.44 (td, *J* = 7.7, 1.8 Hz, 1H), 7.23 (dd, *J* = 7.5, 1.7 Hz, 1H), 6.38 (d, *J* = 2.8 Hz, 1H), 3.71 (s, 3H), 3.60 (s, 3H), 3.31 (s, 3H).

$^1$H NMR (500 MHz, TFA-*d*) δ 8.28 (dd, *J* = 9.4, 1.1 Hz, 1H), 7.91 – 7.80 (m, 2H), 7.60 (td, *J* = 7.6, 1.2 Hz, 1H), 7.53 (td, *J* = 7.8, 1.5 Hz, 1H), 7.19 (dd, *J* = 7.6, 1.5 Hz, 1H), 6.86 (d, *J* = 2.5 Hz, 1H), 4.07 (s, 3H), 3.74 (s, 3H), 3.50 (s, 3H).

$^{13}$C NMR (126 MHz, TFA-*d*) δ 162.7, 160.4, 158.7, 149.5, 145.6, 134.7, 133.2, 133.0, 131.3, 131.3, 128.0, 127.5, 126.4, 120.5, 119.4, 117.7, 115.4, 113.2, 110.9, 109.0, 107.3, 54.9, 30.5, 28.9.

HR-MS (APCI+) calculated for $C_{20}H_{16}BrN_3O_3$ [M+H$^+$]: 426.0453; **observed**: 426.0452.

*5-(4-Bromophenyl)-7-methoxy-1,3-dimethylpyrimido[4,5-b]quinoline-2,4(1H,3H)-dione* ***(2k)***

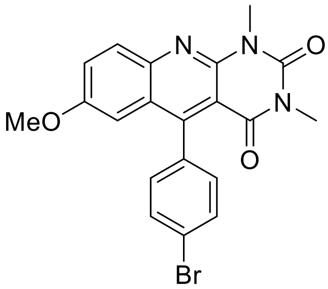

Prepared according to a general procedure from 4-methoxyaniline (**3c**, 0.37 g, 3 mmol), 4-brombenzaldehyde (**4c**, 0.56 g, 3 mmol), and *N,N*-dimethylbarbituric acid (**5**, 0.47 g, 3 mmol) in 6 mL of dry DMF with catalytic amount of $AlCl_3$ (reaction time 15 hours). The product was a pale yellow crystalline powder with a yield of 45% (0.58 g), m.p.: 280 - 282 °C (from 2-propanol).

$^1$H NMR (400 MHz, DMSO-$d_6$) δ 7.96 (d, *J* = 9.2 Hz, 1H), 7.70 (d, *J* = 8.5 Hz, 2H), 7.57 (dd, *J* = 9.3, 2.9, Hz, 1H), 7.20 (d, *J* = 7.7 Hz, 2H), 6.53 (d, *J* = 2.8 Hz, 1H), 3.70 (s, 3H), 3.64 (s, 3H), 3.18 (s, 3H).

$^1$H NMR (500 MHz, TFA-*d*) δ 8.25 (d, *J* = 9.3 Hz, 1H), 7.86 (dd, *J* = 9.4, 2.7 Hz, 1H), 7.84 – 7.72 (m, 2H), 7.20 – 7.12 (m, 2H), 6.98 (d, *J* = 2.7 Hz, 1H), 4.05 (s, 3H), 3.76 (s, 3H), 3.47 (s, 3H).

$^{13}$C NMR (126 MHz, TFA-*d*) δ 163.6, 160.2, 158.9, 149.4, 145.5, 132.7, 132.3, 132.2, 131.0, 127.8, 126.8, 124.6, 120.5, 108.5, 107.9, 54.9, 30.5, 28.9.

HR-MS (APCI+) calculated for $C_{20}H_{16}BrN_3O_3$ [M+H$^+$]: 426.0453; **observed**: 426.0452.

*7-Methoxy-1,3-dimethyl-5-(o-tolyl)pyrimido[4,5-b]quinoline-2,4(1H,3H)-dione* ***(2l)***

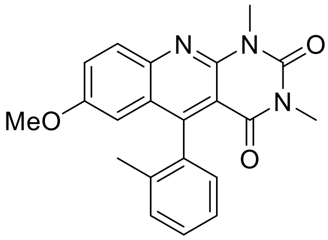

Prepared according to a general procedure from 3-methoxyaniline (**3c**, 0.37 g, 3 mmol), 2-methylbenzaldehyde (**4d**, 0.36 g, 3 mmol), and *N,N*-dimethylbarbituric acid (**5**, 0.47 g, 3 mmol) in 6 mL of dry DMSO (reaction time 15 hours). The product was a pale yellow crystalline powder with a yield of 44% (0.48 g), m.p.: 209 – 210 °C (from 2-propanol).

$^1$H NMR (400 MHz, DMSO-$d_6$) δ 7.96 (d, *J* = 9.2 Hz, 1H), 7.54 (dd, *J* = 9.2, 2.9 Hz, 1H), 7.38-7.30 (m, 2H), 7.30 – 7.26 (m, 1H), 6.97 (d, *J* = 7.1 Hz, 1H), 6.38 (d, *J* = 2.9 Hz, 1H), 3.72 (s, 3H), 3.58 (s, 3H), 3.19 (s, 3H), 1.85 (s, 3H).

$^1$H NMR (500 MHz, TFA-*d*) δ 8.16 (dd, *J* = 9.2, 1.0 Hz, 1H), 7.76 (ddd, *J* = 9.3, 2.7, 1.1 Hz, 1H), 7.44 (dd, *J* = 8.3, 7.0 Hz, 1H), 7.37 (d, *J* = 7.7 Hz, 1H), 7.32 (t, *J* = 7.5 Hz, 1H), 6.91 (dd, *J* = 7.7, 1.4 Hz, 1H), 6.78 – 6.74 (m, 1H), 3.97 (s, 3H), 3.61 (s, 3H), 3.38 (s, 3H), 1.87 (s, 3H).

$^{13}$C NMR (126 MHz, TFA-*d*) δ 165.6, 160.3, 158.9, 149.6, 145.6, 133.9, 133.1, 132.7, 131.1, 130.5, 130.1, 126.8, 126.4, 125.6, 120.5, 108.7, 107.6, 54.8, 30.5, 28.9, 17.7.

HR-MS (APCI+) calculated for $C_{21}H_{19}N_3O_3$ [M+H$^+$]: 362.1504; **observed**: 362.1507.

*8-Methoxy-1,3-dimethyl-5-phenylpyrimido[4,5-b]quinoline-2,4(1H,3H)-dione* ***(2m)***

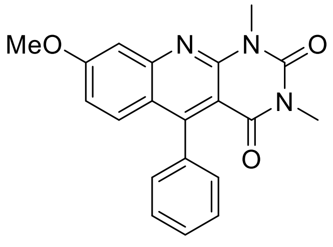


Prepared according to a general procedure from 3-methoxyaniline (**3d**, 0.37 g, 3 mmol), benzaldehyde (**4a**, 0.32 g, 3 mmol), and *N,N*-dimethylbarbituric acid (**5**, 0.47 g, 3 mmol) in 6 mL of dry DMSO (reaction time 2 days). After completion the product was precipitated by 2-propanol and then filtered. The product was a white crystalline powder with a yield of 38% (0.40 g), m.p.: 326 – 327 °C (from 2-propanol).

$^1$H NMR (400 MHz, DMSO-$d_6$) δ 7.53 – 7.41 (m, 3H), 7.32 (d, J = 2.7 Hz, 1H), 7.22 – 7.15 (m, 2H), 7.15 – 7.04 (m, 2H), 3.94 (s, 3H), 3.69 (s, 3H), 3.15 (s, 3H).

$^1$H NMR (500 MHz, TFA-*d*) δ 7.54 – 7.44 (m, 6H), 7.22 (dd, *J* = 9.5, 2.4 Hz, 1H), 7.15 – 7.09 (m, 2H), 3.97 (s, 3H), 3.96 (s, 3H), 3.35 (s, 3H).

$^{13}$C NMR (126 MHz, TFA-*d*) δ 169.4, 165.3, 159.2, 149.8, 147.4, 140.7, 133.3, 132.3, 129.8, 128.5, 126.4, 121.2, 120.42, 105.0, 99.0, 55.9, 30.5, 28.8.

HR-MS (APCI+) calculated for $C_{20}H_{17}N_3O_3$ [M+H$^+$]: 348.1348; **observed**: 348.1349.

*5-(2-Bromophenyl)-8-methoxy-1,3-dimethylpyrimido[4,5-b]quinoline-2,4(1H,3H)-dione* ***(2n)***

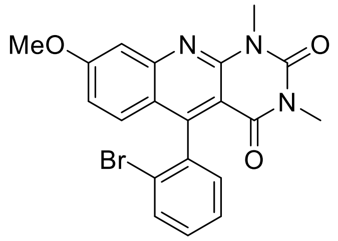


Prepared according to a general procedure from 3-methoxyaniline (**3d**, 0.37 g, 3 mmol), 2-brombenzaldehyde (**4b**, 0.56 g, 3 mmol), and *N,N*-dimethylbarbituric acid (**5**, 0.47 g, 3 mmol) in 6 mL of dry DMSO (reaction time 2 days). After completion the product was precipitated by 2-propanol and then filtered. The product was a white crystalline powder with a yield of 41% (0.53 g), m.p.: 282 – 284 °C (from 2-propanol).

$^1$H NMR (400 MHz, DMSO-$d_6$) δ 7.77 (dd, *J* = 8.0, 1.2 Hz, 1H), 7.51 (dd, *J* = 7.5, 1.3 Hz, 1H), 7.45 (dd, *J* = 7.9, 1.8 Hz, 1H), 7.38 (d, *J* = 2.5 Hz, 1H), 7.23 (dd, *J* = 7.5, 1.7 Hz, 1H), 7.14 (dd, *J* = 9.3, 2.5 Hz, 1H), 7.04 (d, *J* = 9.3 Hz, 1H), 3.97 (s, 3H), 3.73 (s, 3H), 3.19 (s, 3H).

$^1$H NMR (500 MHz, TFA-*d*) δ 7.69 (dt, *J* = 8.1, 1.3 Hz, 1H), 7.53 (t, *J* = 2.0 Hz, 1H), 7.45 (tt, *J* = 7.6, 1.4 Hz, 1H), 7.42 – 7.35 (m, 2H), 7.25 (dt, *J* = 9.5, 2.0 Hz, 1H), 7.05 (dt, *J* = 7.6, 1.6 Hz, 1H), 4.01 (s, 3H), 3.97 (s, 3H), 3.38 (s, 3H).

$^{13}C$ NMR (126 MHz, TFA-*d*) δ 169.7, 162.9, 159.0, 149.7, 147.5, 141.1, 134.6, 133.0, 131.4, 131.2, 127.7, 121.7, 119.8, 119.6, 105.5, 99.2, 56.0, 30.6, 28.8.

HR-MS (APCI+) calculated for $C_{20}H_{16}BrN_3O_3$ [M+H$^+$]: 426.0453; **observed**: 426.0452.

*5-(4-Bromophenyl)-8-methoxy-1,3-dimethylpyrimido[4,5-b]quinoline-2,4(1H,3H)-dione **(2o)***

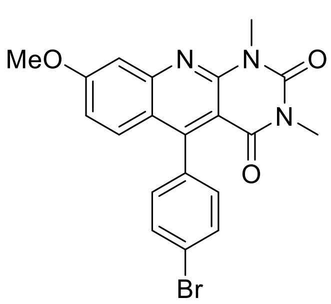


Prepared according to a general procedure from 3-methoxyaniline (**3d,** 0.37 g, 3 mmol), 4-brombenzaldehyde (**4c**, 0.56 g, 3 mmol), and *N,N*-dimethylbarbituric acid (**5**, 0.47 g, 3 mmol) in 6 mL of dry DMSO (reaction time 2 days). After completion the product was precipitated by 2-propanol and then filtered. The product was a white crystalline powder with a yield of 38% (0.49 g), m.p.: 262 – 264 °C (from 2-propanol).

$^1H$ NMR (400 MHz, DMSO-*d*$_6$) δ 7.68 (d, *J* = 8.9 Hz, 2H), 7.35 (d, *J* = 2.5 Hz, 1H), 7.22 – 7.07 (m, 4H), 3.96 (s, 3H), 3.71 (s, 3H), 3.17 (s, 3H).

$^1H$ NMR (500 MHz, TFA-*d*) δ 7.77 (d, *J* = 7.7 Hz, 2H), 7.67 – 7.61 (m, 2H), 7.37 (dd, *J* = 9.5, 2.4 Hz, 1H), 7.13 (d, *J* = 8.0 Hz, 2H), 4.11 (s, 3H), 4.09 (s, 3H), 3.49 (s, 3H).

$^{13}C$ NMR (126 MHz, TFA-*d)* δ 169.6, 163.8, 159.2, 149.7, 147.5, 140.8 132.1, 132.0, 131.8, 128.0, 124.5, 121.5, 120.1, 105.1, 99.2, 56.0, 30.6, 28.8.

HR-MS (APCI+) calculated for $C_{20}H_{16}BrN_3O_3$ [M+H+]: 426.0453; observed: 426.0453.

*8-Methoxy-1,3-dimethyl-5-(o-tolyl)pyrimido[4,5-b]quinoline-2,4(1H,3H)-dione **(2p)***

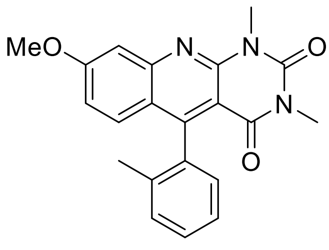


Prepared according to a general procedure from 3-methoxyaniline (**3d**, 0.37 g, 3 mmol), 2-methylbenzaldehyde (**4d**, 0.36 g, 3 mmol), and *N,N*-dimethylbarbituric acid (**5**, 0.47 g, 3 mmol) in 6 mL of dry DMSO (reaction time 2 days). After completion the product was precipitated by 2-propanol and then filtered. The product was a white crystalline powder with a yield of 32% (0.35 g), m.p.: 284 – 287 °C (from 2-propanol).

$^1H$ NMR (400 MHz, DMSO-*d*$_6$) δ 7.41 – 7.24 (m, 4H), 7.13 – 7.06 (m, 1H), 7.04 – 6.95 (m, 2H), 3.96 (s, 1H), 3.73 (s, 3H), 3.18 (s, 3H), 1.83 (s, 3H).

$^1H$ NMR (500 MHz, TFA-*d*) δ 7.56 (d, *J* = 2.3 Hz, 1H), 7.43 (dd, *J* = 8.7, 2.6 Hz, 2H), 7.36 (d, *J* = 7.7 Hz, 1H), 7.32 (t, *J* = 7.5 Hz, 1H), 7.26 (dd, *J* = 9.5, 2.4 Hz, 1H), 6.92 (dd, *J* = 7.7, 1.3 Hz, 1H), 4.02 (s, 3H), 4.01 (s, 3H), 3.41 (s, 3H), 1.89 (s, 3H).

$^{13}C$ NMR (126 MHz, TFA-*d*) δ 169.5, 165.6, 159.2, 149.8, 147.5, 140.8, 134.0, 133.1, 131.8, 130.4, 129.9, 126.1, 125.9, 121.5, 120.1, 105.4, 99.1, 56.0, 30.5, 28.8, 17.7.

HR-MS (APCI+) calculated for $C_{21}H_{19}N_3O_3$ [M+H$^+$]: 362.1504; observed: 362.1505.

*8-Bromo-1,3-dimethyl-5-phenylpyrimido[4,5-b]quinoline-2,4(1H,3H)-dione* ***(2q)***

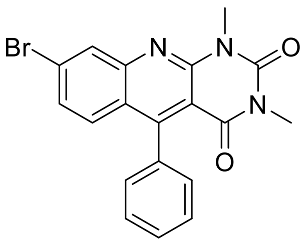

Prepared according to a general procedure from 3-bromoaniline (**3e**, 0.52 g, 3 mmol), benzaldehyde (**4a**, 0.32 g, 3 mmol), and *N,N*-dimethylbarbituric acid (**5**, 0.47 g, 3 mmol) in 6 mL of dry DMSO (reaction time 2 days). The product was a beige crystalline powder with a yield of 14% (0.17 g), m.p.: 284 – 286 °C (from 2-propanol).

$^{1}H$ NMR (400 MHz, DMSO-$d_6$) δ 8.20 (d, *J* = 2.1 Hz, 1H), 7.61 (dd, *J* = 9.0, 2.0 Hz, 1H), 7.50 (qd, *J* = 4.9, 1.8 Hz, 3H), 7.25 – 7.18 (m, 2H), 7.15 (d, *J* = 9.0 Hz, 1H), 3.69 (s, 3H), 3.16 (s, 3H).

$^{1}H$ NMR (500 MHz,TFA-*d*) δ 8.46 (s, 1H), 7.83 – 7.79 (m, 1H), 7.63 – 7.54 (m, 3H), 7.49 (d, *J* = 9.1 Hz, 1H), 7.21 – 7.17 (m, 2H), 4.03 (s, 3H), 3.41 (s, 3H).

$^{13}C$ NMR (126 MHz, TFA-*d*) δ 167.0, 158.7, 149.3, 148.1, 137.3, 135.8, 133.3, 132.7, 131.2, 130.2, 128.8, 126.3, 124.0, 121.8, 108.3, 30.8, 28.9.

HR-MS (APCI+) calculated for $C_{19}H_{14}BrN_3O_2$ [M+H$^+$]: 396.0347; **observed**: 396.0353.

*8-Bromo-5-(2-bromophenyl)-1,3-dimethylpyrimido[4,5-b]quinoline-2,4(1H,3H)-dione (**2r**)*

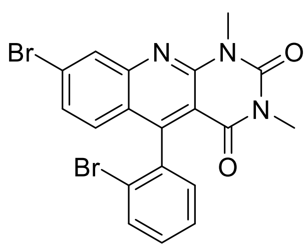

Prepared according to a general procedure from 3-bromoaniline (**3e**, 0.52 g, 3 mmol), 2-brombenzaldehyde (**4b**, 0.56 g, 3 mmol), and *N,N*-dimethylbarbituric acid (**5**, 0.47 g, 3 mmol) in 6 mL of dry DMSO (reaction time 15 hours). The product was a beige crystalline powder with a yield of 23% (0.33 g), m.p.: 277 – 279 °C (from 2-propanol).

$^{1}H$ NMR (400 MHz, DMSO-$d_6$) δ 8.28 – 8.23 (m, 1H), 7.78 (dd, *J* = 8.0, 1.2 Hz, 1H), 7.64 (dd, *J* = 9.0, 2.0 Hz, 1H), 7.58 – 7.51 (m, 1H), 7.44 (td, *J* = 7.7, 1.8 Hz, 1H), 7.23 (dd, *J* = 7.5, 1.8 Hz, 1H), 7.07 (d, *J* = 9.0 Hz, 1H),, 3.72 (s, 3H), 3.20 (s, 3H).

$^{1}H$ NMR (500 MHz,TFA-*d*) δ 8.48 (s, 1H), 7.78 (ddt, *J* = 25.6, 8.0, 1.4 Hz, 2H), 7.55 – 7.42 (m, 2H), 7.36 (dd, *J* = 9.0, 1.1 Hz, 1H), 7.09 (dt, *J* = 7.6, 1.4 Hz, 1H), 4.02 (s, 3H), 3.42 (s, 3H).

$^{13}C$ NMR (126 MHz, TFA-*d*) δ 164.5, 158.4, 149.2, 148.1, 137.6, 136.0, 134.1, 133.7, 133.1, 131.5, 130.2, 127.9, 127.6, 123.2, 122.0, 119.3, 108.9, 30.8, 28.9.

HR-MS (APCI+) calculated for $C_{19}H_{13}Br_2N_3O_2$ [M+H$^+$]: 473.9453; **observed**: 473.9457.

*8-Bromo-1,3-dimethyl-5-(o-tolyl)pyrimido[4,5-b]quinoline-2,4(1H,3H)-dione* ***(2s)***

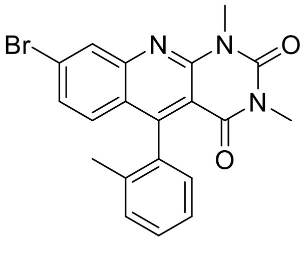

Prepared according to a general procedure from 3-bromoaniline (**3e**, 0.52 g, 3 mmol), 2-methylbenzaldehyde (**4d**, 0.36 g, 3 mmol), and *N,N*-dimethylbarbituric acid (**5**, 0.47 g, 3 mmol) in 6 mL of dry DMSO (reaction time 2 days). The product was a white crystalline powder with a yield of 28% (0.34 g), m.p.: 282 – 285 °C (from 2-propanol).

$^{1}H$ NMR (400 MHz, DMSO-$d_6$) δ 8.22 (d, *J* = 2.0 Hz, 1H), 7.59 (dd, *J* = 9.0, 2.0 Hz, 1H), 7.41 – 7.33 (m, 2H), 7.29 (td, *J* = 7.0, 2.0 Hz, 1H), 7.04 (d, *J* = 9.0 Hz, 1H), 7.00 (d, *J* = 7.4 Hz, 1H), 3.72 (s, 3H), 3.18 (s, 3H), 1.84 (s, 3H).

$^{1}H$ NMR (500 MHz, TFA-*d*) δ 8.47 (s, 1H), 7.78 (dt, *J* = 9.0, 1.7 Hz, 1H), 7.47 (tt, *J* = 7.7, 1.5 Hz, 1H), 7.43 – 7.29 (m, 3H), 6.92 (dd, *J* = 7.7, 1.5 Hz, 1H), 4.02 (s, 3H), 3.41 (s, 3H), 1.89 (s, 3H).

$^{13}C$ NMR (126 MHz, TFA-*d*) δ 167.4, 158.6, 149.3, 148.1, 137.4, 135.9, 134.0, 133.6, 132.5, 130.6, 130.6, 130.2, 126.3, 125.7, 123.6, 121.9, 108.7, 30.8, 28.9, 17.8.

HR-MS (APCI+) calculated for $C_{20}H_{16}BrN_3O_2$ [M+H$^+$]: 410.0504; **observed**: 410.0495.

*8-Chloro-1,3-dimethyl-5-phenylpyrimido[4,5-b]quinoline-2,4(1H,3H)-dione **(2t)***

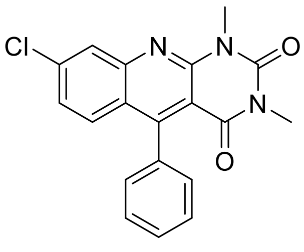


Prepared according to a general procedure from 3-chloroaniline (**3f**, 0.38 g, 3 mmol), benzaldehyde (**4a**, 0.32 g, 3 mmol), and *N,N*-dimethylbarbituric acid (**5**, 0.47 g, 3 mmol) in 6 mL of dry DMSO (reaction time 2 days). The product was a pale yellow crystalline powder with a yield of 15% (0.16 g), m.p.: 286 – 288 °C (from 2-propanol).

$^{1}H$ NMR (400 MHz, DMSO-*d*$_6$) δ 8.06 (d, *J* = 2.2 Hz, 1H), 7.53-7.46 (m, 4H), 7.26 (s, 1H), 7.24 (s, 2H), 3.71 (s, 3H), 3.18 (s, 3H).

$^{1}H$ NMR (500 MHz,TFA-*d*) δ 8.27 (d, *J* = 1.8 Hz, 1H), 7.64 (dd, *J* = 9.1, 1.9 Hz, 1H), 7.63 – 7.53 (m, 4H), 7.20 – 7.16 (m, 2H), 4.02 (s, 3H), 3.41 (s, 3H).

$^{13}C$ NMR (126 MHz, TFA-*d*) δ 166.8, 158.7, 149.2, 148.2, 147.2, 137.6, 132.7, 131.6, 130.5, 130.2, 128.8, 126.3, 123.7, 118.5, 108.2, 30.7, 28.9.

HR-MS (APCI+) calculated for $C_{19}H_{14}ClN_3O_2$ [M+H$^+$]: 352.0853; **observed**: 352.0848.

*8-Chloro-1,3-dimethyl-5-(o-tolyl)pyrimido[4,5-b]quinoline-2,4(1H,3H)-dione **(2u)***

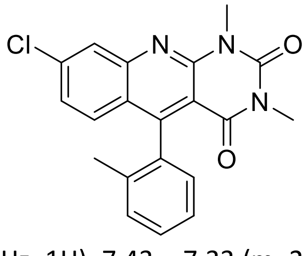


Prepared according to a general procedure from 3-chloroaniline (**3f**, 0.38 g, 3 mmol), 2-methylbenzaldehyde (**4d**, 0.36 g, 3 mmol), and *N,N*-dimethylbarbituric acid (**5**, 0.47 g, 3 mmol) in 6 mL of dry DMSO (reaction time 2 days). The product was a white crystalline powder with a yield of 37% (0.40 g), m.p.: 265 – 267 °C (from 2-propanol).

$^{1}H$ NMR (400 MHz, DMSO-*d*$_6$) δ 8.05 (d, *J* = 2.1 Hz, 1H), 7.48 (dd, *J* = 9.0, 2.2 Hz, 1H), 7.43 – 7.33 (m, 2H), 7.29 (td, *J* = 7.1, 2.0 Hz, 1H), 7.11 (d, *J* = 9.0 Hz, 1H), 6.99 (dd, *J* = 7.2, 1.2 Hz, 1H), 3.70 (s, 3H), 3.16 (s, 3H), 1.82 (s, 3H).

$^{1}H$ NMR (500 MHz, TFA-*d*) δ 8.30 (d, *J* = 1.7 Hz, 1H), 7.64 (dt, *J* = 9.1, 1.6 Hz, 1H), 7.53 – 7.47 (m, 2H), 7.45 – 7.30 (m, 2H), 6.94 (dd, *J* = 7.8, 1.4 Hz, 1H), 4.03 (s, 3H), 3.42 (s,3H), 1.90 (s, 3H).

$^{13}C$ NMR (126 MHz, TFA-*d*) δ 167.2, 158.6, 149.3, 148.2, 147.3, 137.6, 134.0, 132.6, 131.0, 130.7, 130.5, 130.2, 126.3, 125.7, 123.3, 118.6, 108.6, 30.7, 28.9, 17.8.

HR-MS (APCI+) calculated for $C_{20}H_{16}ClN_3O_2$ [M+H$^+$]: 366.1009; **observed**: 366.1014.

*8-Fluoro-1,3-dimethyl-5-phenylpyrimido[4,5-b]quinoline-2,4(1H,3H)-dione **(2v)***

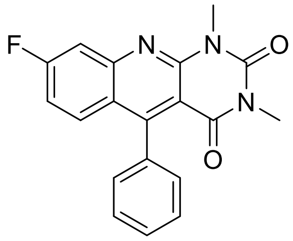

Prepared according to a general procedure from 3-fluoroaniline (**3g**, 0.33 g, 3 mmol), benzaldehyde (**4a**, 0.32 g, 3 mmol), and *N,N*-dimethylbarbituric acid (**5**, 0.47 g, 3 mmol) in 6 mL of dry DMSO (reaction time 2 days). The product was a white crystalline powder with a yield of 10% (0.10 g) and contains 4% of impurities that could not be removed, m.p.: 273 – 275 °C (from 2-propanol).

$^{1}$H NMR (400 MHz, DMSO-$d_6$) δ 7.73 (dd, *J* = 10.3, 2.6 Hz, 1H), 7.54 – 7.46 (m, 3H), 7.38 (ddd, *J* = 9.4, 8.4, 2.6 Hz, 1H), 7.29 (dd, *J* = 9.4, 6.4 Hz, 1H), 7.24 – 7.19 (m, 2H), 3.70 (s, 3H), 3.16 (s, 3H).

$^{1}$H NMR (500 MHz, TFA-*d*) δ 7.96 (dt, *J* = 8.2, 2.5 Hz, 1H), 7.71 (ddd, *J* = 9.4, 5.3, 2.1 Hz, 1H), 7.67 – 7.55 (m, 3H), 7.43 (ddt, *J* = 9.8, 7.7, 2.2 Hz, 1H), 7.23 – 7.15 (m, 2H), 4.02 (s, 3H), 3.41 (s, 3H).

$^{13}$C NMR (126 MHz, TFA-*d*) δ 170.1, 167.8, 166.2, 158.8, 149.3, 148.4, 139.3 (d, *J* = 13.9 Hz), 134.1 (d, *J* = 11.5 Hz), 132.9, 130.2, 128.7, 126.3, 122.4, 119.5 (d, *J* = 24.5 Hz), 107.5, 105.2 (d, *J* = 27.3 Hz), 30.7, 28.9.

$^{19}$F NMR (471 MHz, TFA-*d*) δ -78.01.

HR-MS (APCI+) calculated for $C_{19}H_{14}FN_3O_2$ [M+H$^+$]: 336.1148; **observed**: 336.1149.

*8-Fluoro-1,3-dimethyl-5-(o-tolyl)pyrimido[4,5-b]quinoline-2,4(1H,3H)-dione **(2w)***

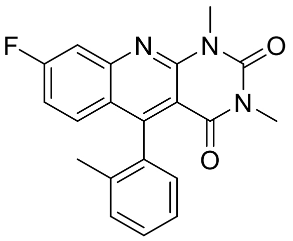

Prepared according to a general procedure from 3-fluoroaniline (**3g**, 0.33 g, 3 mmol), 2-methylbenzaldehyde (**4d**, 0.36 g, 3 mmol), and *N,N*-dimethylbarbituric acid (**5**, 0.47 g, 3 mmol) in 6 mL of dry DMSO (reaction time 2 days). The product was a beige crystalline powder with a yield of 19% (0.20 g), m.p.: 261 – 263 °C (from 2-propanol).

$^{1}$H NMR (400 MHz, DMSO-$d_6$) δ 7.76 (dd, *J* = 10.3, 2.6 Hz, 1H), 7.45 – 7.34 (m, 3H), 7.33 – 7.27 (m, 1H), 7.21 – 7.16 (m, 1H), 7.00 (d, *J* = 7.4 Hz, 1H), 3.72 (s, 3H), 3.18 (s, 3H), 1.84 (s, 3H).

$^{1}$H NMR (500 MHz, TFA-*d*) δ 7.93 (dt, *J* = 8.2, 2.3 Hz, 1H), 7.57 (ddd, *J* = 9.7, 5.3, 1.9 Hz, 1H), 7.46 – 7.40 (m, 1H), 7.41 – 7.33 (m, 2H), 7.30 (t, *J* = 7.6 Hz, 1H), 6.89 (dd, *J* = 7.9, 1.6 Hz, 1H), 3.98 (s, 3H), 3.38 (s, 3H), 1.85 (s, 3H).

$^{13}$C NMR (126 MHz, TFA-*d*) δ 170.1, 167.8, 167.2, 158.6, 149.2, 148.4, 139.3 (d, *J* = 13.9 Hz), 134.0, 133.5 (d, *J* = 11.7 Hz), 132.7, 130.5, 130.2, 126.2, 125.7, 122.0, 119.7 (d, *J* = 25.2 Hz), 105.2 (d, *J* = 27.4 Hz), 30.7, 28.9, 17.7.

$^{19}$F NMR (471 MHz, TFA-*d*) δ -77.96.

HR-MS (APCI+) calculated for $C_{20}H_{16}FN_3O_2$ [M+H$^+$]: 350.1305;**observed**: 350.1306.

*1,3-Dimethyl-5-phenylpyrimido[4,5-b]quinoline-2,4(1H,3H)-dione* ***(2x)***

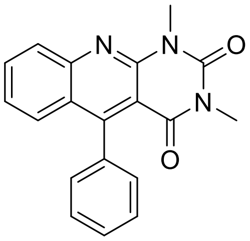

Prepared according to a general procedure from aniline (**3h**, 0.28 g, 3 mmol), benzaldehyde (**4a**, 0.32 g, 3 mmol), and *N,N*-dimethylbarbituric acid (**5**, 0.47 g, 3 mmol) in dry DMF with catalytic amount of $AlCl_3$ (reaction time 15 hours). The product was a pale yellow crystalline powder with a yield of 15% (0.14 g), m.p.: 265 – 267 °C (from 2-propanol).

$^1$H NMR (400 MHz, DMSO-$d_6$) δ 8.03 – 7.96 (m, 1H), 7.85 (ddd, *J* = 8.4, 6.8, 1.5 Hz, 1H), 7.56 – 7.49 (m, 3H), 7.48 (m, 1H), 7.28 – 7.26 (m, 1H), 7.25 – 7.21 (m, 2H), 3.73 (s, 3H), 3.18 (s, 3H).

$^1$H NMR (500 MHz, TFA-*d*) δ 8.30 – 8.24 (m, 1H), 8.16 (ddd, *J* = 8.5, 6.8, 1.6 Hz, 1H), 7.74 (ddd, *J* = 8.6, 6.9, 1.0 Hz, 1H), 7.69 (ddd, *J* = 8.6, 1.6, 0.6 Hz, 1H), 7.65 – 7.54 (m, 3H), 7.28 – 7.19 (m, 2H), 4.06 (s, 3H), 3.45 (s, 3H).

$^{13}$C NMR (126 MHz, TFA-*d*) δ 167.4, 158.9, 149.5, 147.5, 139.0, 137.2, 133.1, 130.5, 130.0, 129.4, 128.7, 126.4, 125.4, 118.8, 108.2, 30.7, 28.9

HR-MS (APCI+) calculated for $C_{19}H_{15}N_3O_2$ [M+H$^+$]: 318.1242; **observed**: 318.1242.

Spectra data were in agreement with previously reported values [2]

*7,8-Dimethoxy-1,3-dimethylpyrimido[4,5-b]quinoline-2,4(1H,3H)-dione* ***(6)***

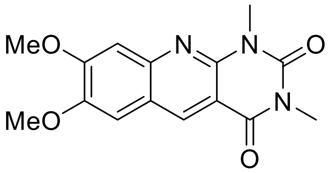

Prepared from aniline (**3b**, 0.15 g, 1 mmol) and *N,N*-dimethylbarbituric acid (**5**, 0.15 g, 1 mmol) and DMSO (3 mL, reaction time 15 hours). The product was a white crystalline powder with a yield of 15% (0.05 g), m.p.: 265 – 267 °C (from 2-propanol).

$^1$H NMR (400 MHz, *TFA-d*) δ 9.45 (s, 1H, 5-CH), 7.60 (s, 1H), 7.54 (s, 1H), 4.09 (s, 3H), 4.05 (s, 3H), 3.92 (s, 3H), 3.57 (s, 3H).

$^1$H NMR (400 MHz, DMSO-$d_6$) δ 8.87 (s, 1H, 5-CH), 7.59 (s, 1H), 7.30 (s, 1H), 3.97 (s, 3H), 3.89 (s, 3H), 3.65 (s, 3H), 3.33 (s, 3H).

$^{13}$C NMR (101 MHz, TFA-*d*) δ 160.8, 160.2, 151.3, 149.9, 146.1, 145.2, 137.6, 120.2, 108.1, 107.9, 98.9, 56.6, 55.8, 30.2, 28.7.

HR-MS (APCI+) calculated for $C_{15}H_{15}N_3O_4$ [M+H$^+$]: 302.1135; **observed**: 302.1139

Spectra data were in agreement with previously reported values [3, 4]

*7,8-dimethoxy-1,3-dimethylpyrimido[4,5-b]quinoline-2,4(1H,3H)-dione-5-d* ***(6-d)***

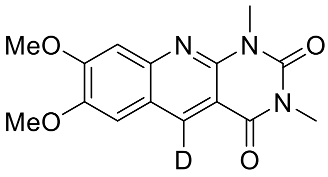

Prepared from aniline (**3b**, 0.15 g, 1 mmol) and *N,N*-dimethylbarbituric acid (**5**, 0.15 g, 1 mmol) and DMSO-$d_6$ (3 mL, reaction time 36 hours). The product was a white crystalline powder with a yield of 26% (0.08 g), m.p.: 265 – 267 °C (from 2-propanol).

$^1$H NMR (400 MHz, *TFA-d*) δ 7.62 (s, 1H), 7.56 (s, 1H), 4.11 (s, 3H), 4.07 (s, 3H), 3.94 (s, 3H), 3.56(s, 3H).

$^{1}H$ NMR (400 MHz, DMSO-$d_6$) δ 7.59 (s, 1H), 7.30 (s, 1H), 3.97 (s, 3H), 3.89 (s, 3H), 3.65 (s, 3H), 3.33 (s, 3H).

$^{13}C$ NMR (101 MHz, TFA-*d*) δ 160.8, 160.2, 151.3, 149.9, 146.3 (traces of C(5)-H), 146.0 (t, $J_{CD}$ = 25 Hz), 145.2, 137.6, 120.2, 108.1, 107.9, 98.9, 56.6, 55.8, 30.2, 28.7.

HR-MS (APCI+) calculated for $C_{15}H_{14}DN_3O_4$ [M+H$^+$]: 303.1198; **observed**: 303.1199

# Extended analysis

## Reactivity of *m*-substituted anilines

The three-component reaction with anilines bearing substituents in *meta*-position (for instance, **3d**) theoretically may lead to the formation of two regioisomers. We have observed only traces of another possible regioisomer **2m’** in the $^{1}H$ NMR spectrum of the filtrate left after crystallisation of 5-aryldeazalloxazine **2m**. The deazaalloxazine **2m’** was not isolated.

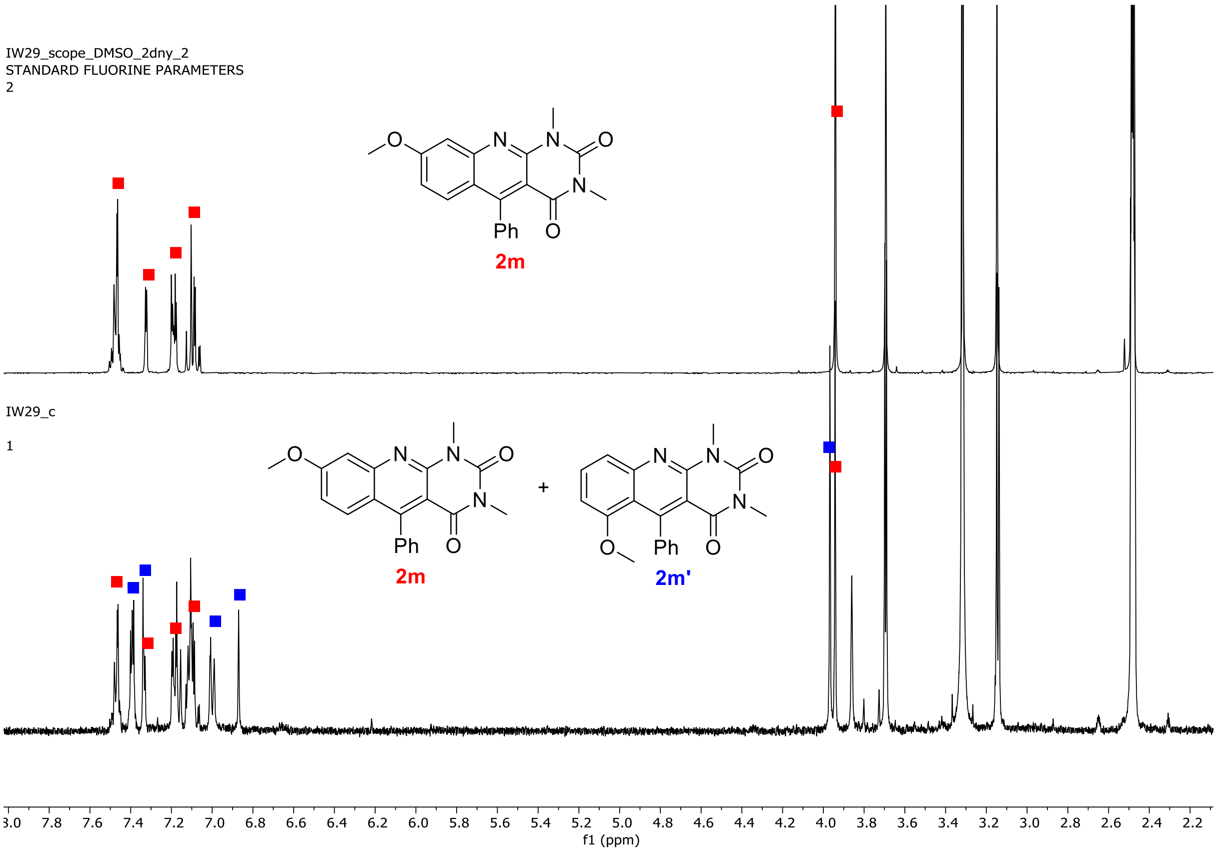


**Figure S1**. Comparison of $^{1}H$ NMR spectra of 5-aryldeazaalloxazine **2m** with the mixture of **2m** with its regioisomer **2m’**.

## Possible mechanism for the formation of deazaalloxazine 6

The deazaalloxazine **6** was formed both in the three-component reaction of aniline **3b**, mesitaldehyde (**4e**) and *N*,*N*-dimethylbarbituric acid (**5**, Scheme S1A) and in the pseudo three-component condensation of aniline **3b** and *N*,*N*-dimethylbarbituric acid (**5**) in DMSO (Scheme S1B).

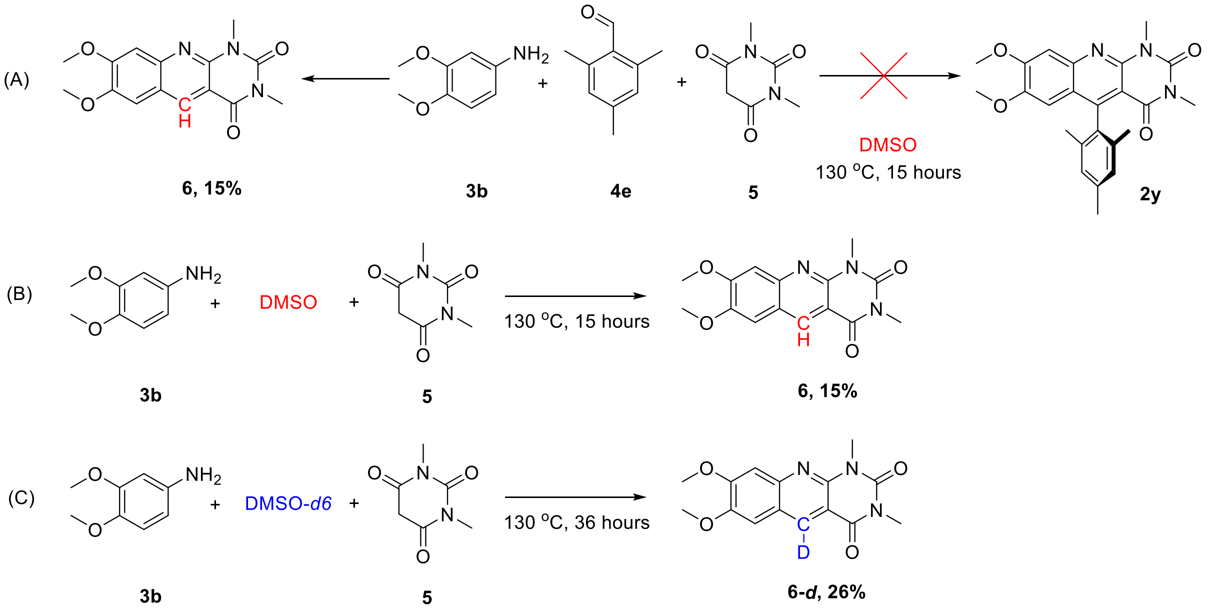


**Scheme S1:** Control experiments related to bulky substituted aldehydes.

When performing reaction in DMSO-$d_6$ (Scheme S1C), the deuterated in C(5) deazaalloxazine **6-*d*** was isolated, which indicates that DMSO was involved in the reaction providing the desired methine group. The comparison of $^1$H NMR spectra of **6** and **6-d** supports this together with mass spectrometry data (Figure S2).

(A)

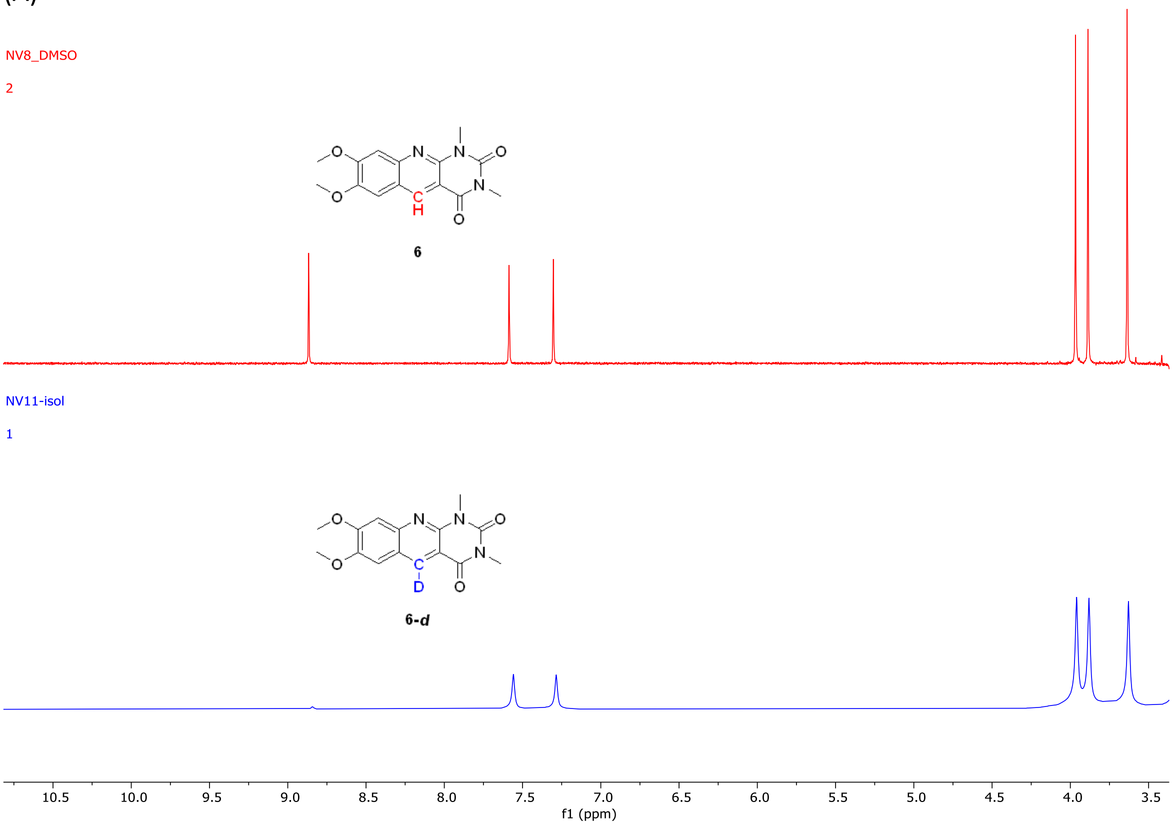
NV8_DMSO
2
6
NV11-isol
1
6-*d*
10.5
10.0
9.5
9.0
8.5
8.0
7.5
7.0
6.5
6.0
5.5
5.0
4.5
4.0
3.5
f1 (ppm)

(B)

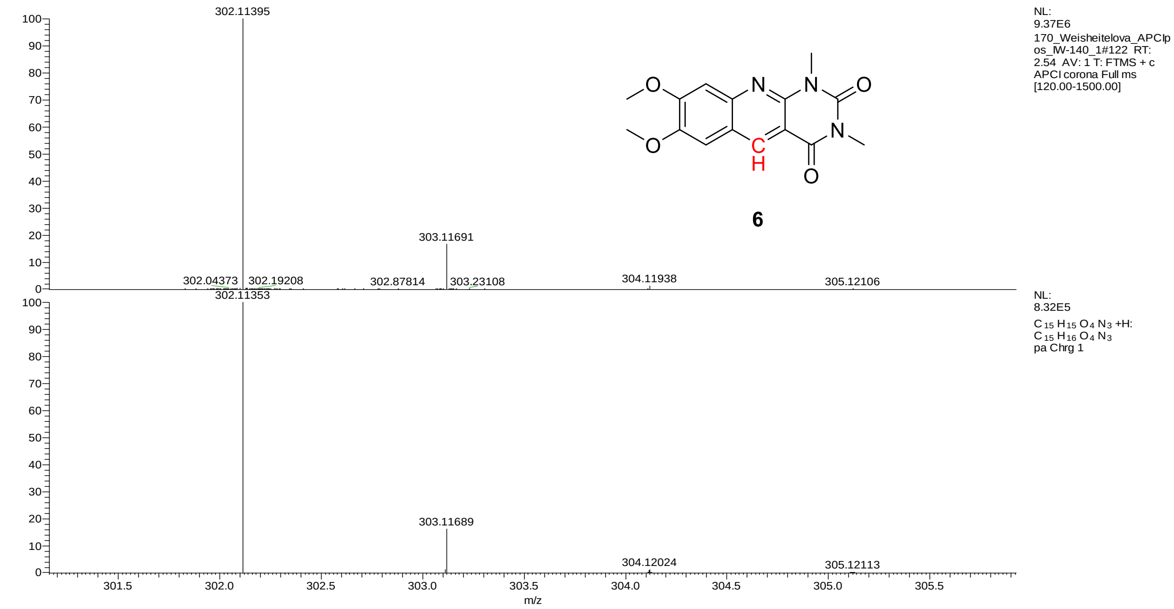
302.11395
303.11691
302.04373
302.19208
302.87814
303.23108
304.11938
305.12106
NL:
9.37E6
170_Weisheitelova_APCIpos_IW-140_1#122 RT: 2.54 AV: 1 T: FTMS + c APCI corona Full ms [120.00-1500.00]
6
302.11353
303.11689
304.12024
305.12113
NL:
8.32E5
C 15 H 15 O 4 N 3 +H:
C 15 H 16 O 4 N 3
pa Chrg 1
301.5
302.0
302.5
303.0
303.5
304.0
304.5
305.0
305.5
m/z

(C)

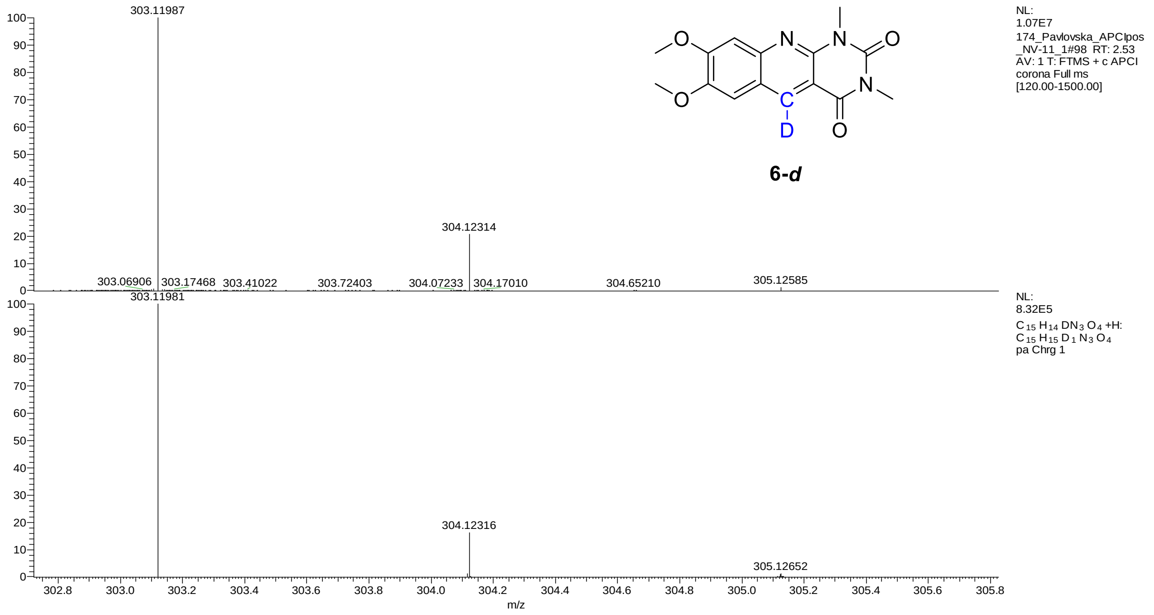


**Figure S2.** (A) Comparison of $^{1}$H NMR spectra of **6** and **6-*d***. (B) MS data for **6**. (C) MS data for **6-*d*.**

Such results together with previous reports on DMSO acting as a methine source in the synthesis of heterocyclic compounds [5, 6], suggests a following possible mechanism (Scheme S2). Thus, when reacting with *N*,*N*-dimethylbarbituric acid (**5**), aniline **3b** forms the imine (**A**), which may isomerize to a more stable anilinouracil intermediate (**B**). DMSO is activated with the generation of the electrophilic intermediate (**C**), which provides the azadiene intermediate (**D**). The last forms 5-deazaalloxazine **6** after a possible annulation reaction. When aromatic aldehyde in the three-component reaction is involved, it plays the role of electrophilic fragment when reacting with anilinouracil **C**. In this case, we did not observe Knoevenagel adducts as intermediates. It should be noted that acidic catalysis (with TFA) might facilitate reaction by activating DMSO.

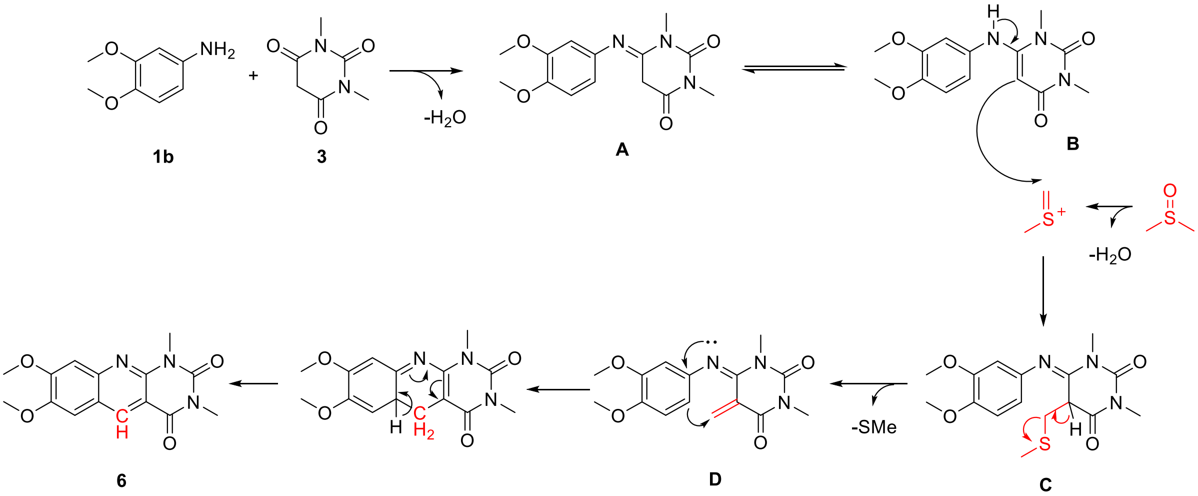


**Scheme 2:** Proposed mechanism for the formation of the 5-deazaalloxazine **6**.

# NMR spectra of isolated products

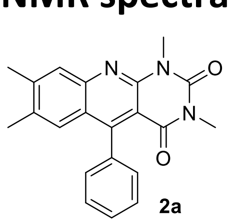

**2a**

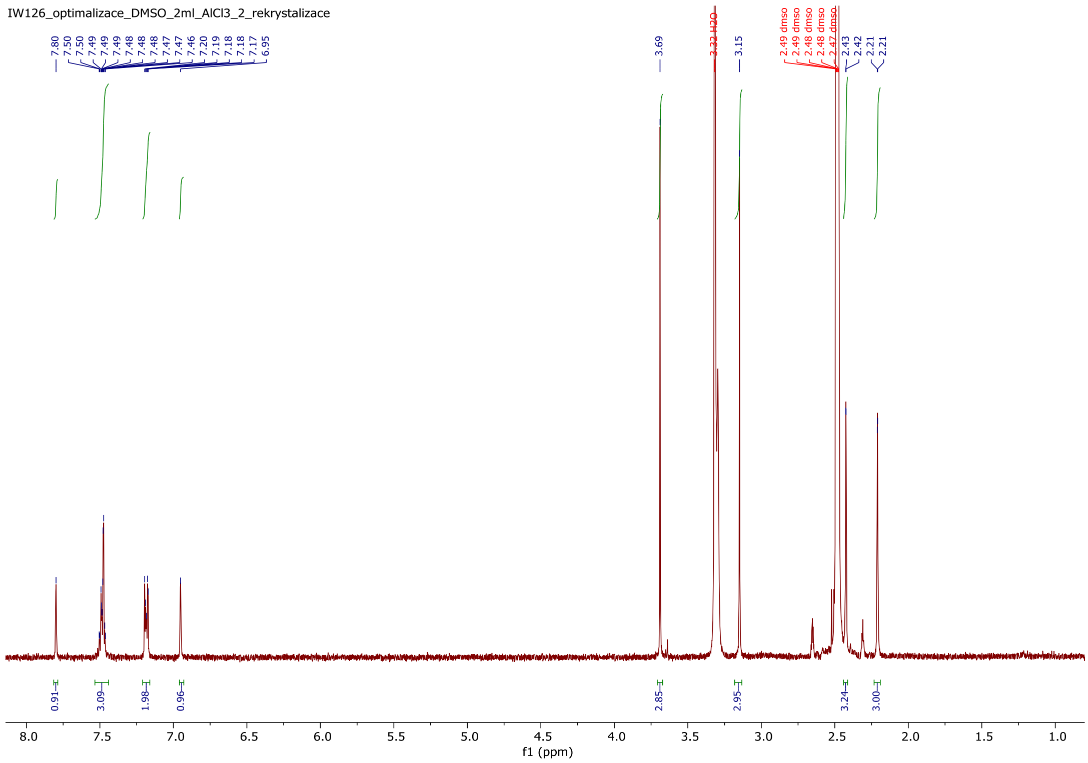


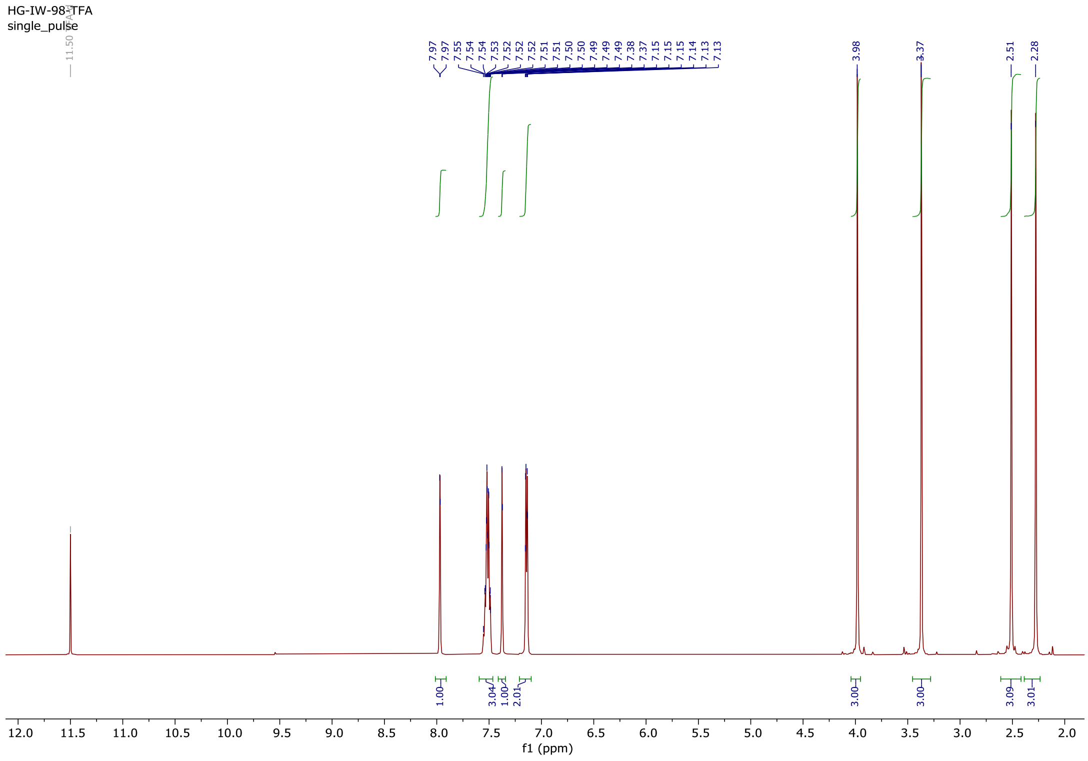

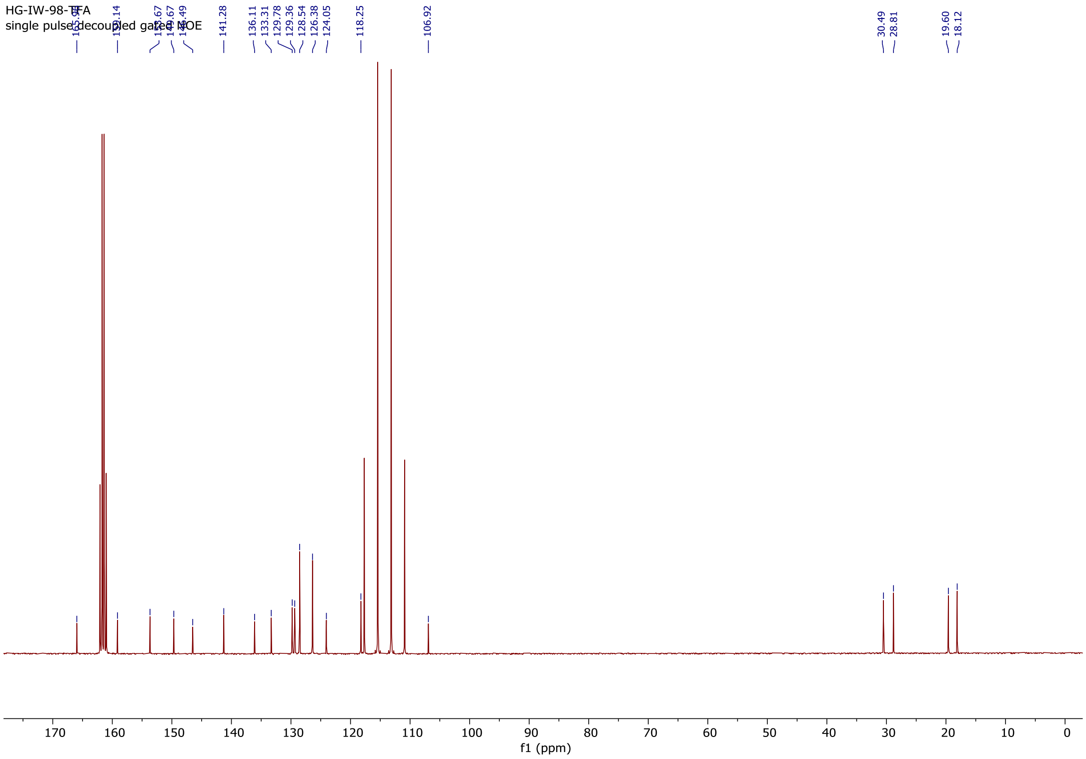


**Figure S3.** $^{1}H$ and $^{13}C$ NMR spectra of **2a**

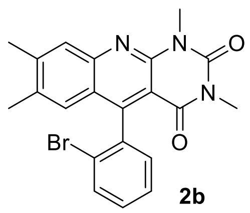

**2b**

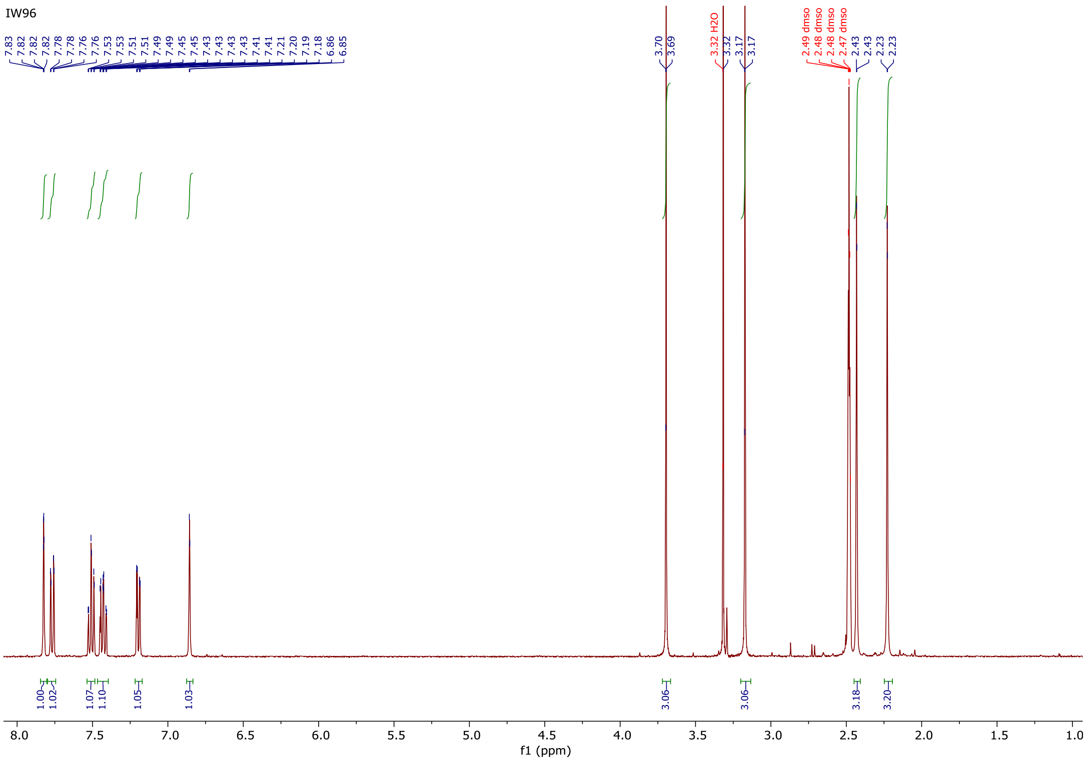

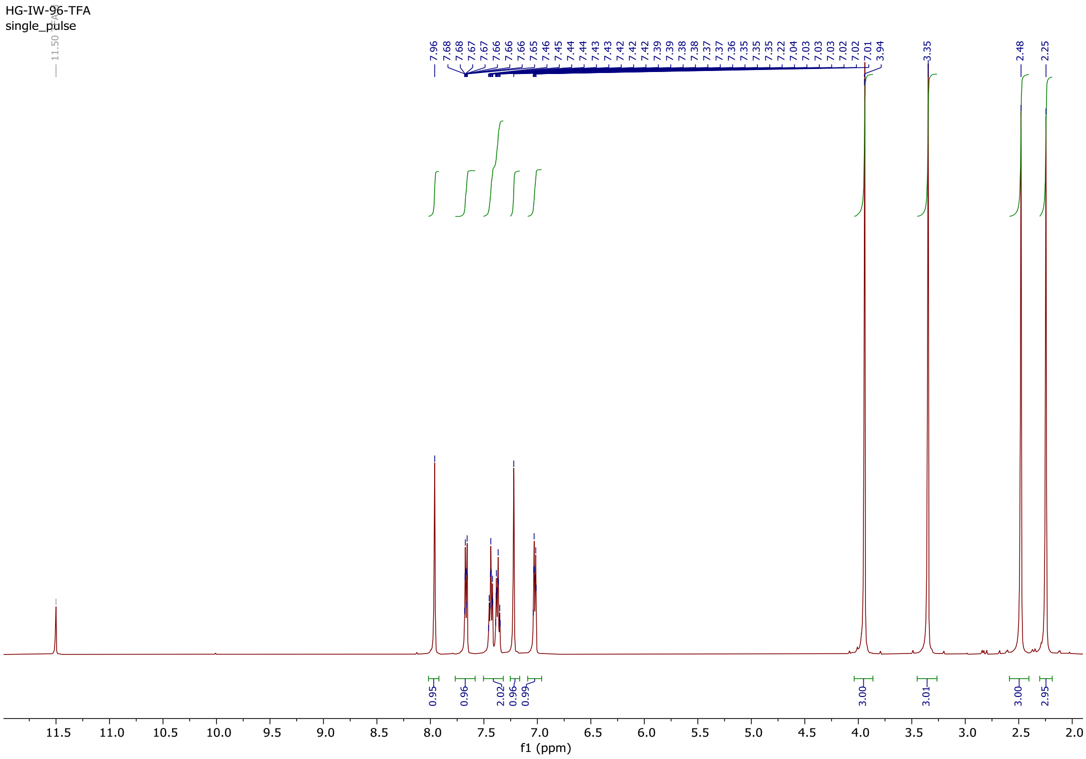

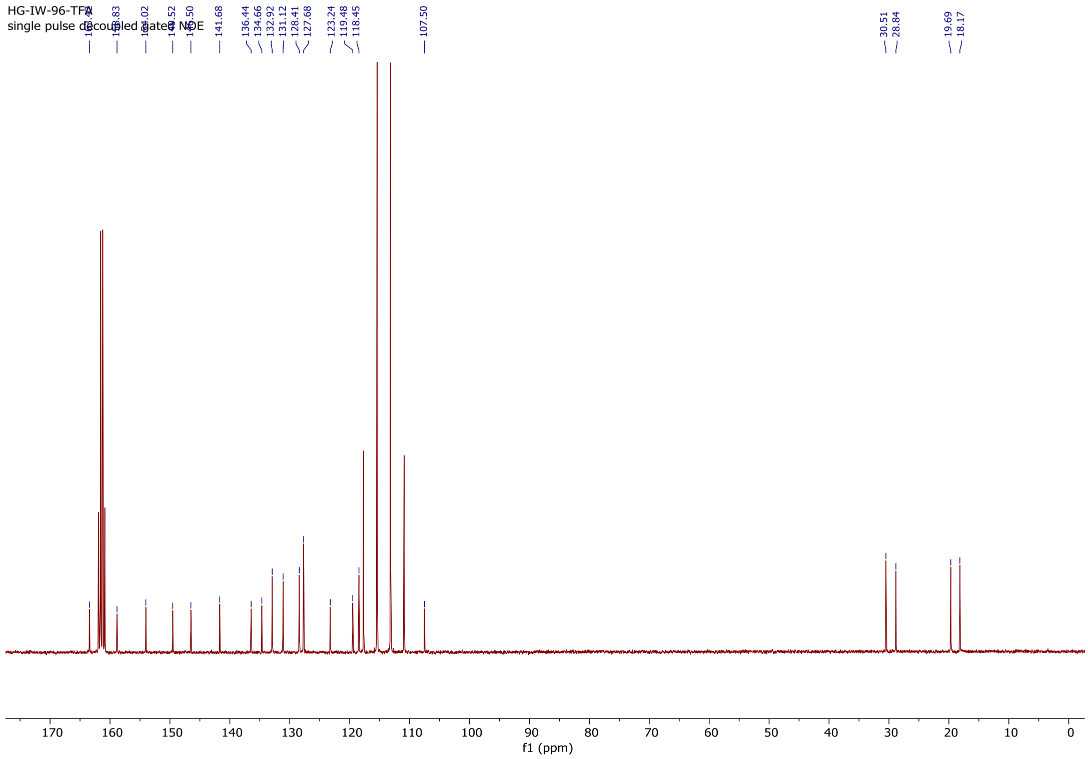


**Figure S4.** $^{1}$H and $^{13}$C NMR spectra of **2b**

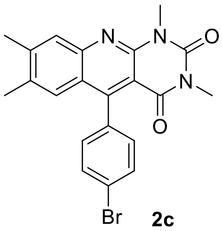

**2c**

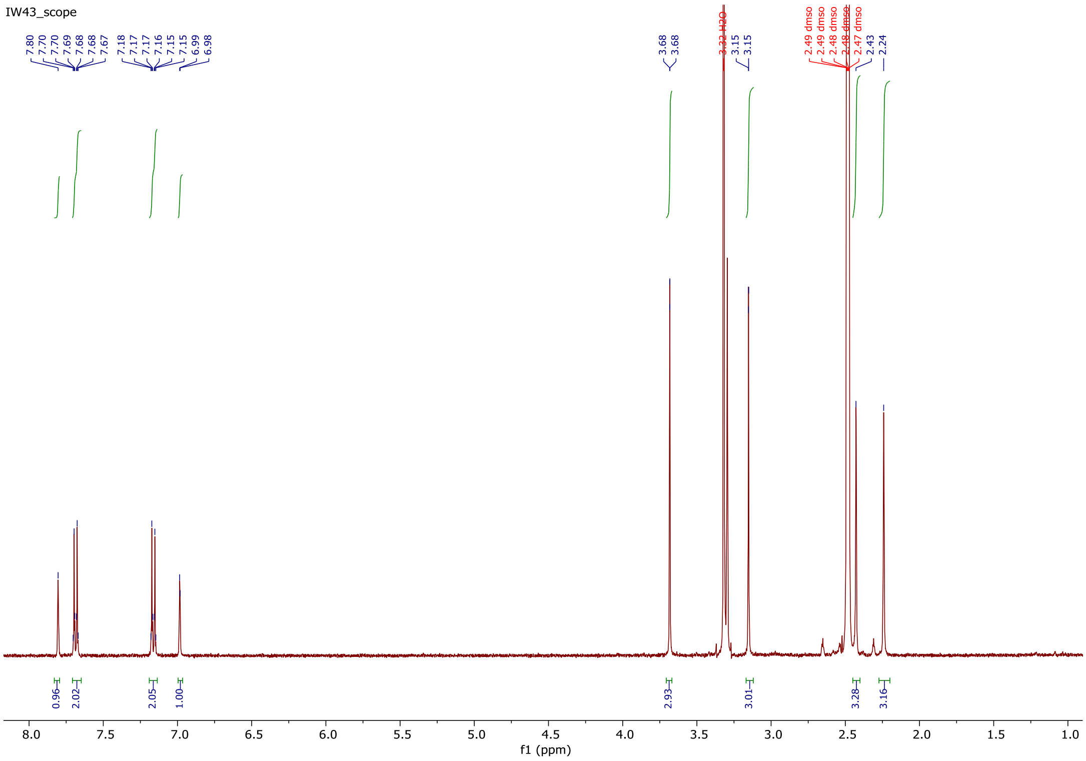
IW43_scope
7.80
7.70
7.70
7.69
7.68
7.68
7.67
7.18
7.17
7.17
7.16
7.15
7.15
6.99
6.98
3.68
3.68
3.32 H2O
3.15
3.15
2.49 dmso
2.49 dmso
2.48 dmso
2.48 dmso
2.47 dmso
2.43
2.24
0.96
2.02
2.05
1.00
2.93
3.01
3.28
3.16
f1 (ppm)

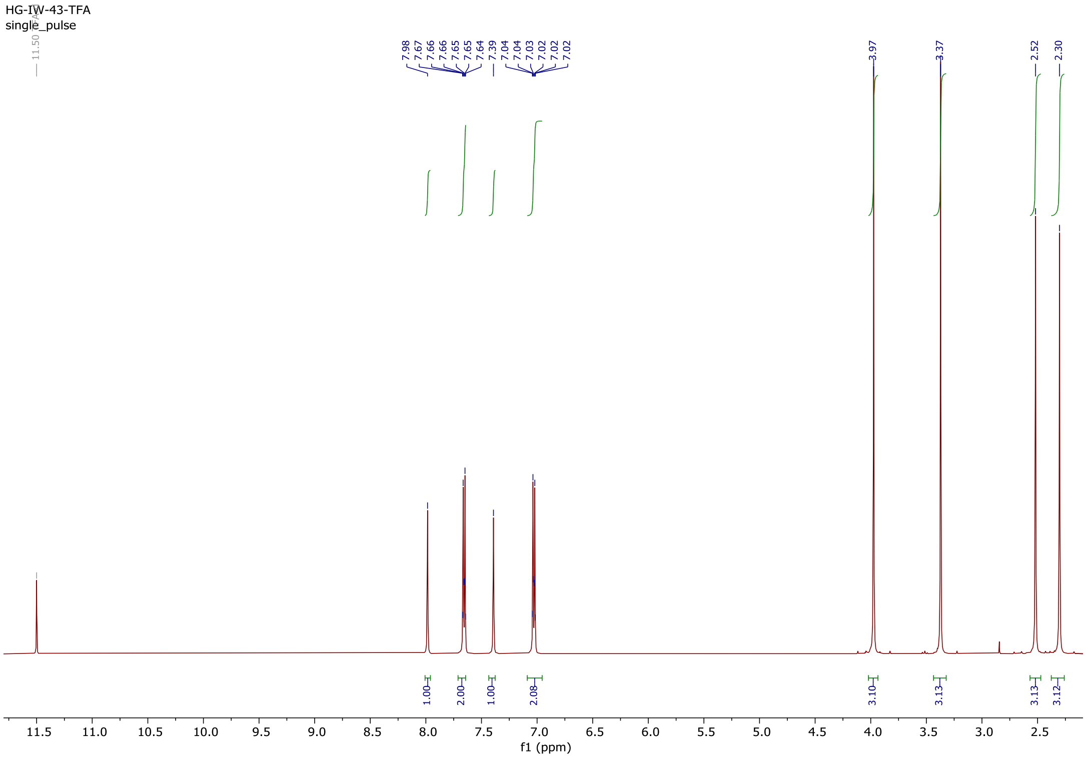
HG-IW-43-TFA
single_pulse
11.50
7.98
7.67
7.66
7.66
7.65
7.65
7.64
7.39
7.04
7.04
7.03
7.02
7.02
7.02
3.97
3.37
2.52
2.30
1.00
2.00
1.00
2.08
3.10
3.13
3.13
3.12
f1 (ppm)

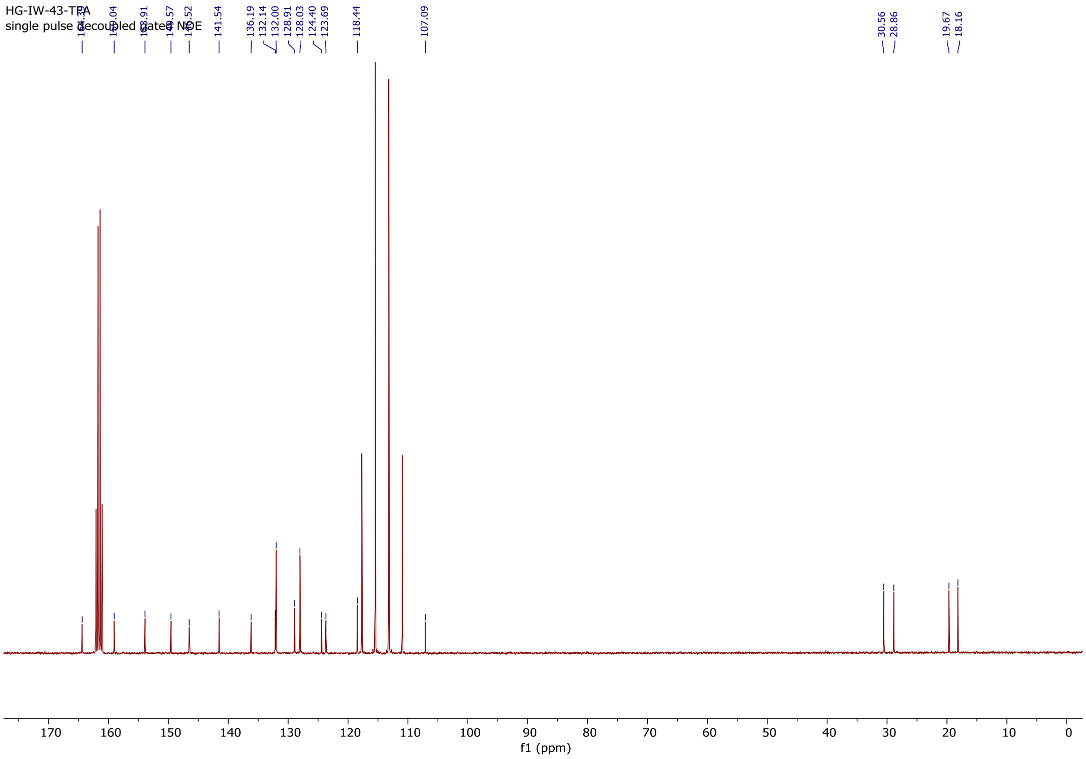


**Figure S5.** $^{1}H$ and $^{13}C$ NMR spectra of **2c**

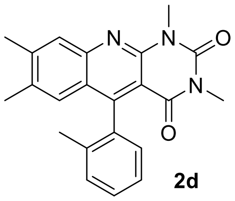

**2d**

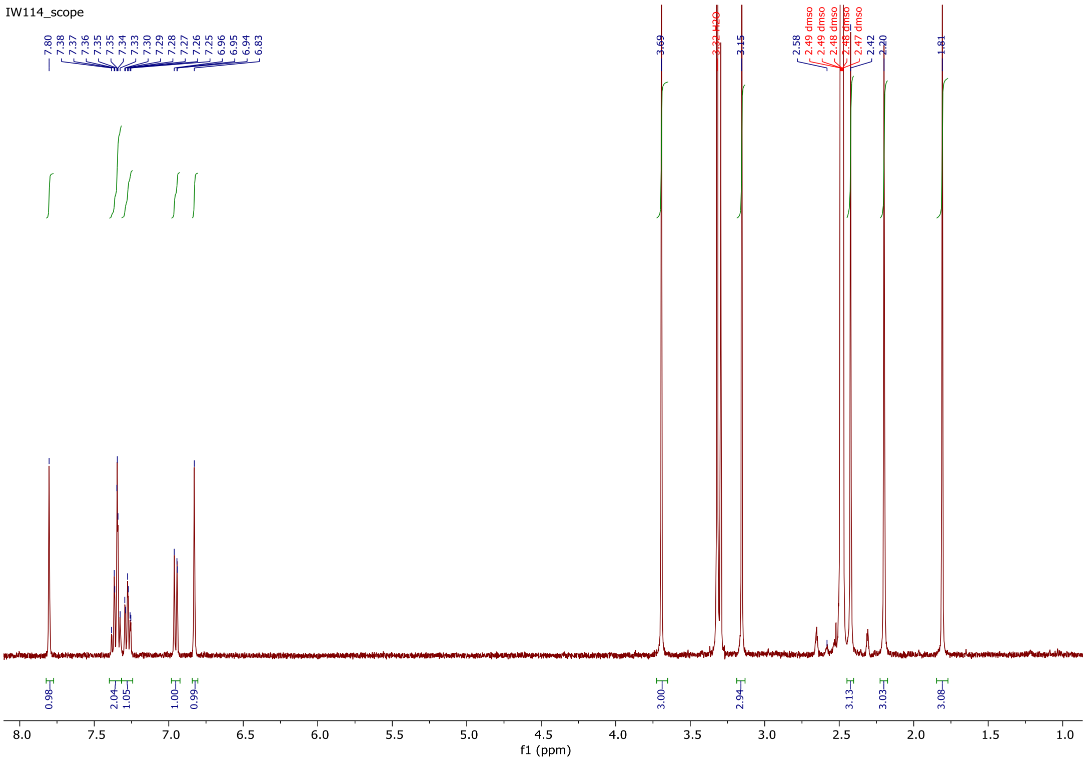


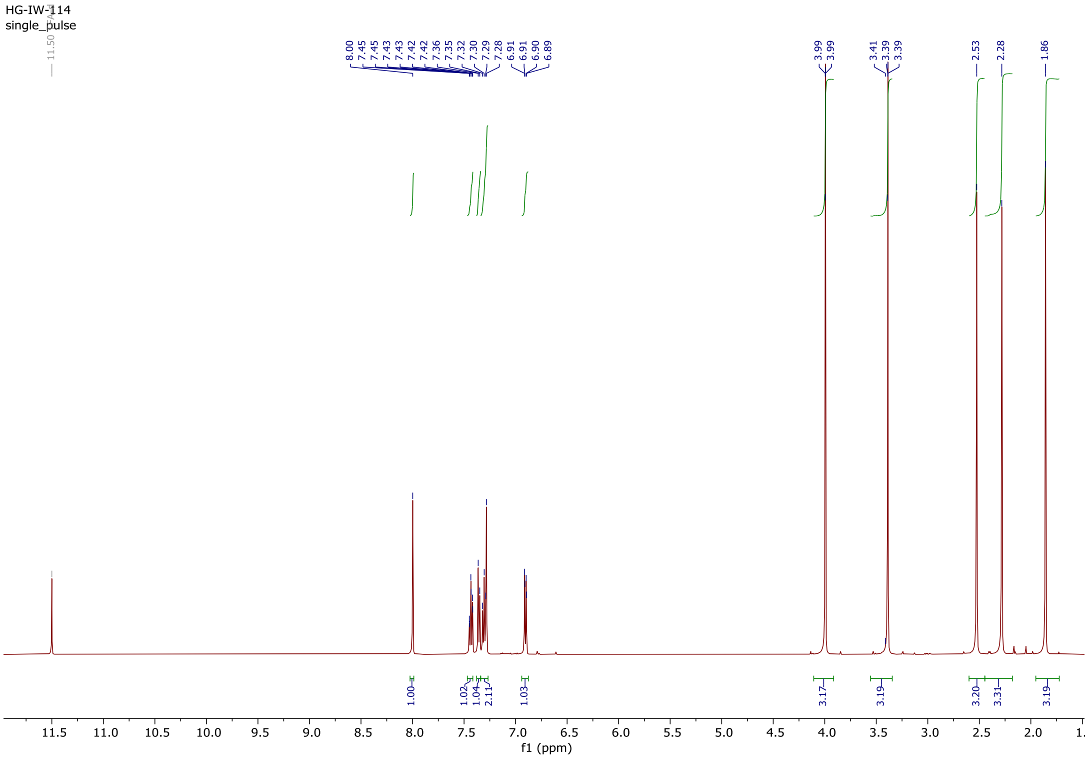

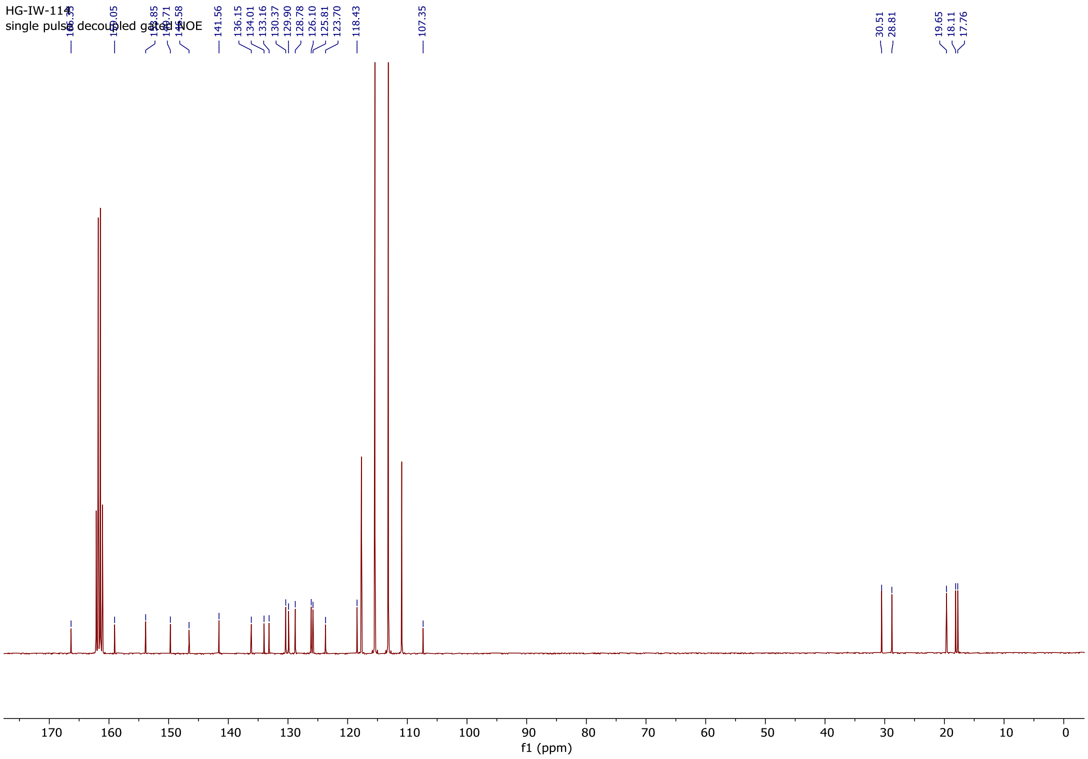


**Figure S6.** $^{1}H$ and $^{13}C$ NMR spectra of **2d**

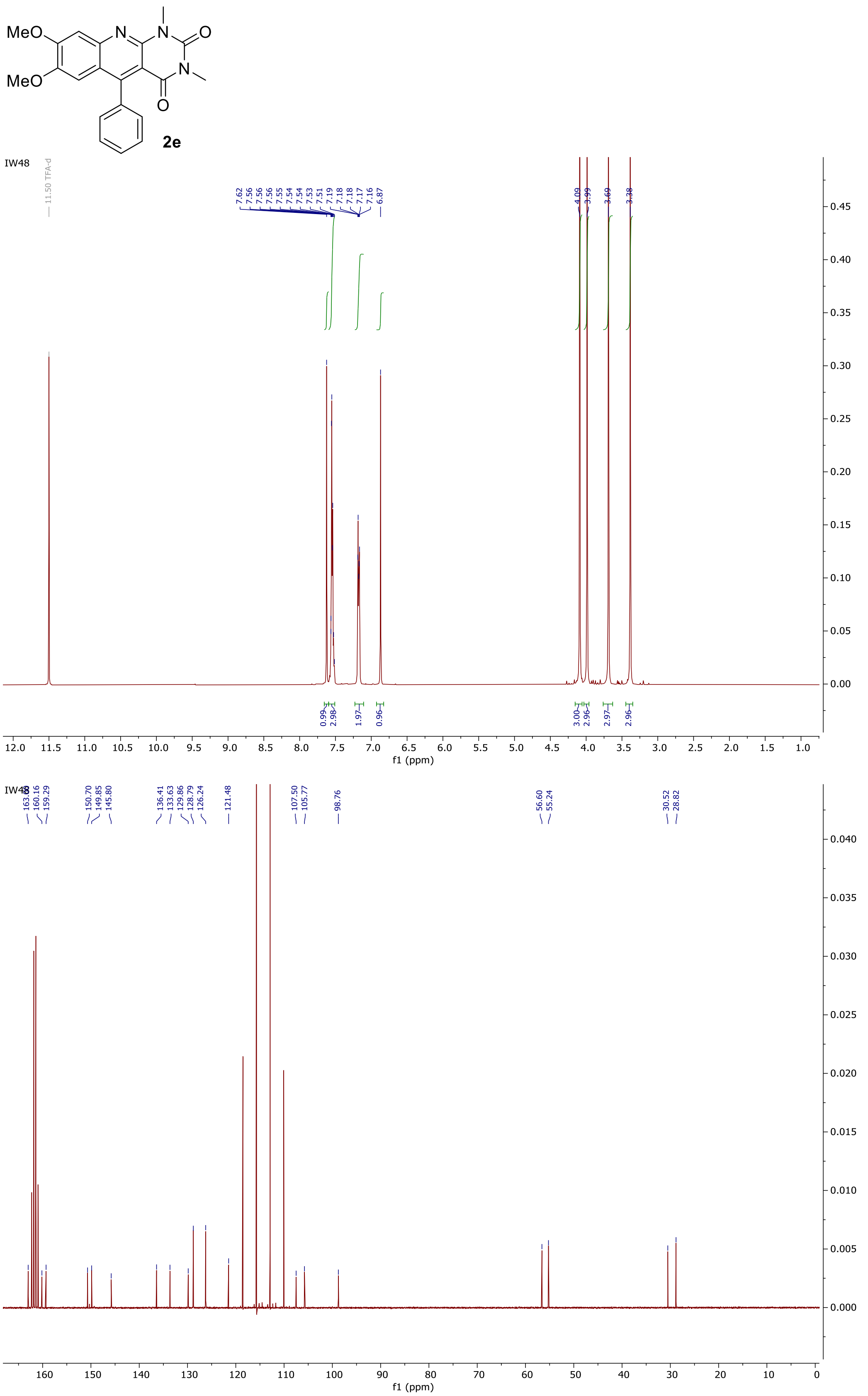


**Figure S7.** $^{1}$H and $^{13}$C NMR spectra of **2e**.

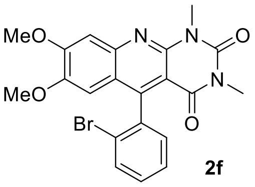



**2f**

**Figure S7.** $^{1}$H and 13C NMR spectra of **2e**

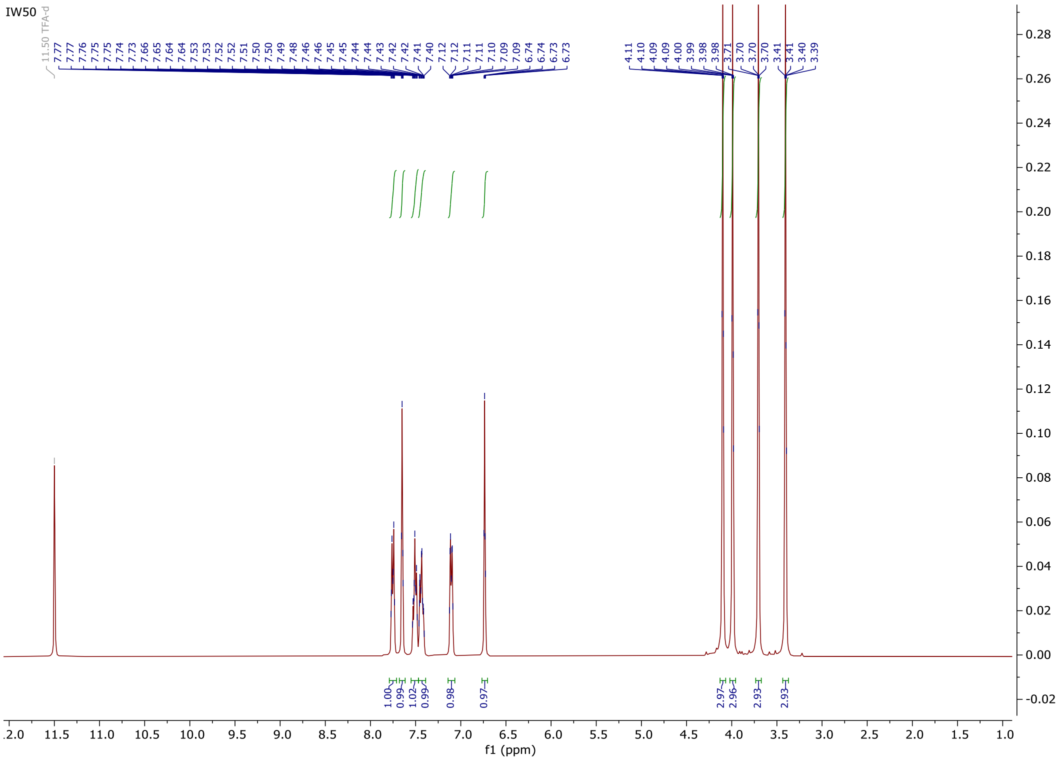



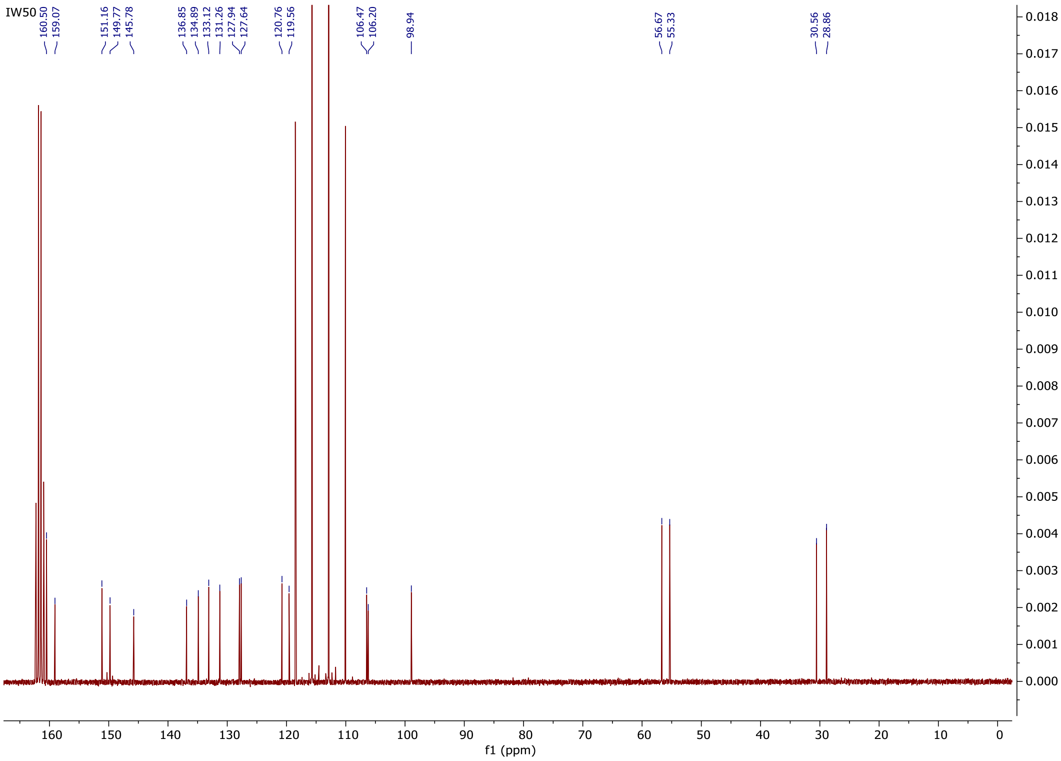



**Figure 8.** $^{1}$H and $^{13}$C NMR spectra of **2f**

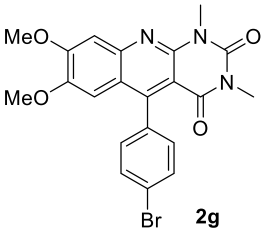
MeO
MeO
N
N
N
O
O
Br
2g

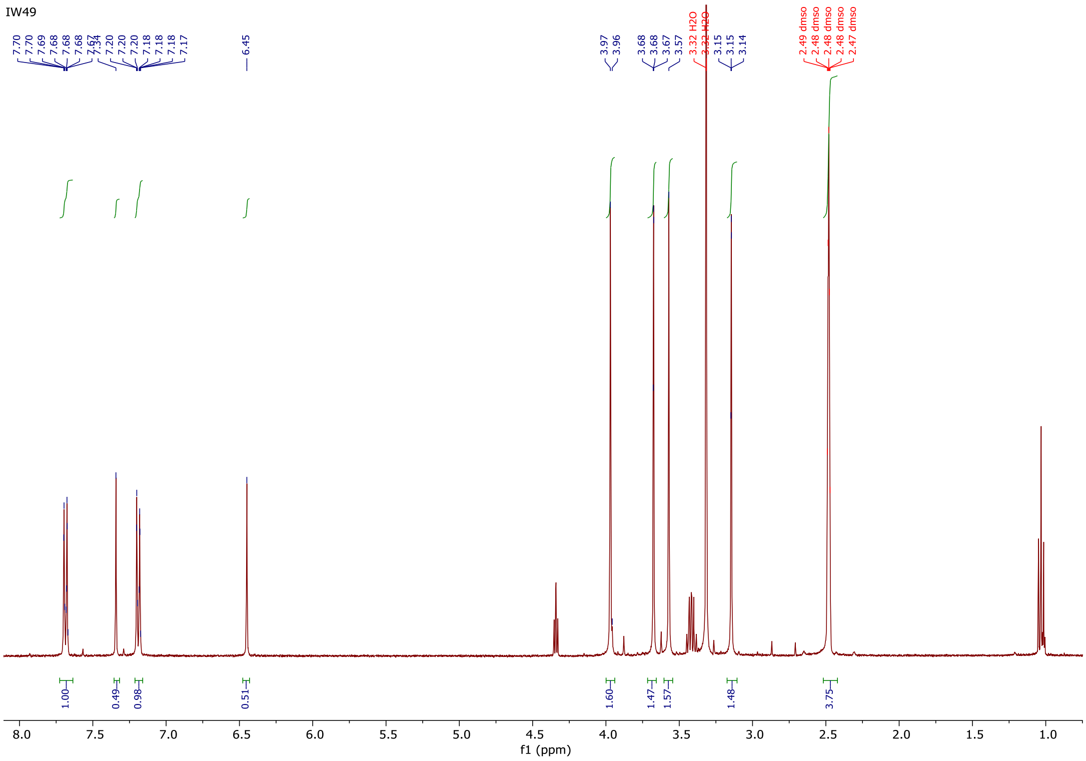
IW49
7.70
7.70
7.69
7.68
7.68
7.68
7.67
7.34
7.20
7.20
7.20
7.18
7.18
7.18
7.17
6.45
3.97
3.96
3.68
3.68
3.67
3.57
3.32 H2O
3.31 H2O
3.15
3.15
3.14
2.49 dmso
2.48 dmso
2.48 dmso
2.48 dmso
2.47 dmso
1.00
0.49
0.98
0.51
1.60
1.47
1.57
1.48
3.75
8.0
7.5
7.0
6.5
6.0
5.5
5.0
4.5
4.0
3.5
3.0
2.5
2.0
1.5
1.0
f1 (ppm)

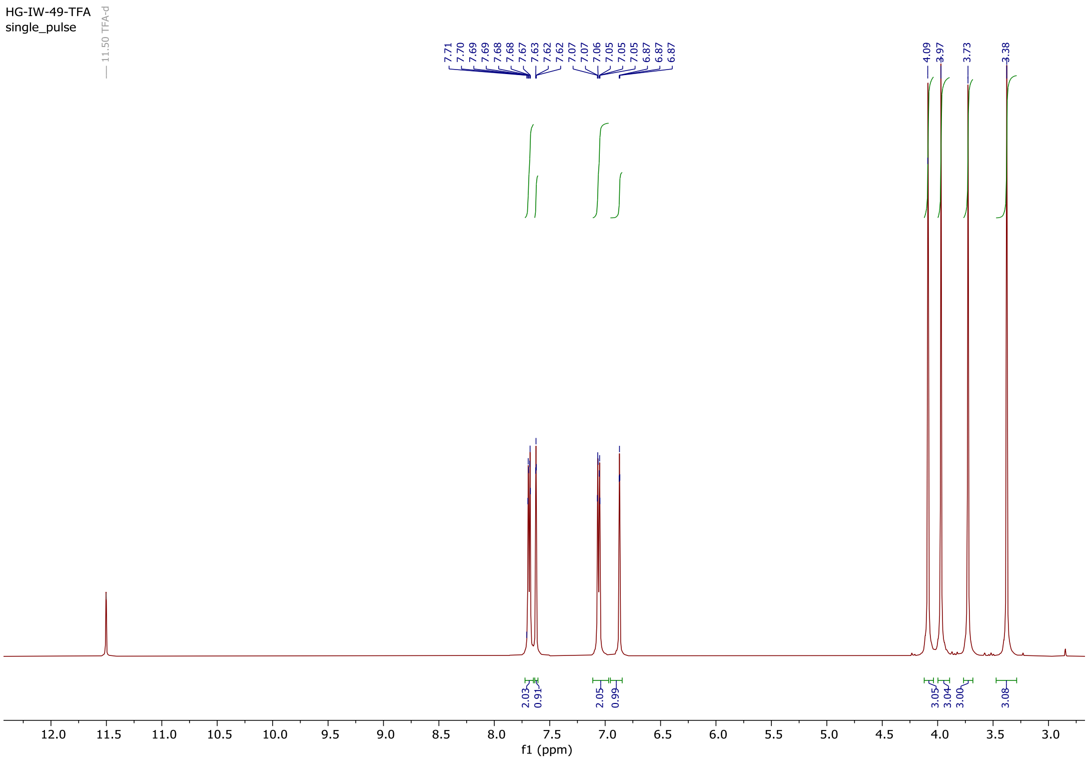
HG-IW-49-TFA
single_pulse
11.50 TFA-d
7.71
7.70
7.69
7.68
7.68
7.67
7.63
7.62
7.62
7.07
7.06
7.05
7.05
7.05
6.87
6.87
6.87
4.09
3.97
3.73
3.38
2.03
0.91
2.05
0.99
3.05
3.04
3.00
3.08
12.0
11.5
11.0
10.5
10.0
9.5
9.0
8.5
8.0
7.5
7.0
6.5
6.0
5.5
5.0
4.5
4.0
3.5
3.0
f1 (ppm)

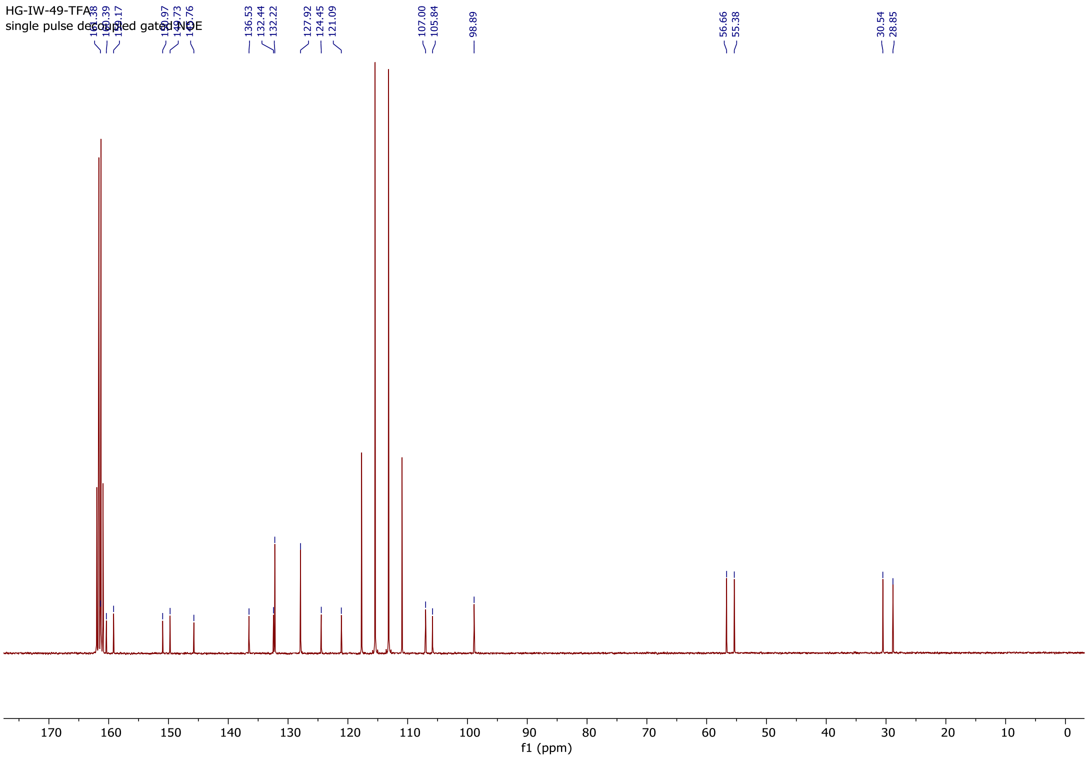


**Figure 9** $^{1}$H and $^{13}$C NMR spectra of **2g**

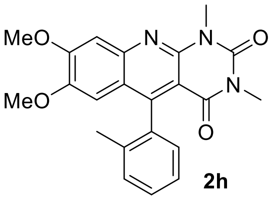
MeO
MeO
N
N
N
O
O

**2h**

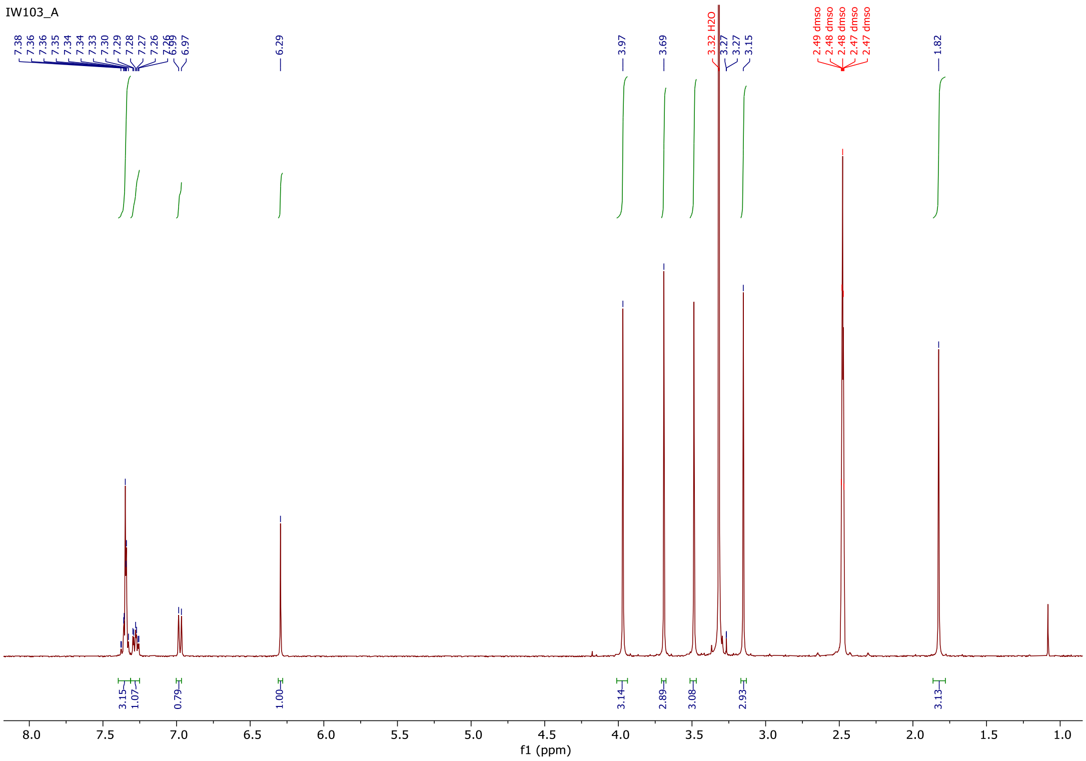
IW103_A
7.38
7.36
7.36
7.35
7.34
7.34
7.33
7.30
7.29
7.28
7.27
7.26
7.26
6.99
6.97
6.29
3.97
3.69
3.32 H2O
3.27
3.27
3.15
2.49 dmso
2.48 dmso
2.48 dmso
2.47 dmso
2.47 dmso
1.82
3.15
1.07
0.79
1.00
3.14
2.89
3.08
2.93
3.13
8.0 7.5 7.0 6.5 6.0 5.5 5.0 4.5 4.0 3.5 3.0 2.5 2.0 1.5 1.0
f1 (ppm)

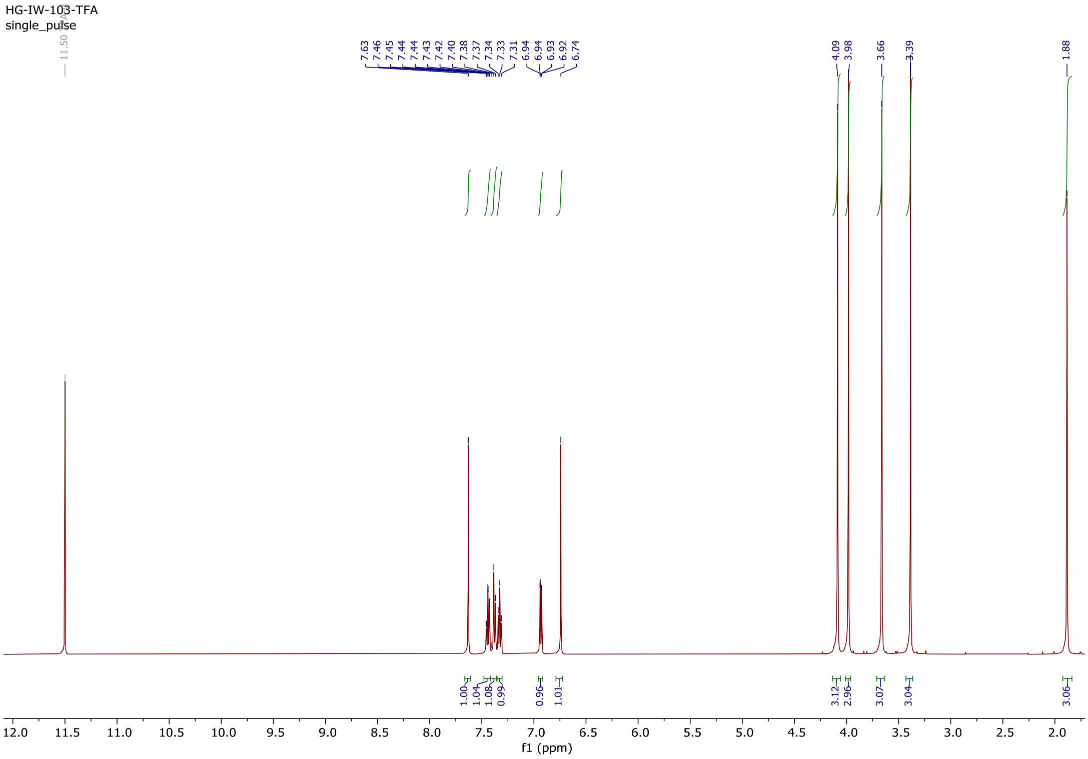
HG-IW-103-TFA
single_pulse
11.50
7.63
7.46
7.45
7.44
7.44
7.43
7.42
7.40
7.38
7.37
7.34
7.33
7.31
6.94
6.94
6.93
6.92
6.74
4.09
3.98
3.66
3.39
1.88
1.00
1.04
1.08
0.99
0.96
1.01
3.12
2.96
3.07
3.04
3.06
12.0 11.5 11.0 10.5 10.0 9.5 9.0 8.5 8.0 7.5 7.0 6.5 6.0 5.5 5.0 4.5 4.0 3.5 3.0 2.5 2.0
f1 (ppm)

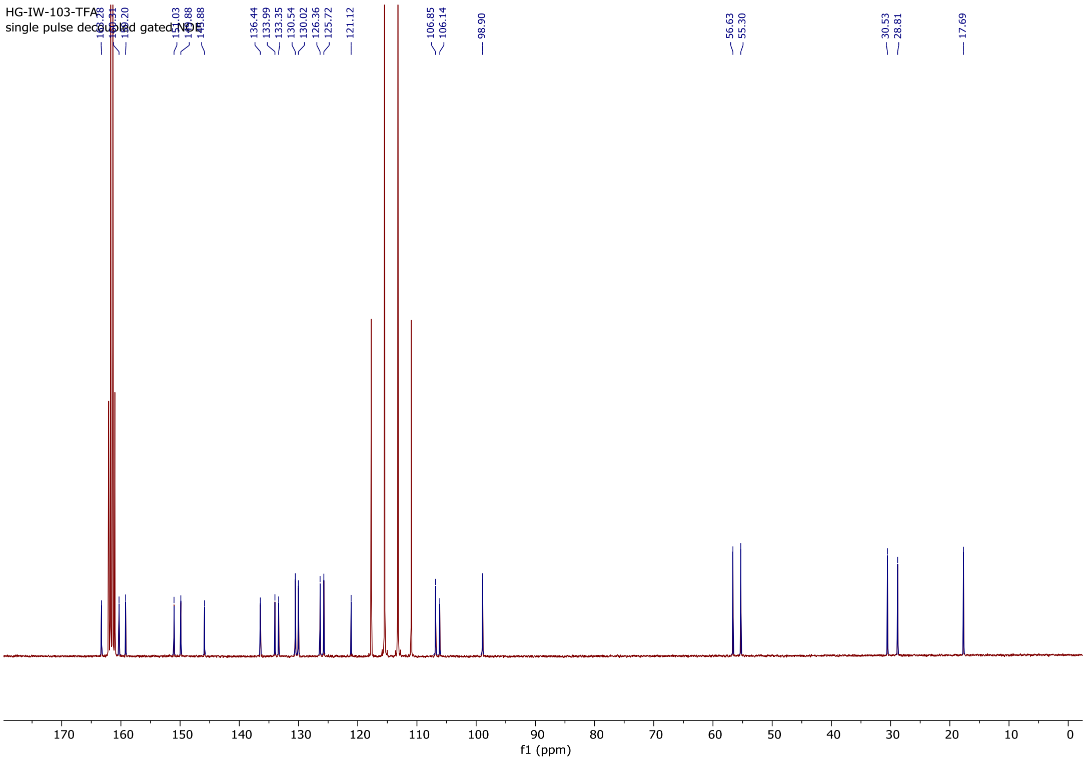


**Figure 10** $^{1}H$ and $^{13}C$ NMR spectra of **2h**

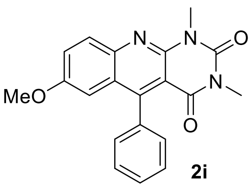


**2i**

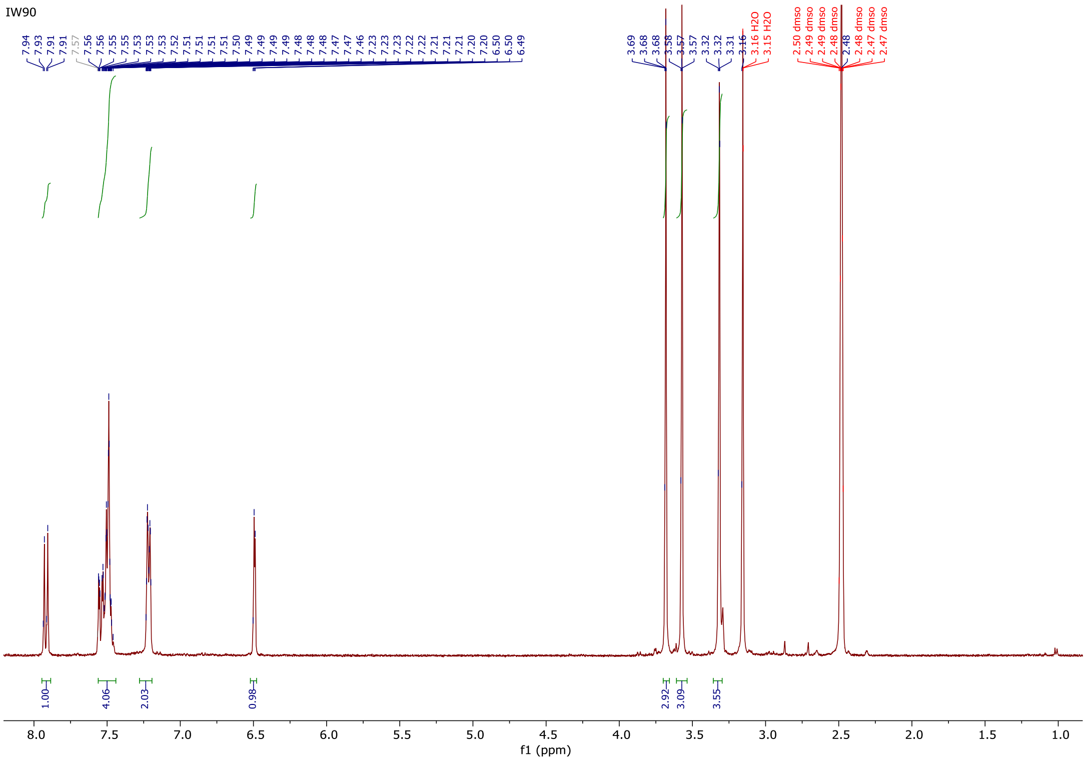


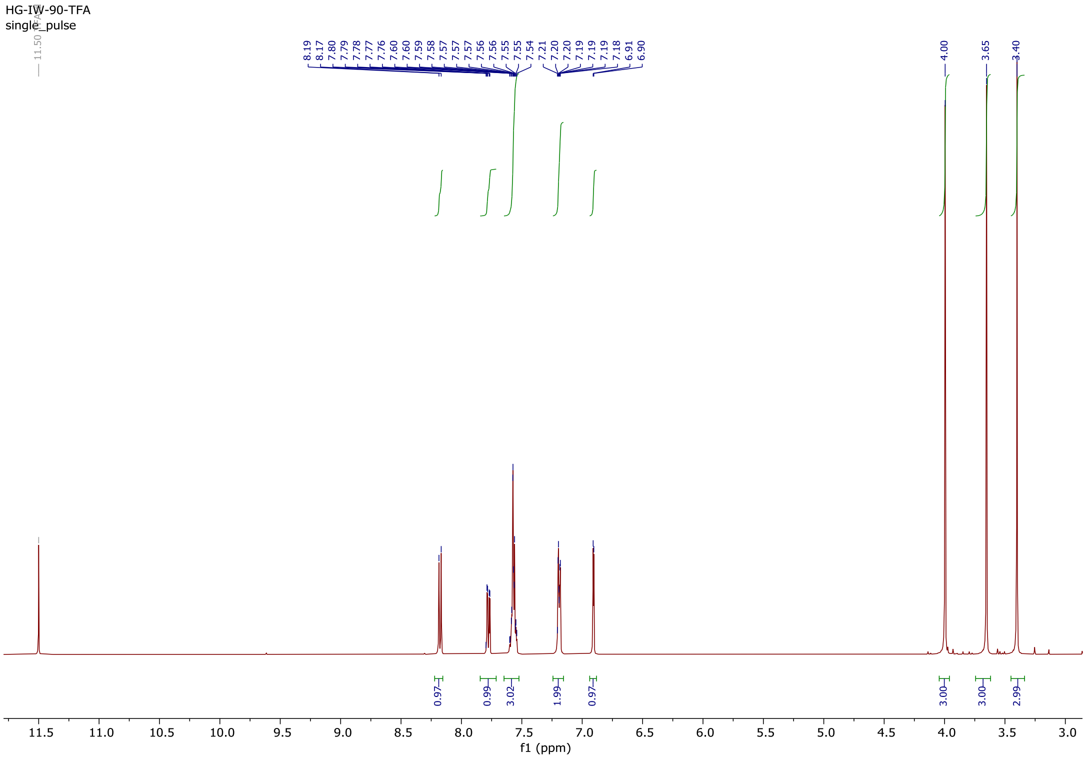

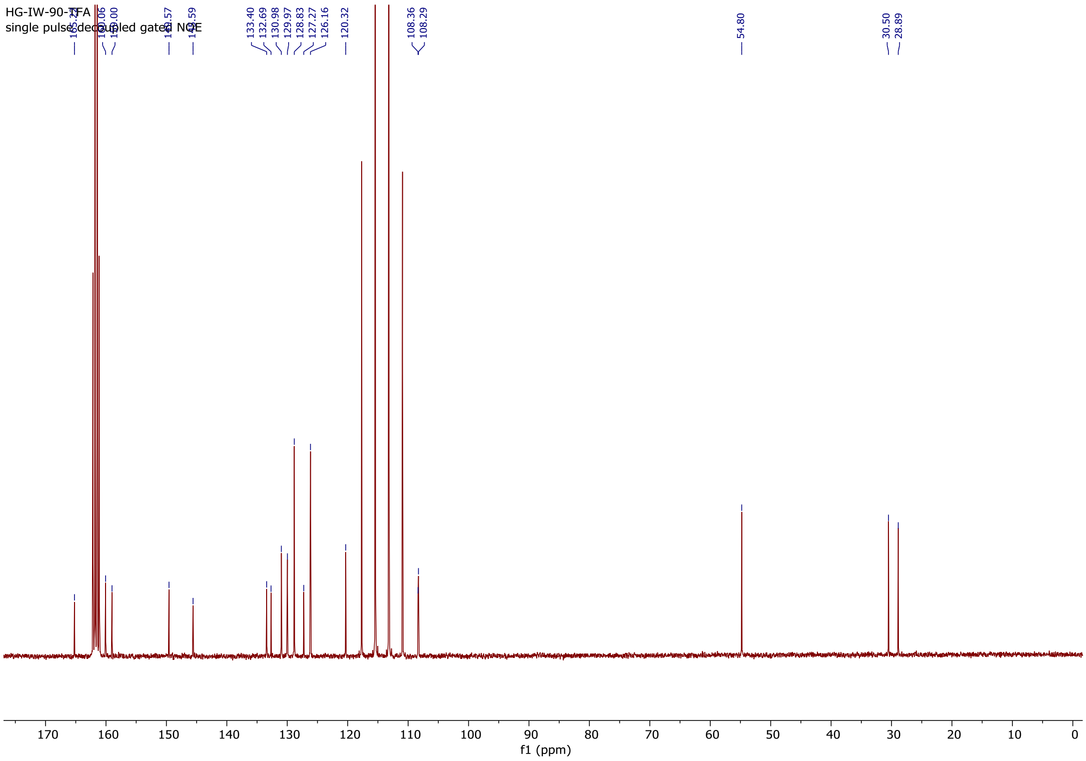


**Figure 11** $^{1}$H and $^{13}$C NMR spectra of **2i**

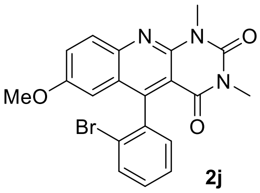
MeO
Br
N
N
O
N
O

**2j**

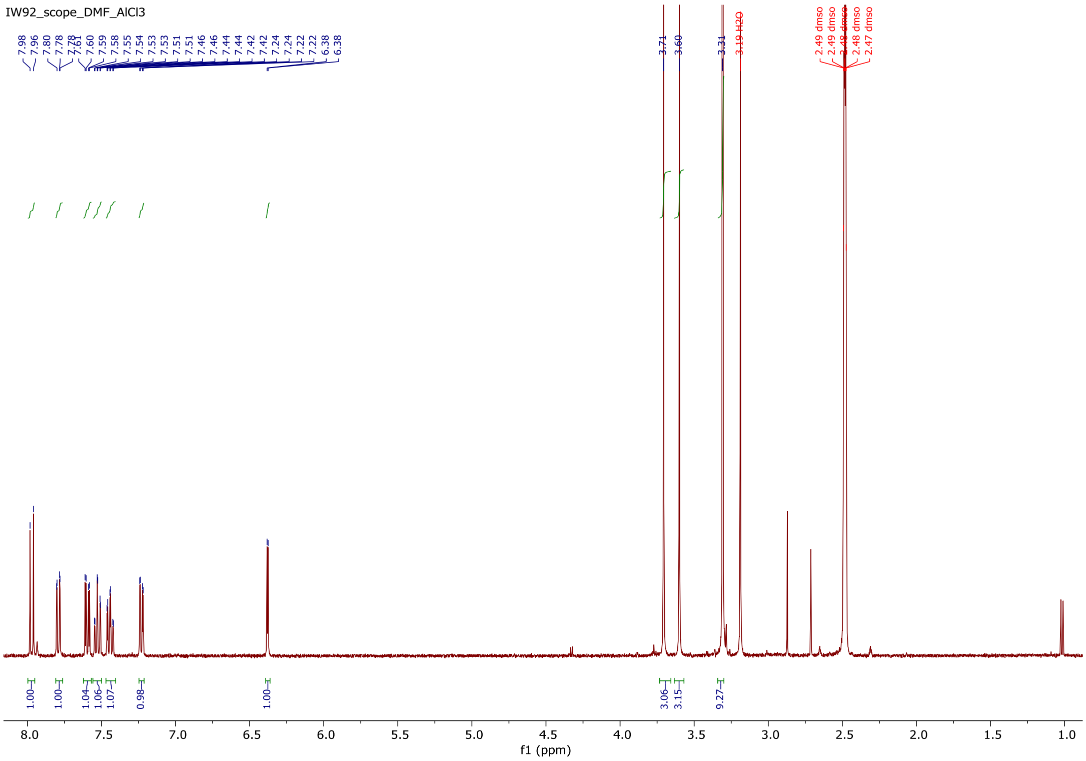
IW92_scope_DMF_AlCl3
f1 (ppm)

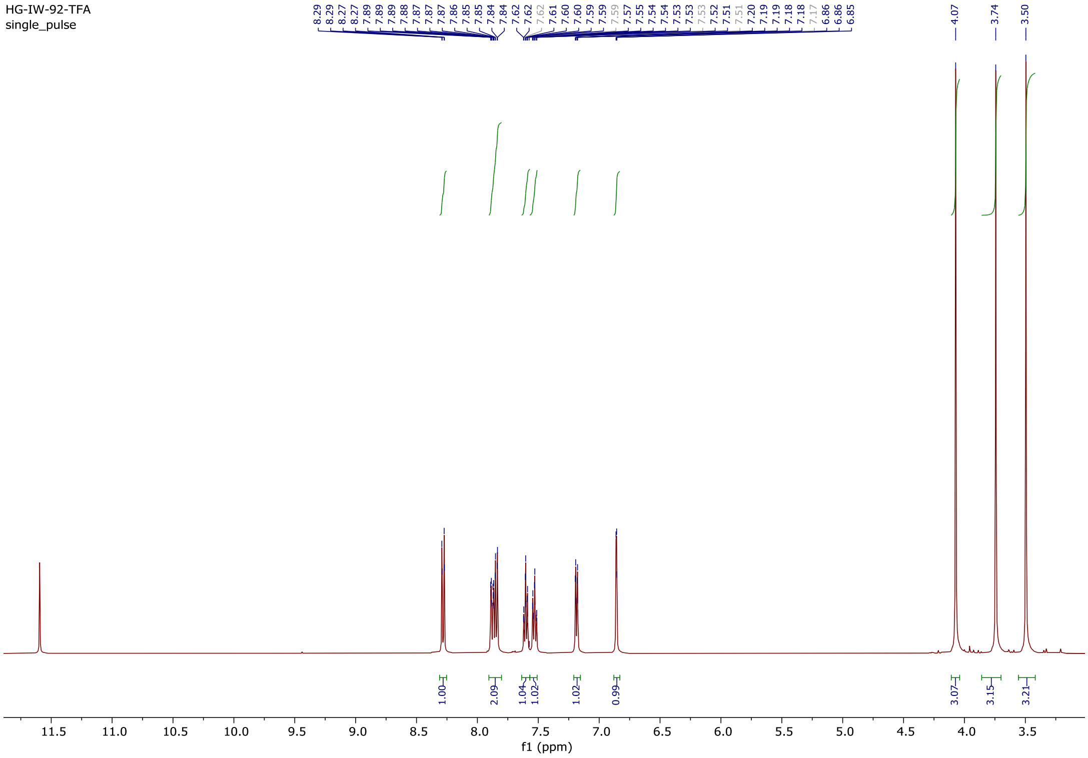
HG-IW-92-TFA
single_pulse
f1 (ppm)

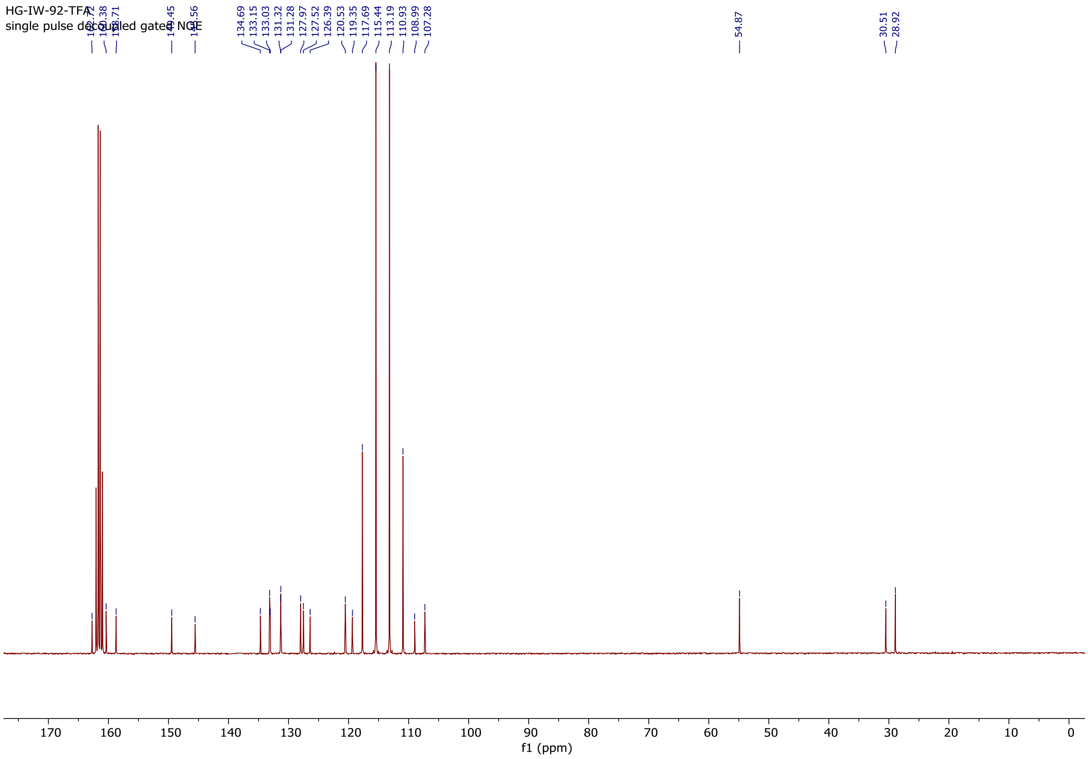


**Figure 12** $^{1}$H and $^{13}$C NMR spectra of **2j**

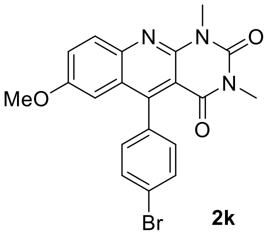
MeO
N
N
N
O
O
Br

**2k**

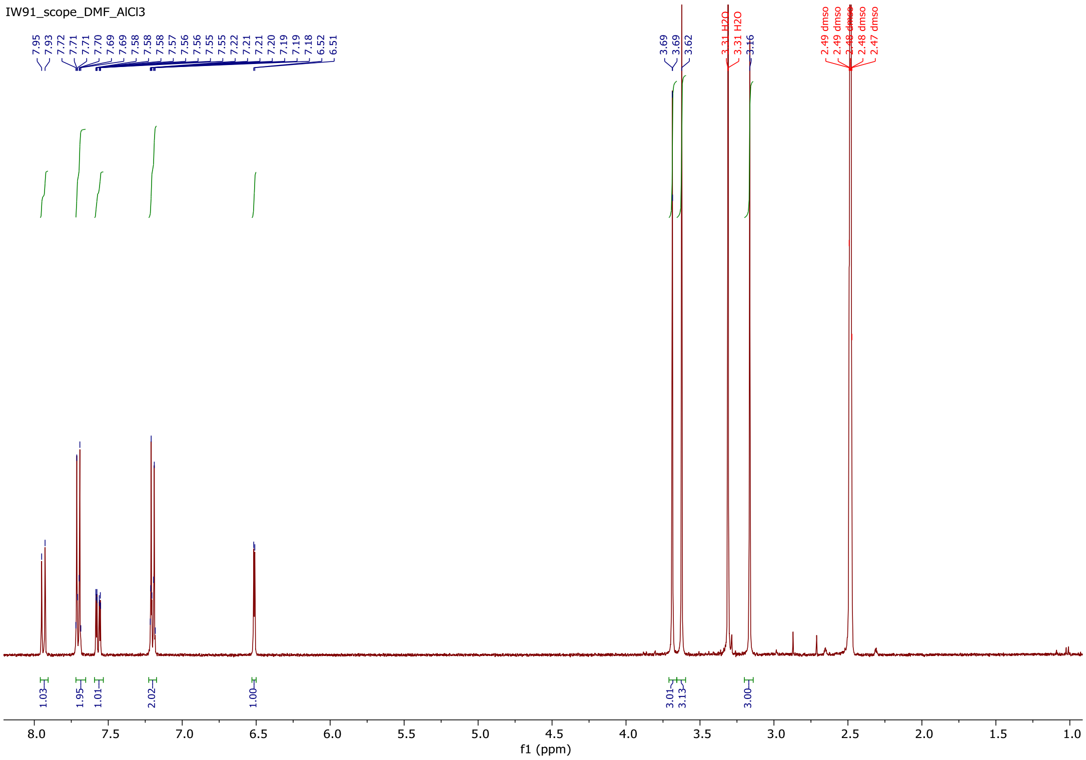
IW91_scope_DMF_AlCl3
7.95
7.93
7.72
7.71
7.70
7.69
7.69
7.58
7.58
7.57
7.56
7.55
7.55
7.22
7.21
7.21
7.20
7.19
7.18
6.52
6.51
3.69
3.69
3.62
3.31 H2O
3.31 H2O
3.16
2.49 dmso
2.49 dmso
2.48 dmso
2.48 dmso
2.47 dmso
1.03
1.95
1.01
2.02
1.00
3.01
3.13
3.00
f1 (ppm)

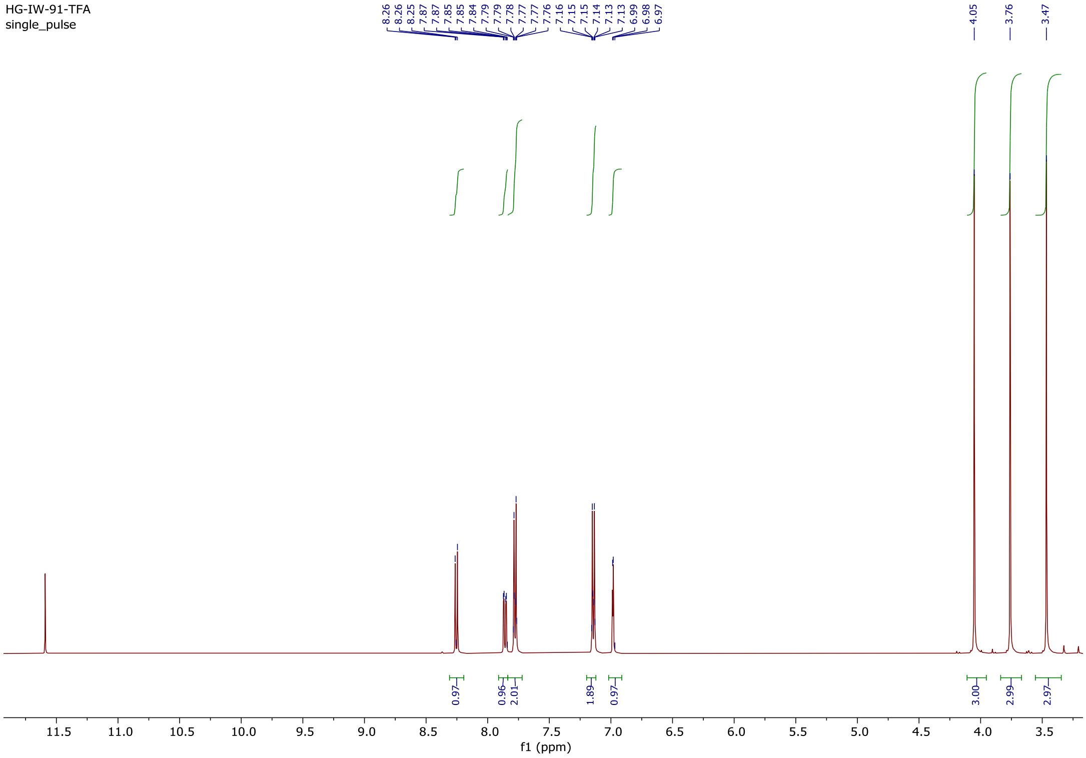
HG-IW-91-TFA
single_pulse
8.26
8.26
8.25
7.87
7.87
7.85
7.85
7.84
7.79
7.78
7.77
7.77
7.76
7.16
7.15
7.14
7.13
6.99
6.98
6.97
4.05
3.76
3.47
0.97
0.96
2.01
1.89
0.97
3.00
2.99
2.97
f1 (ppm)

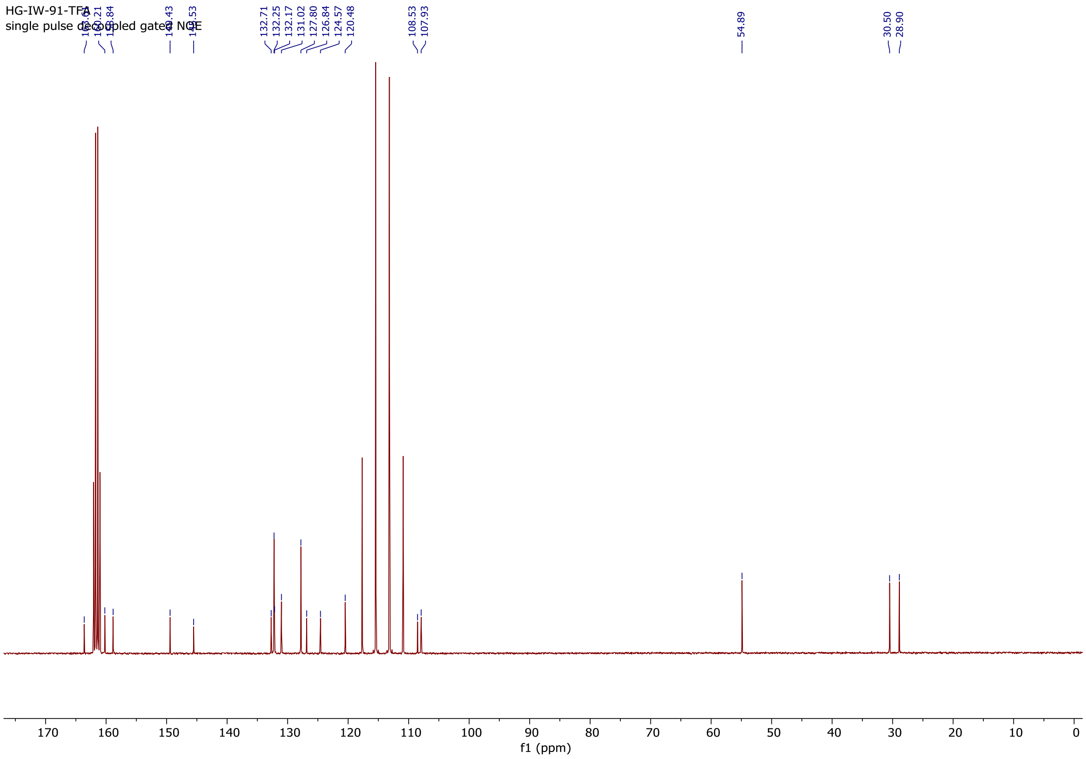


**Figure 13** $^{1}H$ and $^{13}C$ NMR spectra of **2k**

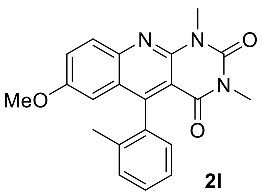

2l

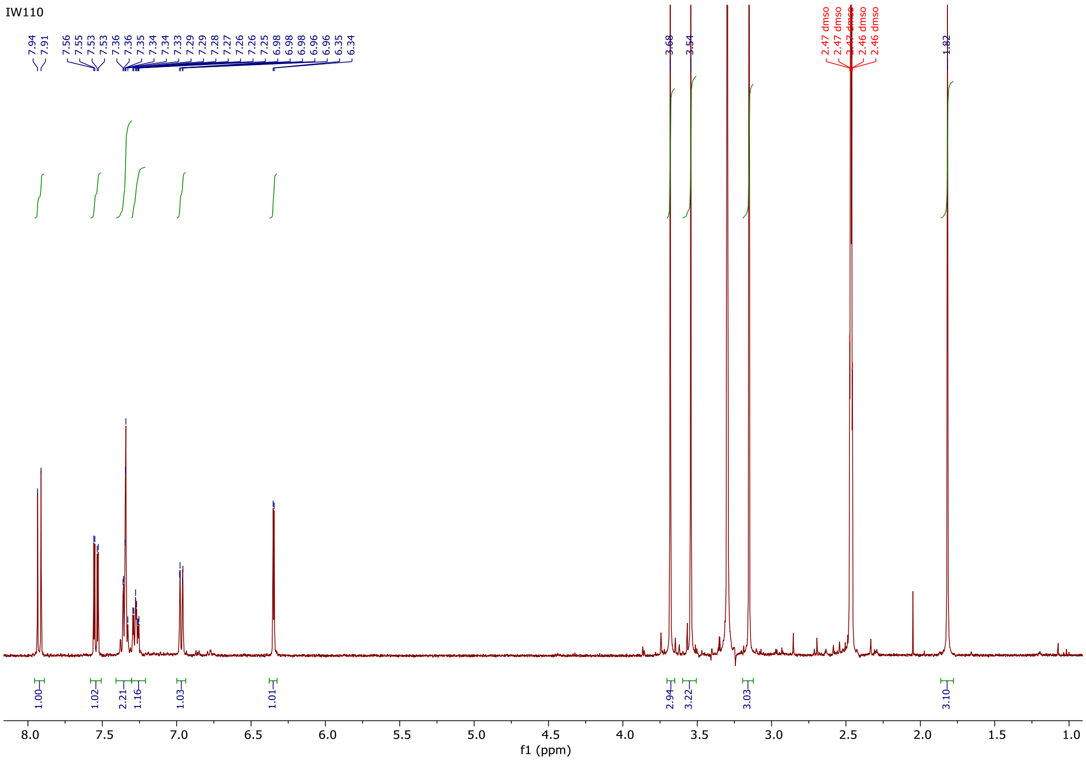


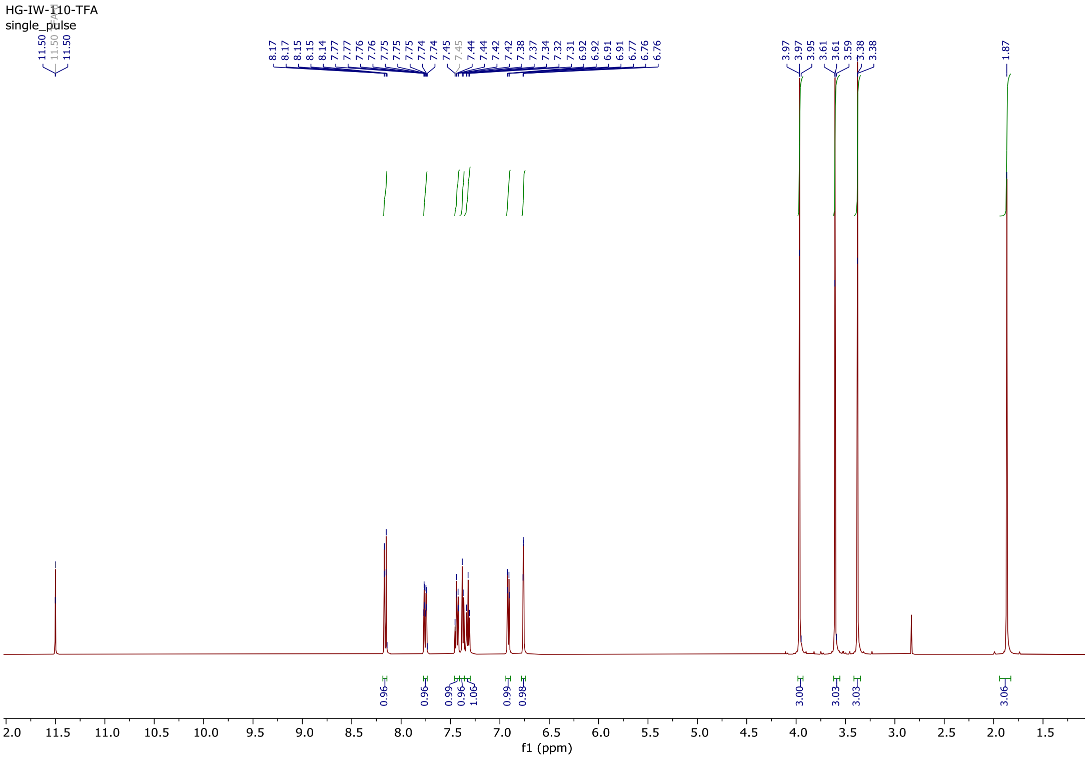

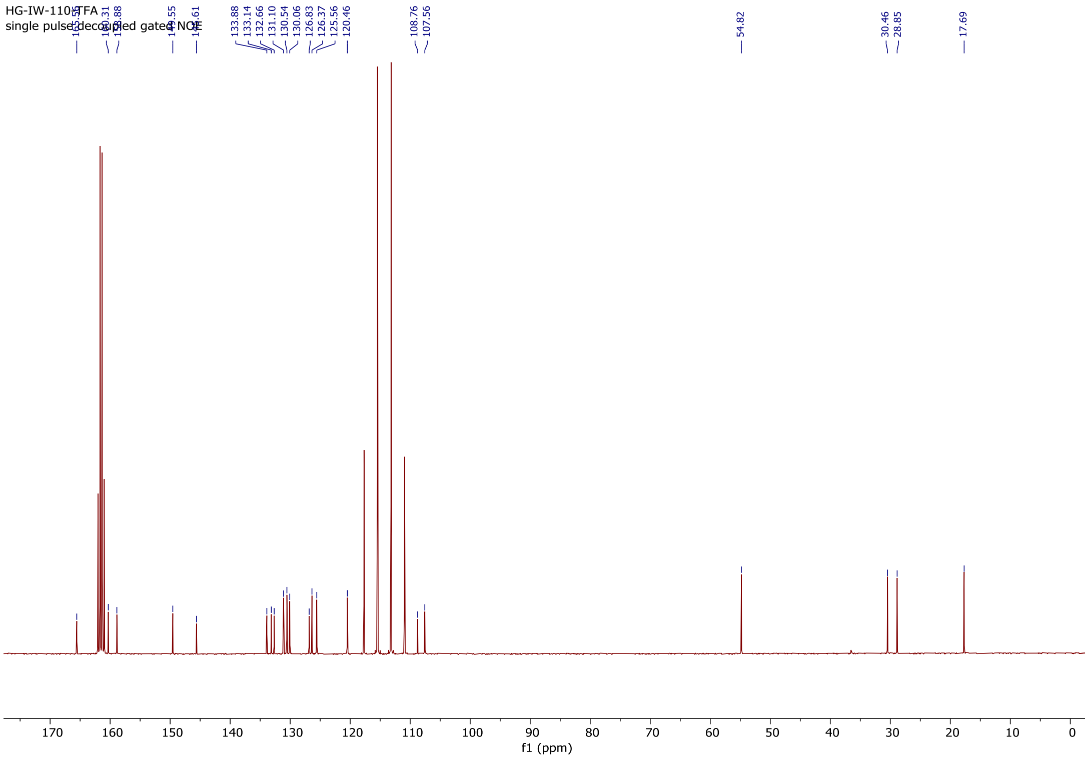


**Figure 14** $^{1}H$ and $^{13}C$ NMR spectra of **2l**

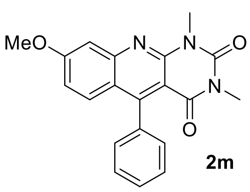
MeO
N
N
O
N
O

**2m**

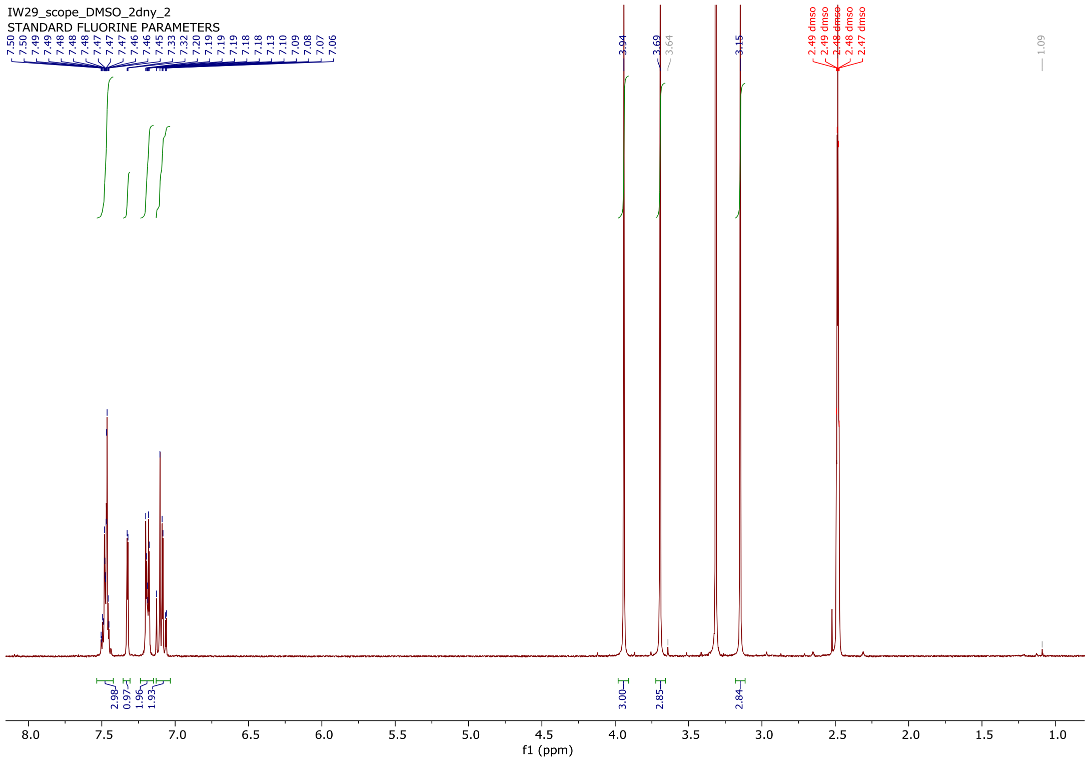
IW29_scope_DMSO_2dny_2
STANDARD FLUORINE PARAMETERS
7.50 7.50 7.49 7.48 7.48 7.48 7.47 7.47 7.47 7.46 7.46 7.45 7.33 7.32 7.20 7.19 7.19 7.19 7.18 7.18 7.13 7.10 7.09 7.08 7.07 7.06
3.94
3.69
3.64
3.15
2.49 dmso
2.49 dmso
2.48 dmso
2.48 dmso
2.47 dmso
1.09
2.98
0.97
1.96
1.93
3.00
2.85
2.84
8.0 7.5 7.0 6.5 6.0 5.5 5.0 4.5 4.0 3.5 3.0 2.5 2.0 1.5 1.0
f1 (ppm)

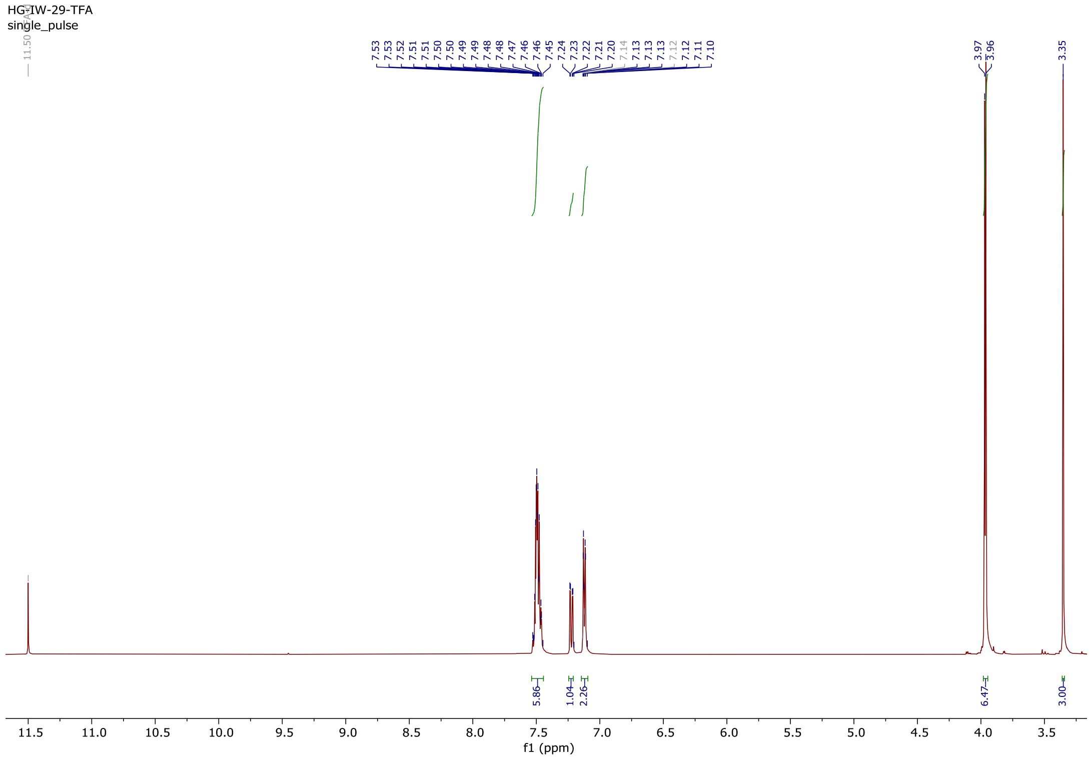
HG-IW-29-TFA
single_pulse
11.50
7.53 7.53 7.52 7.51 7.51 7.50 7.50 7.49 7.49 7.48 7.48 7.47 7.46 7.46 7.45 7.24 7.23 7.22 7.21 7.20 7.14 7.13 7.13 7.13 7.12 7.12 7.11 7.10
3.97
3.96
3.35
5.86
1.04
2.26
6.47
3.00
11.5 11.0 10.5 10.0 9.5 9.0 8.5 8.0 7.5 7.0 6.5 6.0 5.5 5.0 4.5 4.0 3.5
f1 (ppm)

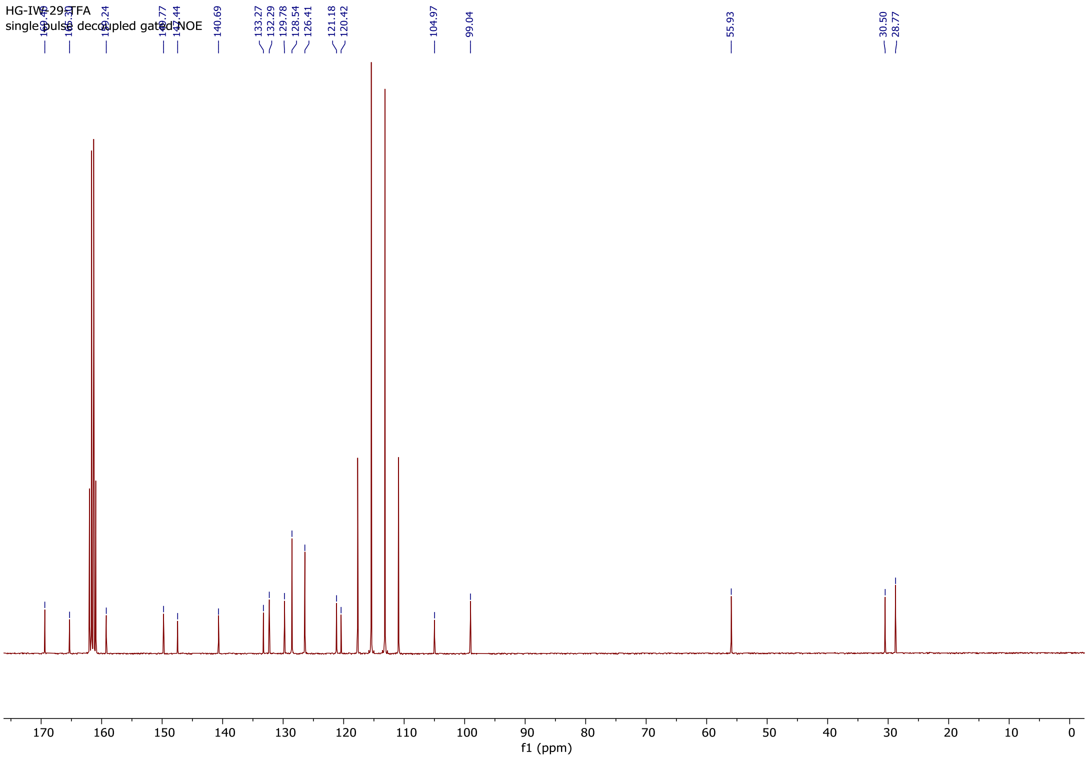


**Figure 15** $^{1}$H and $^{13}$C NMR spectra of **2m**

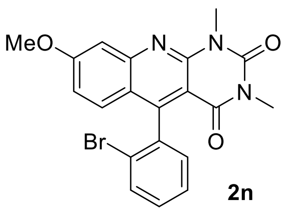
MeO
N
N
O
N
Br
O

**2n**

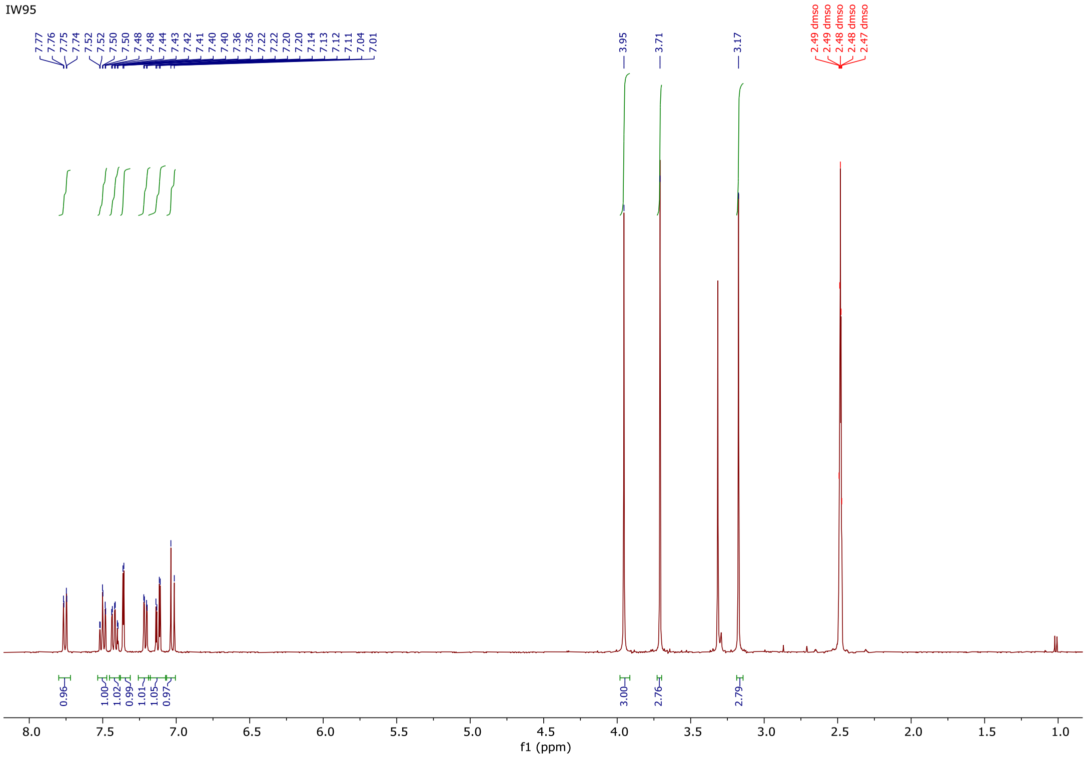
IW95
7.77
7.76
7.75
7.74
7.52
7.52
7.50
7.50
7.48
7.48
7.44
7.43
7.42
7.41
7.40
7.40
7.36
7.36
7.22
7.22
7.20
7.20
7.14
7.13
7.12
7.11
7.04
7.01
3.95
3.71
3.17
2.49 dmso
2.49 dmso
2.48 dmso
2.48 dmso
2.47 dmso
0.96
1.00
1.02
0.99
1.01
1.05
0.97
3.00
2.76
2.79
f1 (ppm)

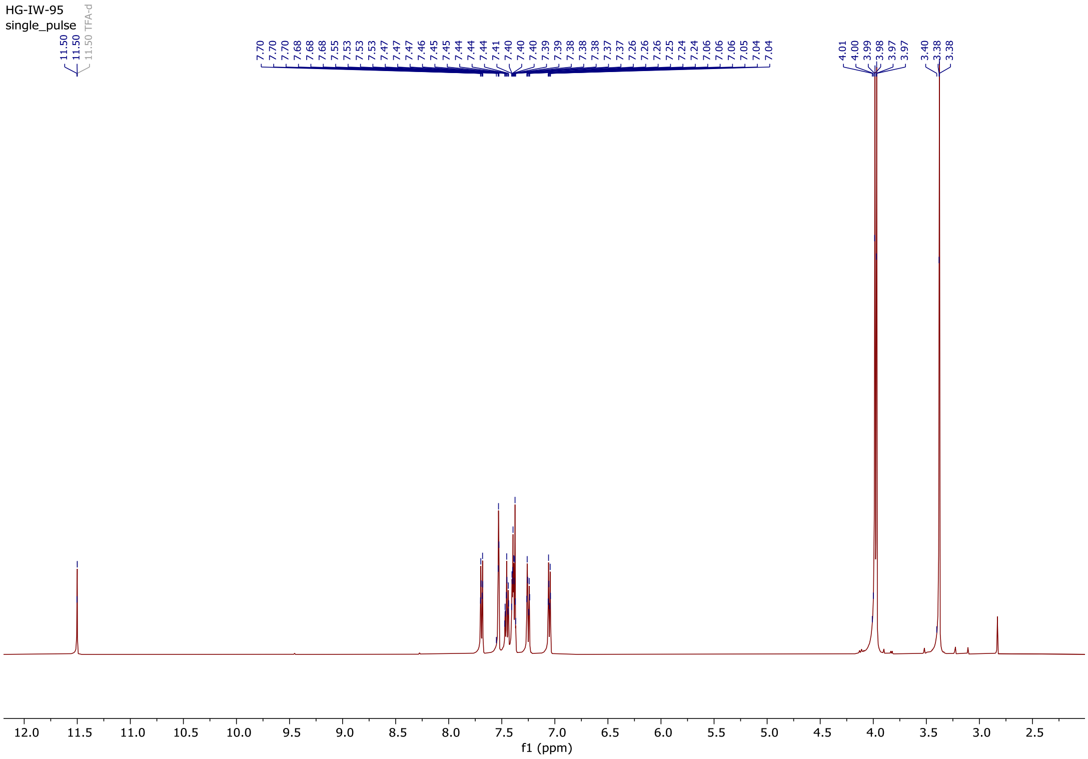
HG-IW-95
single_pulse
11.50
11.50
11.50 TFA-d
7.70
7.70
7.70
7.68
7.68
7.55
7.53
7.53
7.53
7.47
7.47
7.46
7.45
7.45
7.44
7.44
7.41
7.40
7.40
7.40
7.39
7.38
7.38
7.37
7.37
7.26
7.26
7.26
7.25
7.24
7.24
7.06
7.06
7.06
7.05
7.04
7.04
4.01
4.00
3.99
3.98
3.97
3.97
3.40
3.38
3.38
f1 (ppm)

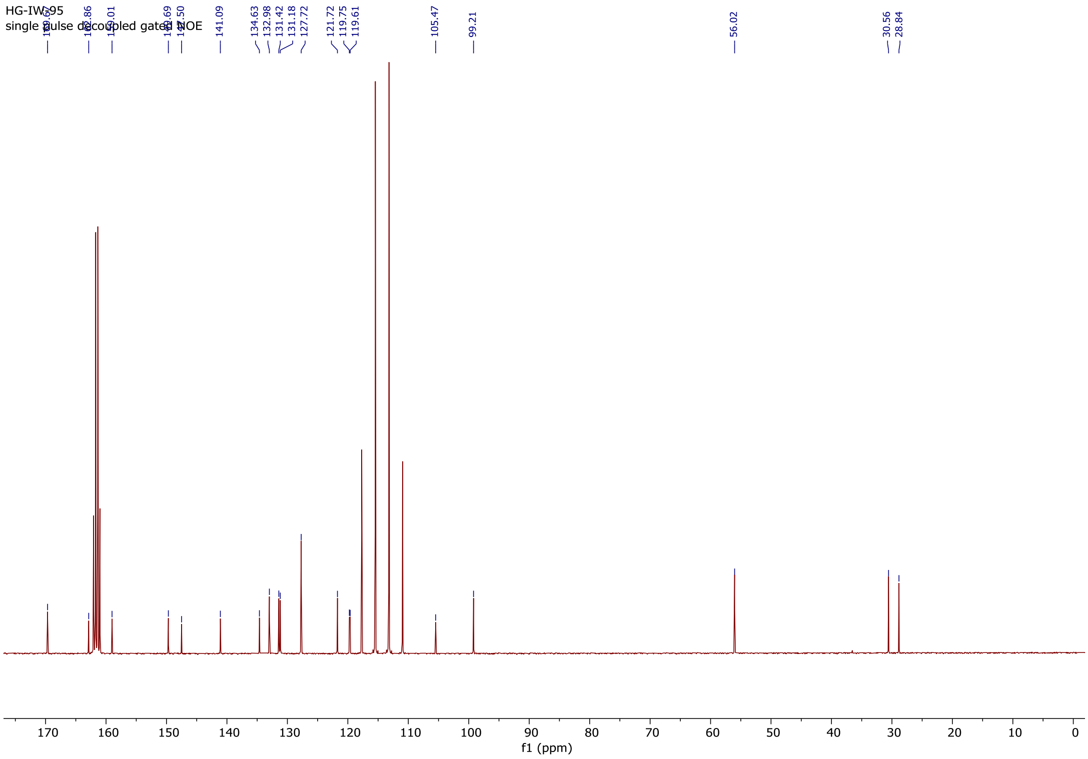


**Figure 16** $^{1}H$ and $^{13}C$ NMR spectra of **2n**

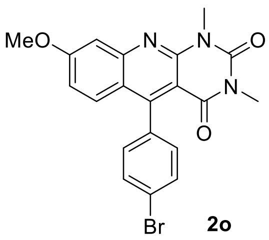


**2o**

IW44_scope_DMSO_3dny

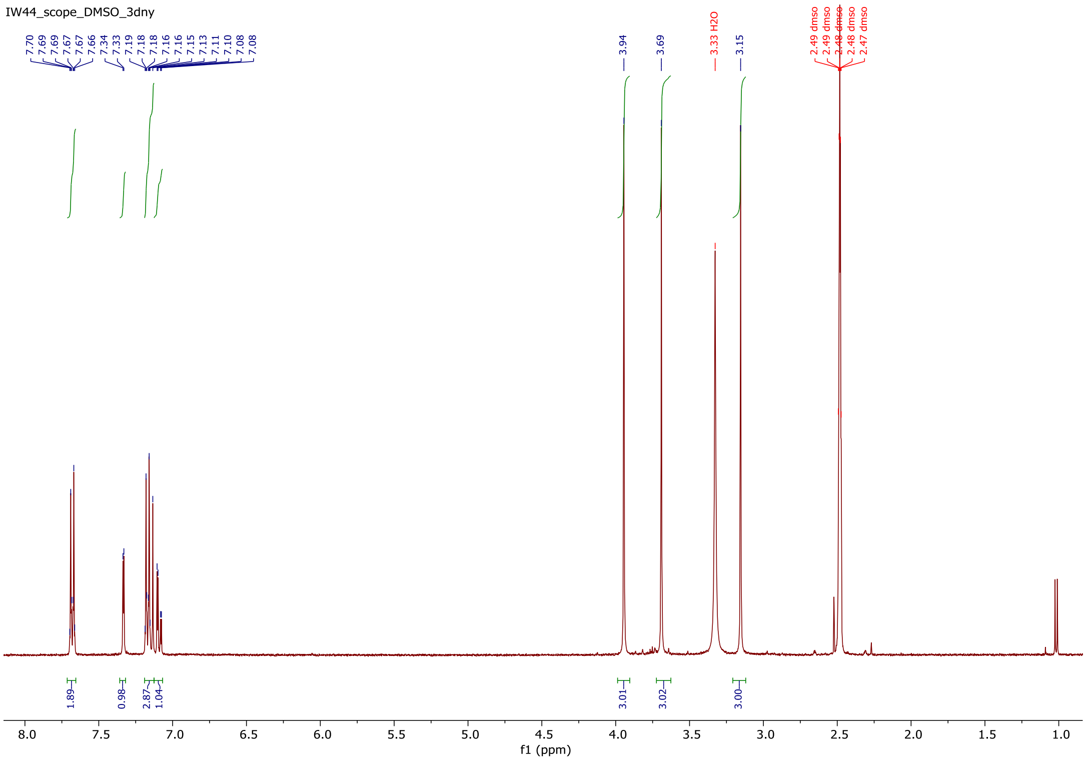


HG-IW-44-TFA
single_pulse

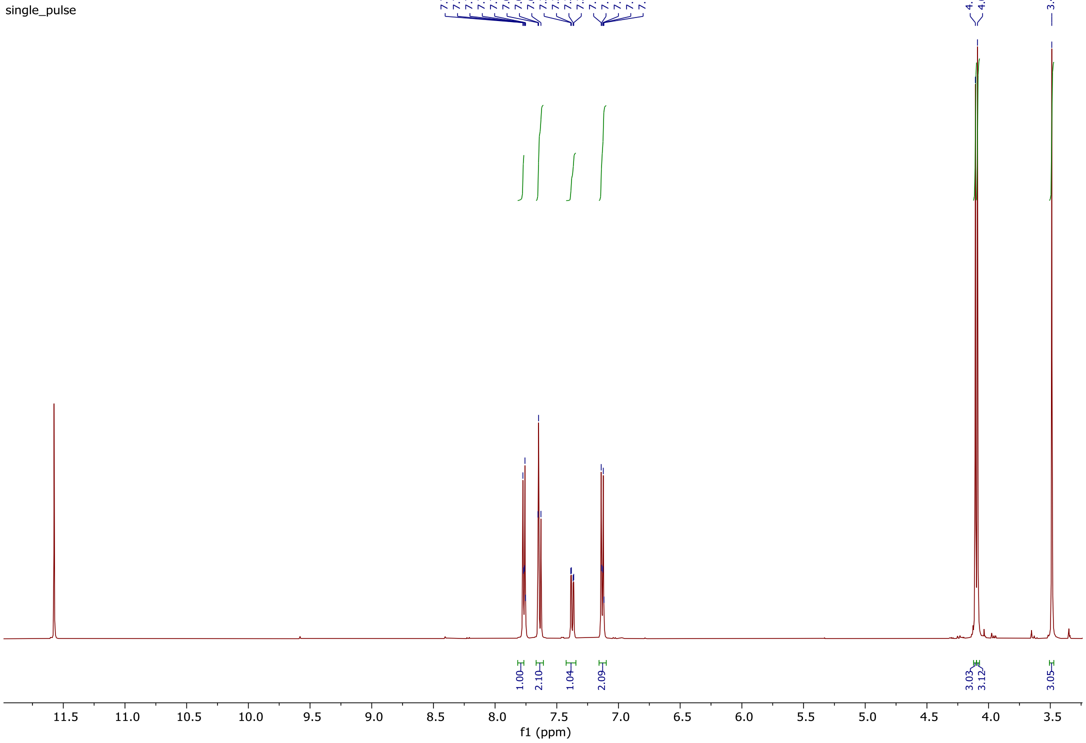

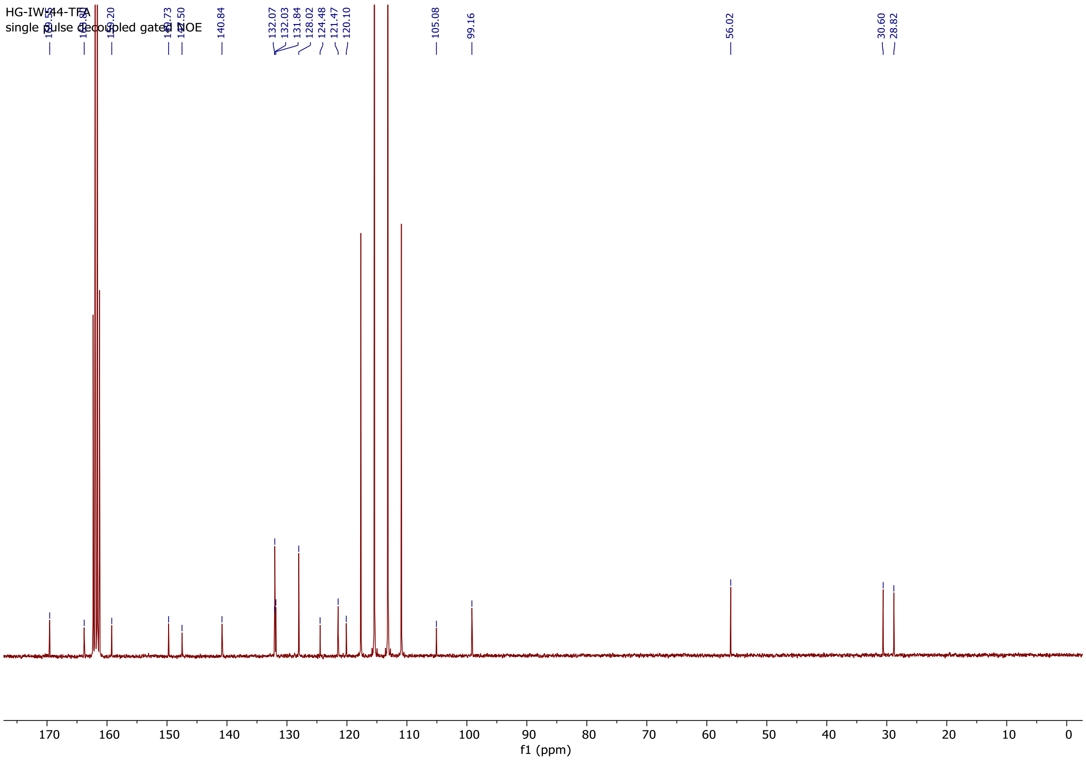


**Figure 17** $^{1}$H and $^{13}$C NMR spectra of **2o**

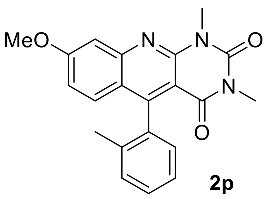

**2p**

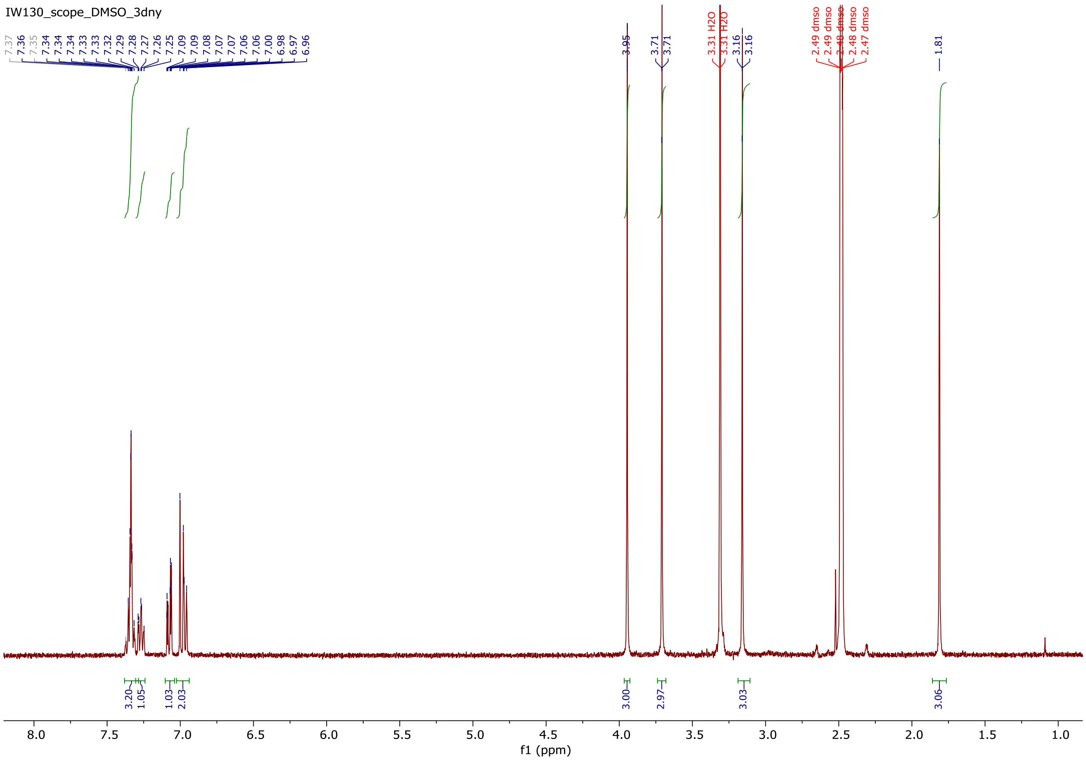


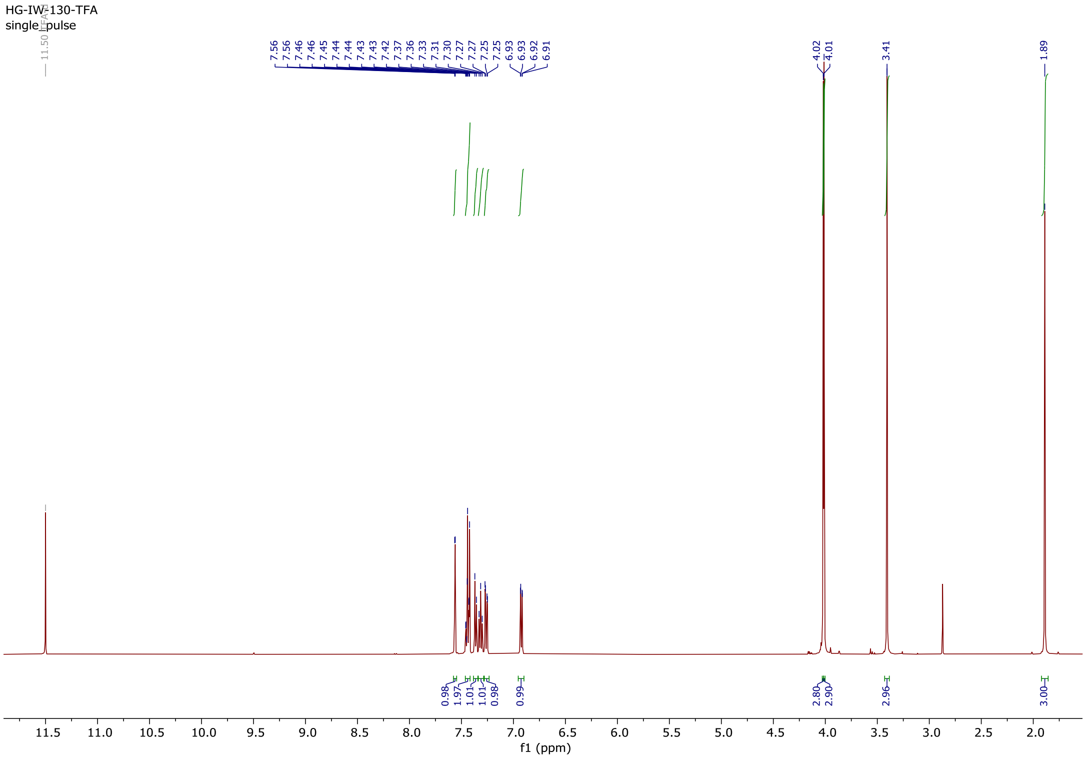

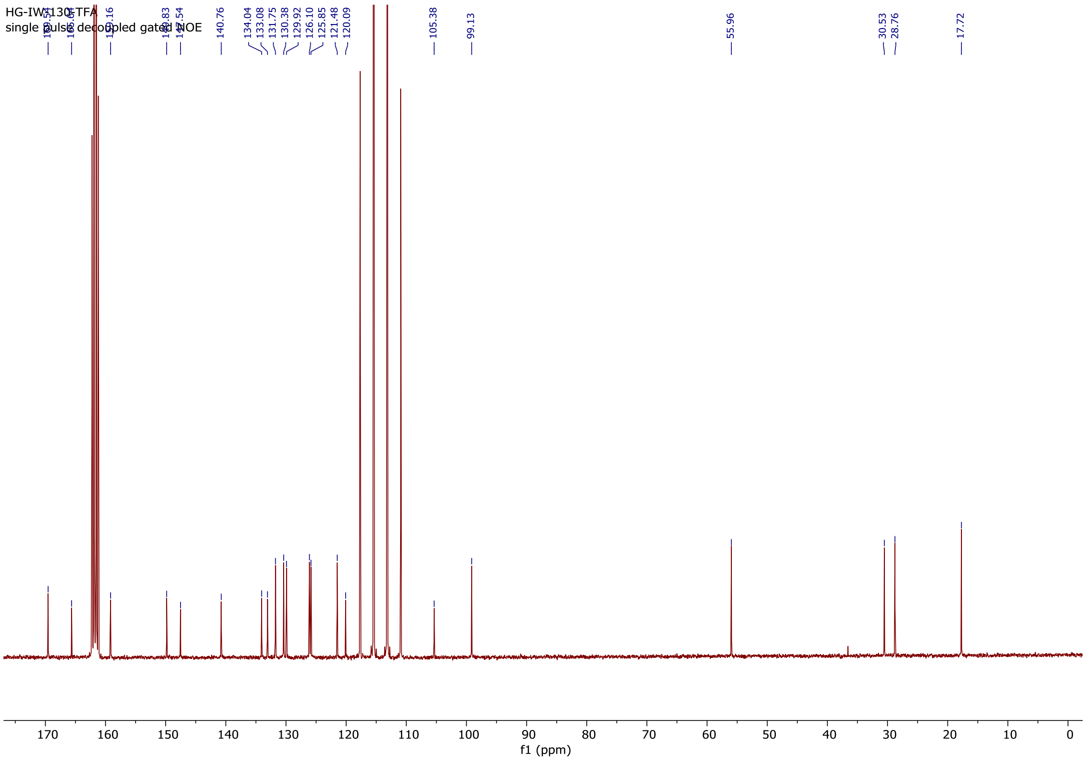


**Figure 18** $^{1}$H and $^{13}$C NMR spectra of **2p**

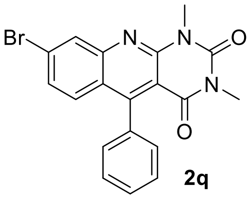

**2q**

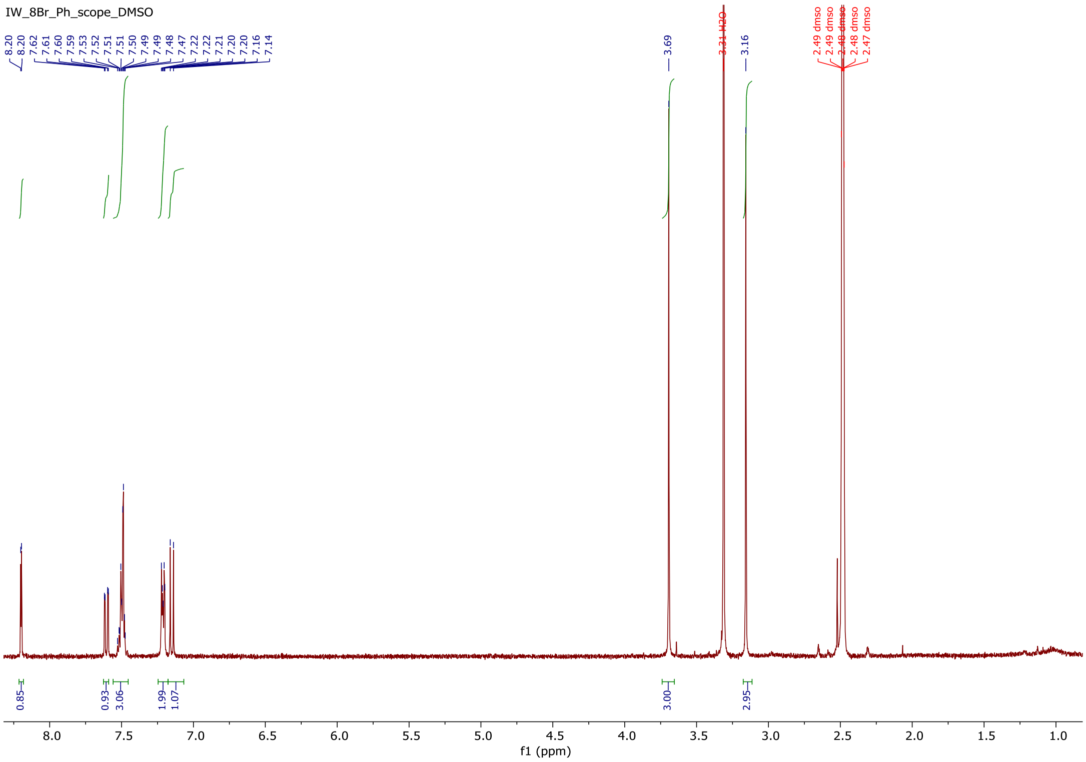
IW_8Br_Ph_scope_DMSO
8.20
8.20
7.62
7.61
7.60
7.59
7.53
7.52
7.51
7.51
7.50
7.49
7.49
7.48
7.47
7.22
7.22
7.21
7.20
7.16
7.14
3.69
3.31 H2O
3.16
2.49 dmso
2.49 dmso
2.49 dmso
2.48 dmso
2.47 dmso
0.85
0.93
3.06
1.99
1.07
3.00
2.95
8.0 7.5 7.0 6.5 6.0 5.5 5.0 4.5 4.0 3.5 3.0 2.5 2.0 1.5 1.0
f1 (ppm)

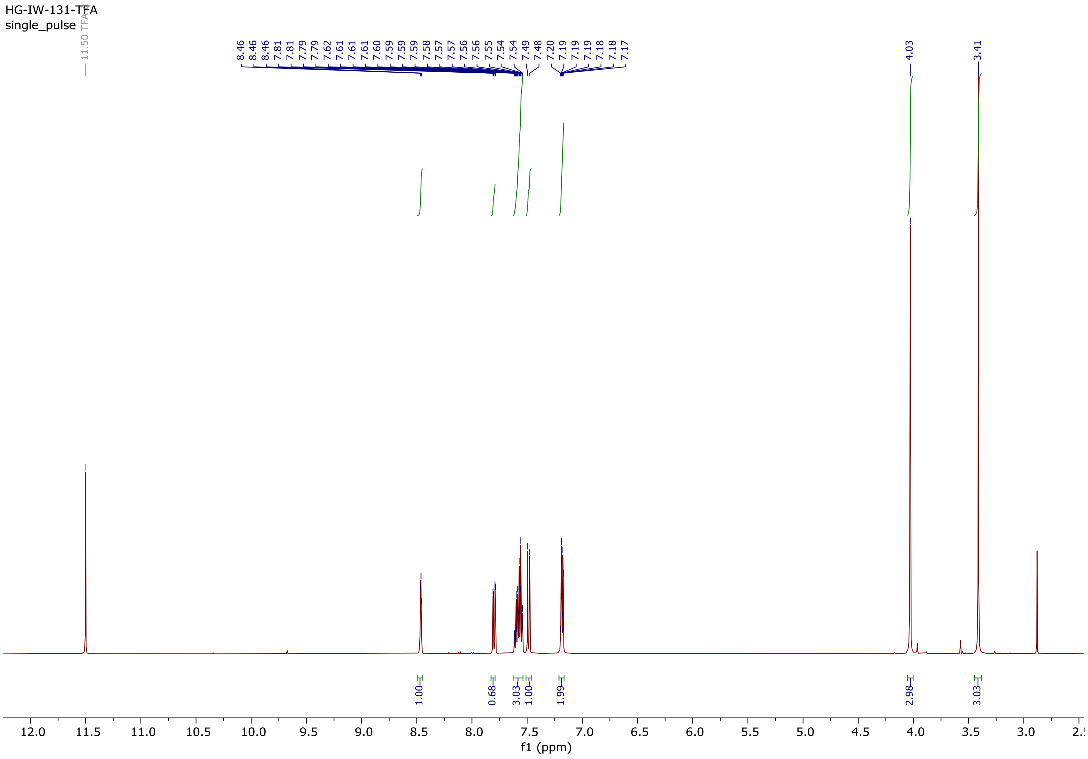
HG-IW-131-TFA
single_pulse
11.50 TFA
8.46
8.46
8.46
7.81
7.81
7.79
7.79
7.62
7.61
7.61
7.61
7.60
7.59
7.59
7.59
7.58
7.57
7.57
7.56
7.55
7.54
7.49
7.48
7.20
7.19
7.19
7.19
7.18
7.17
4.03
3.41
1.00
0.68
3.03
1.00
1.99
2.98
3.03
12.0 11.5 11.0 10.5 10.0 9.5 9.0 8.5 8.0 7.5 7.0 6.5 6.0 5.5 5.0 4.5 4.0 3.5 3.0
f1 (ppm)

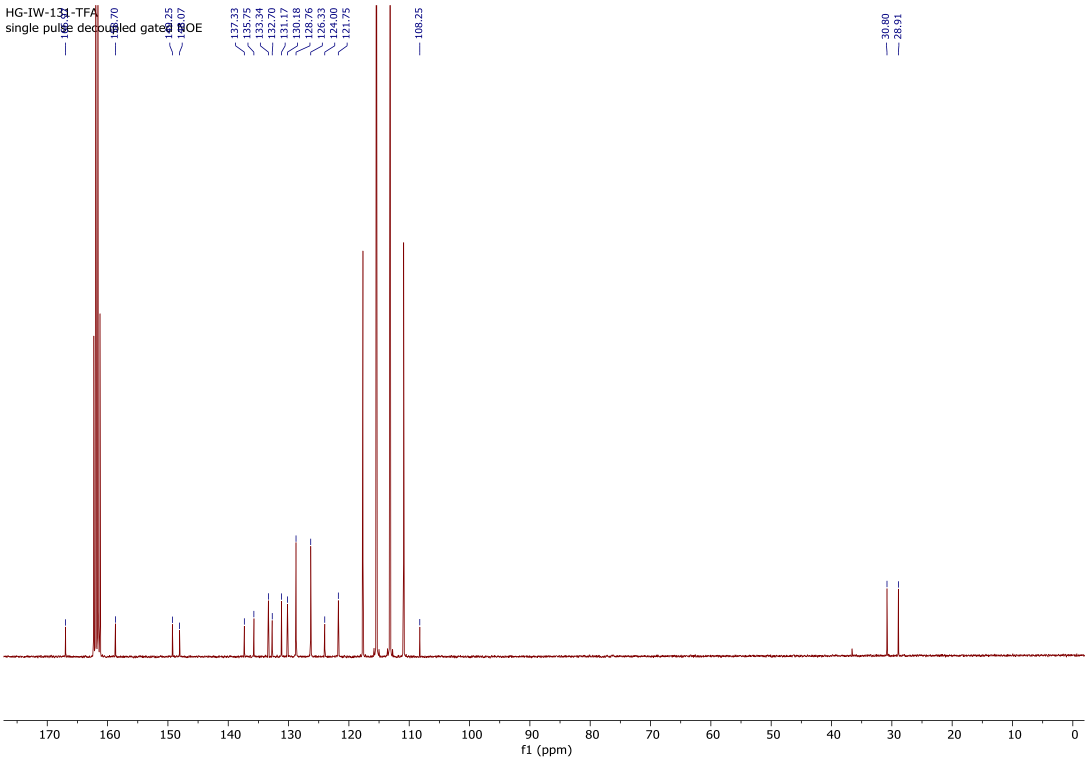


**Figure 19** $^{1}$H and $^{13}$C NMR spectra of **2q**

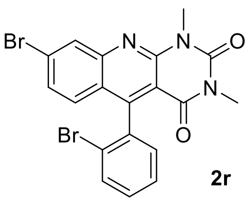

**2r**

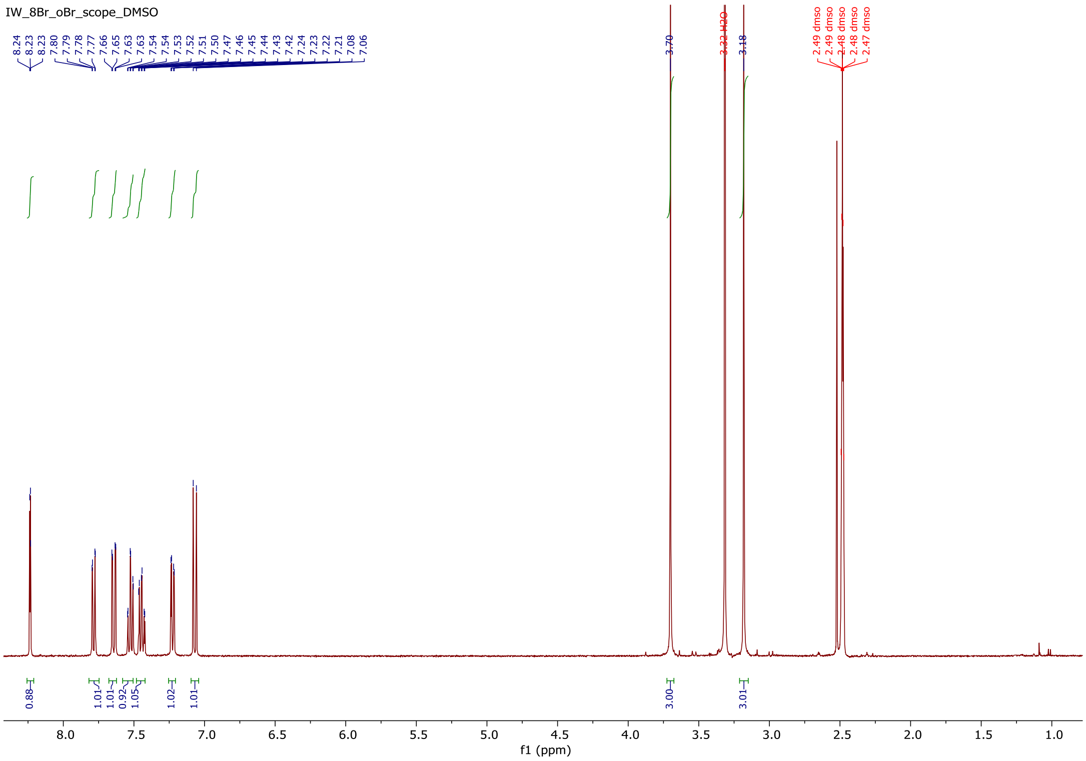


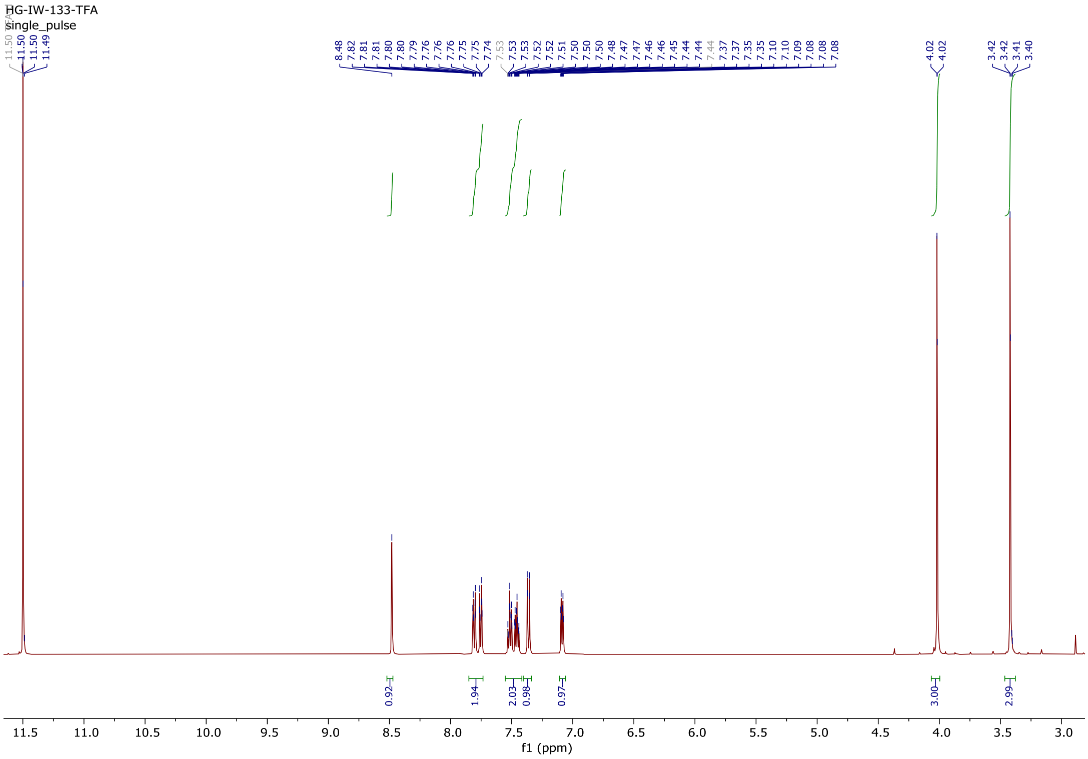

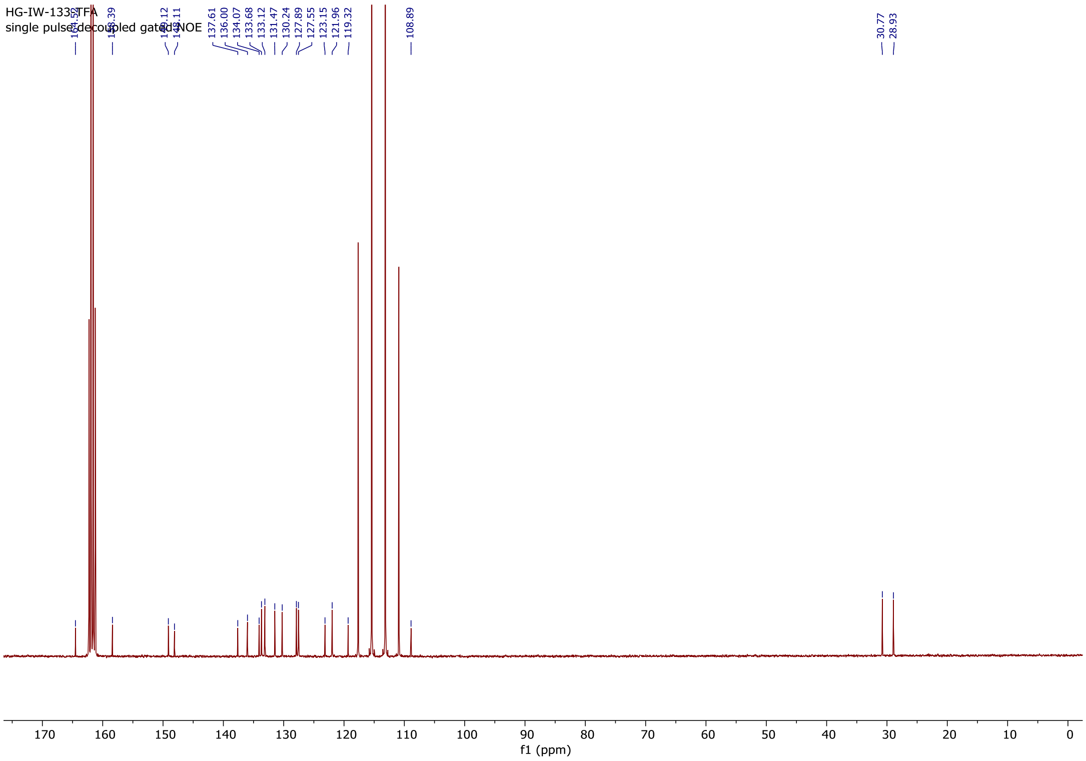


**Figure 20** $^{1}H$ and $^{13}C$ NMR spectra of **2r**

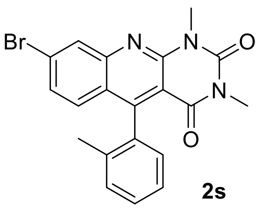

**2s**

IW_8Br_oMe_scope_DMSO

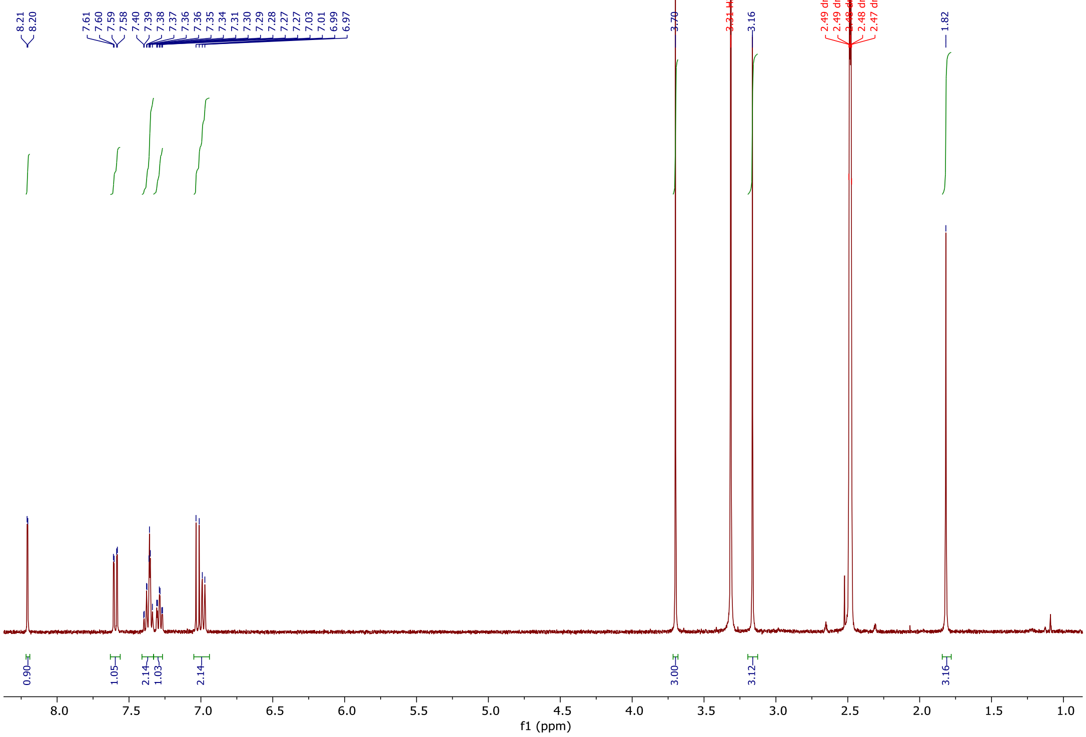


HG-IW-132-TFA
single_pulse

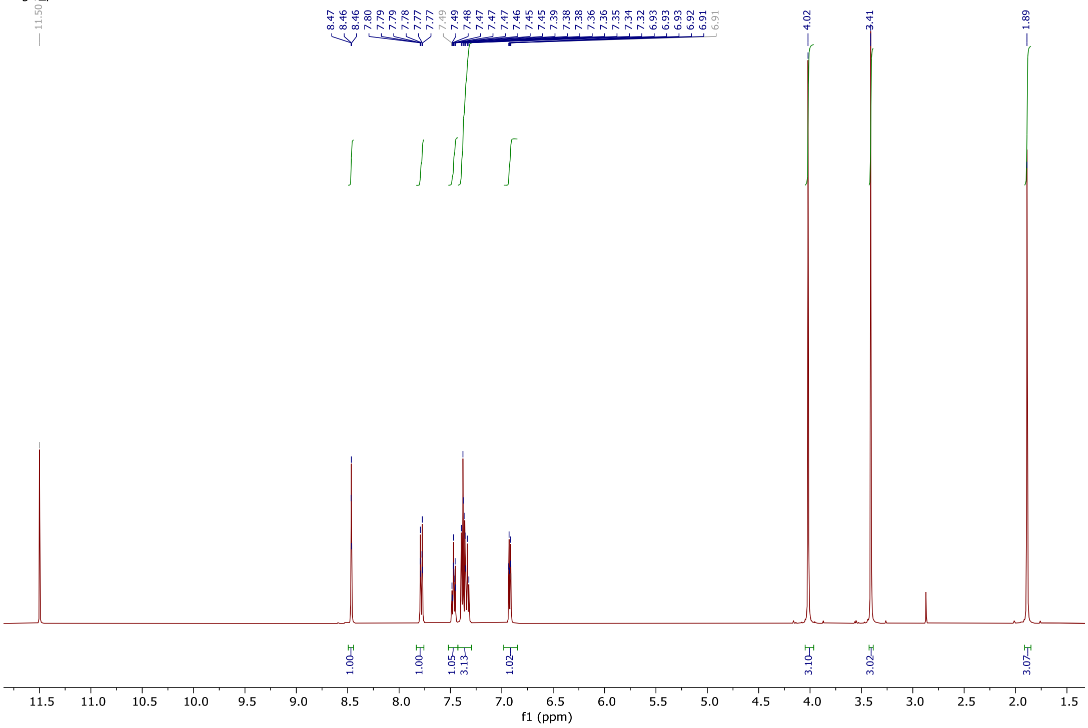

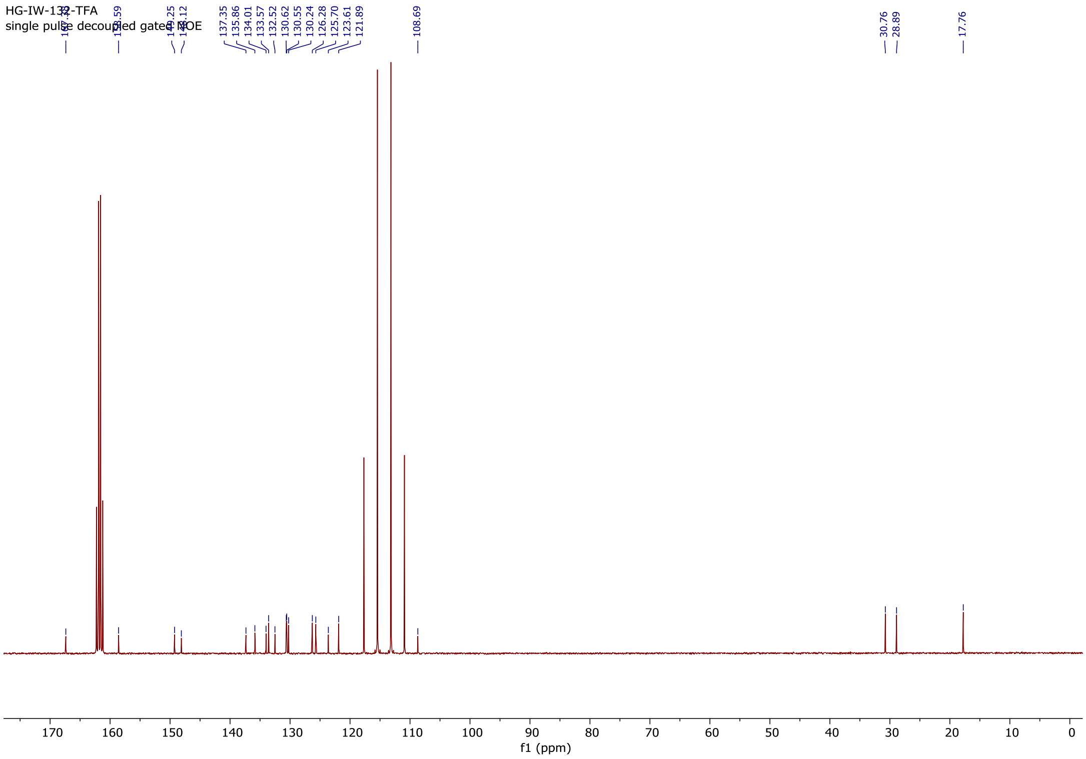


**Figure 21** $^{1}H$ and $^{13}C$ NMR spectra of **2s**

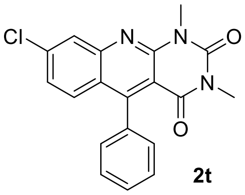

**2t**

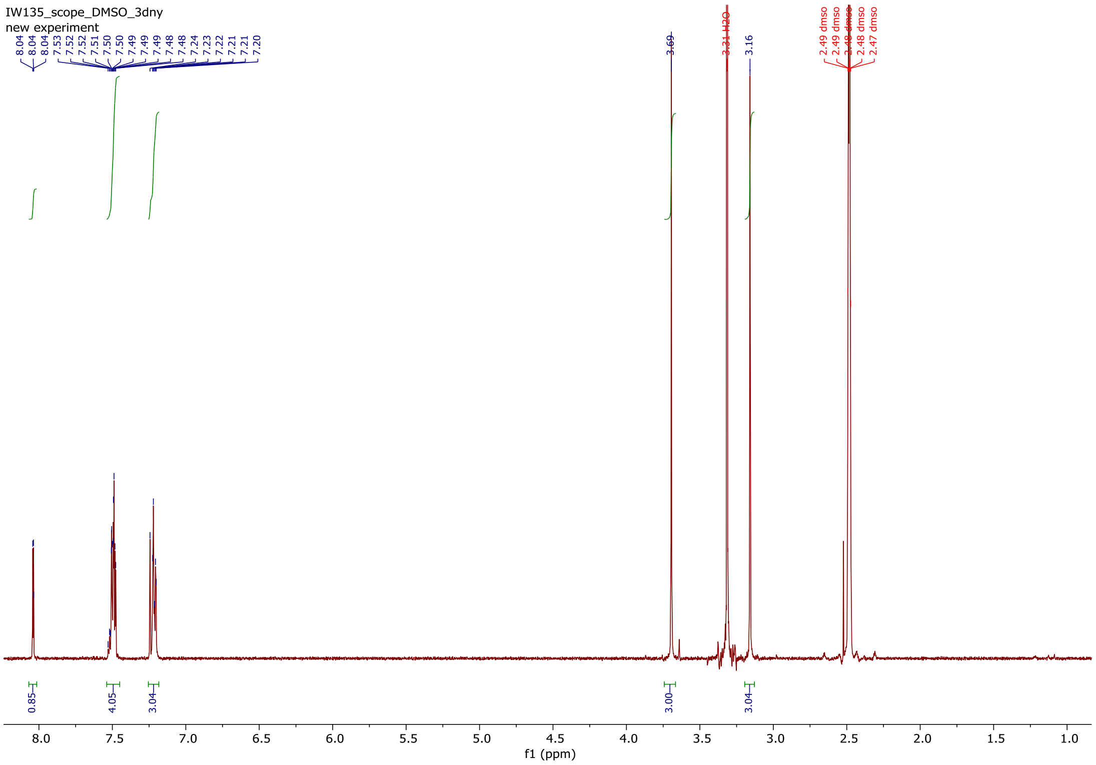
IW135_scope_DMSO_3dny
new experiment
f1 (ppm)

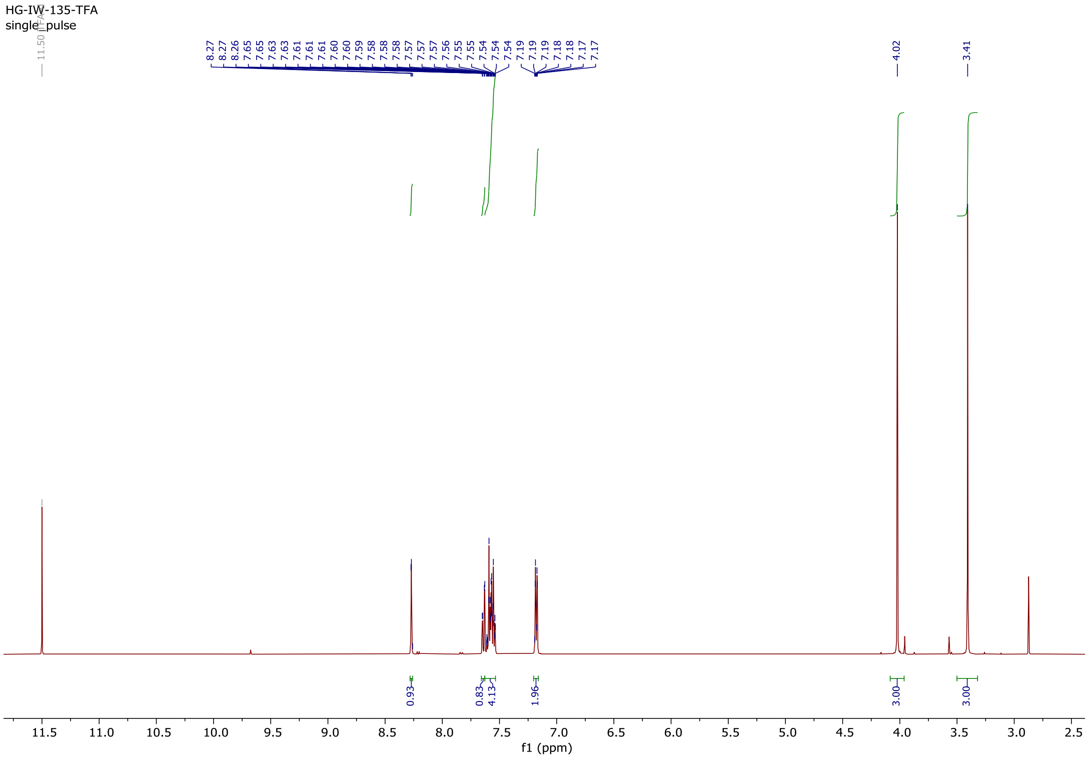
HG-IW-135-TFA
single_pulse
f1 (ppm)

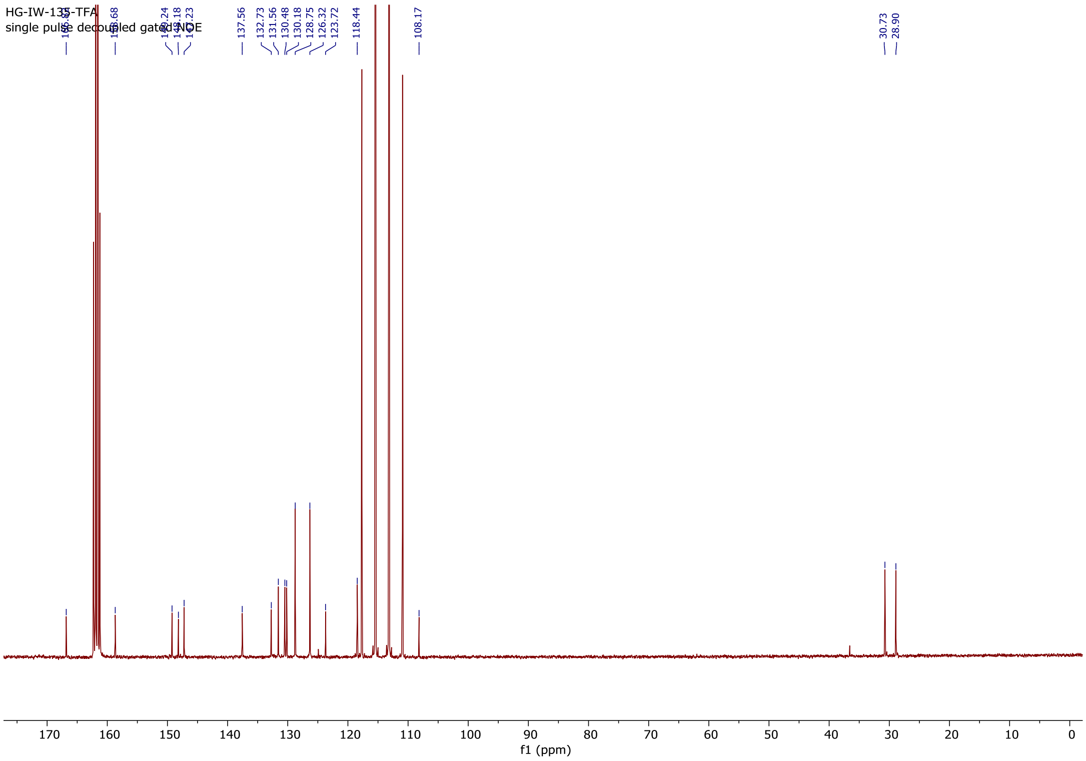


**Figure 22** $^{1}H$ and $^{13}C$ NMR spectra of **2t**

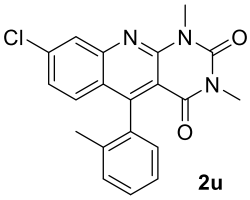
Cl
N
N
O
N
O

**2u**

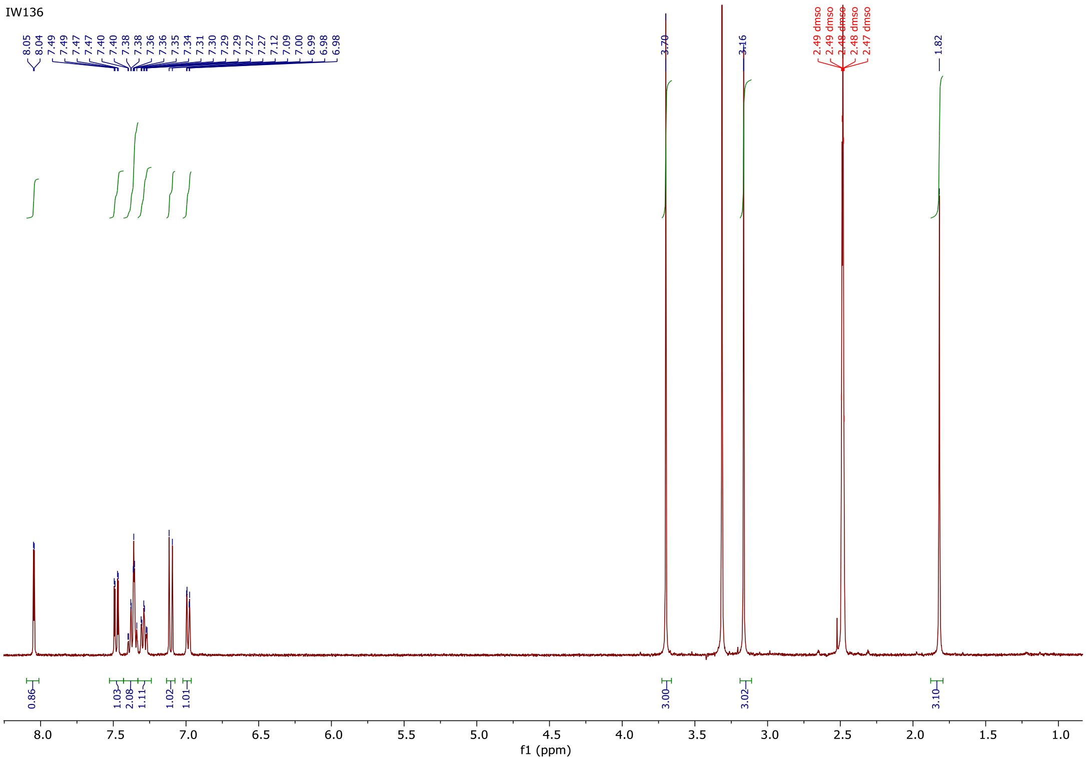
IW136
8.05 8.04 7.49 7.47 7.47 7.40 7.40 7.38 7.38 7.36 7.36 7.35 7.34 7.31 7.30 7.29 7.29 7.27 7.27 7.12 7.09 7.00 6.99 6.98 6.98
3.70
3.16
2.49 dmso 2.49 dmso 2.48 dmso 2.48 dmso 2.47 dmso
1.82
0.86 1.03 2.08 1.11 1.02 1.01 3.00 3.02 3.10
8.0 7.5 7.0 6.5 6.0 5.5 5.0 4.5 4.0 3.5 3.0 2.5 2.0 1.5 1.0
f1 (ppm)

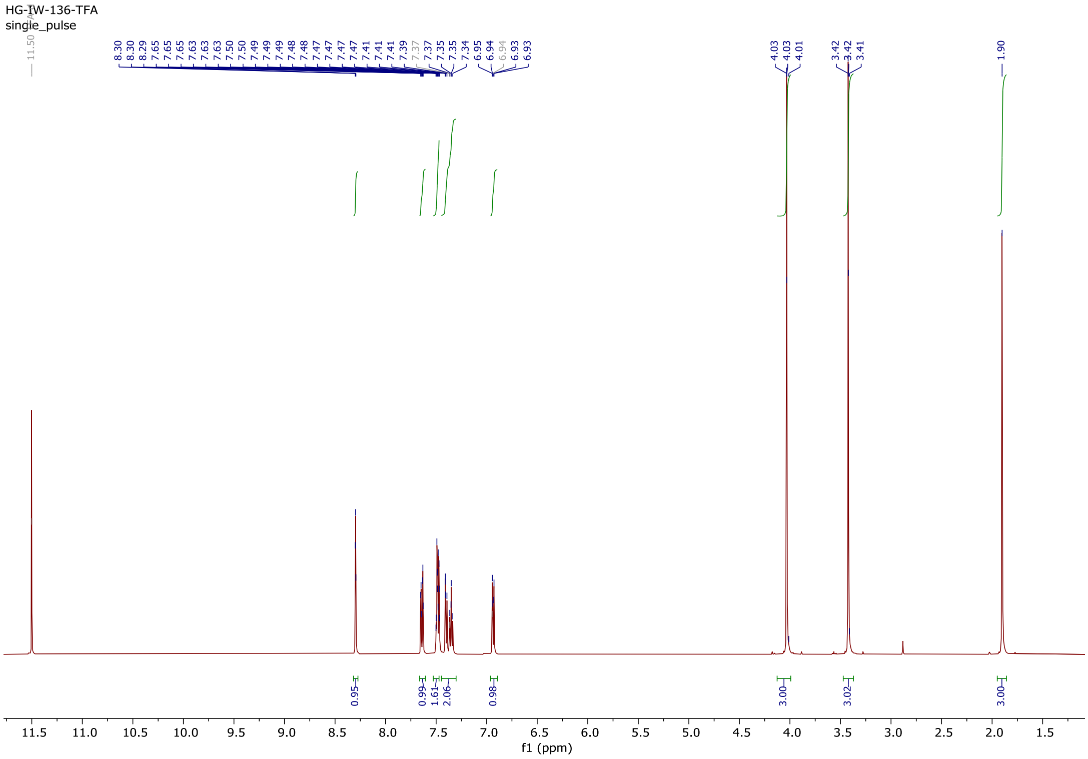
HG-IW-136-TFA
single_pulse
11.50
8.30 8.30 8.29 7.65 7.65 7.65 7.63 7.63 7.63 7.50 7.49 7.49 7.49 7.48 7.47 7.47 7.47 7.47 7.41 7.41 7.41 7.39 7.37 7.37 7.35 7.35 7.34 6.95 6.94 6.94 6.93 6.93
4.03 4.03 4.01
3.42 3.42 3.41
1.90
0.95 0.99 1.61 2.06 0.98 3.00 3.02 3.00
11.5 11.0 10.5 10.0 9.5 9.0 8.5 8.0 7.5 7.0 6.5 6.0 5.5 5.0 4.5 4.0 3.5 3.0 2.5 2.0 1.5
f1 (ppm)

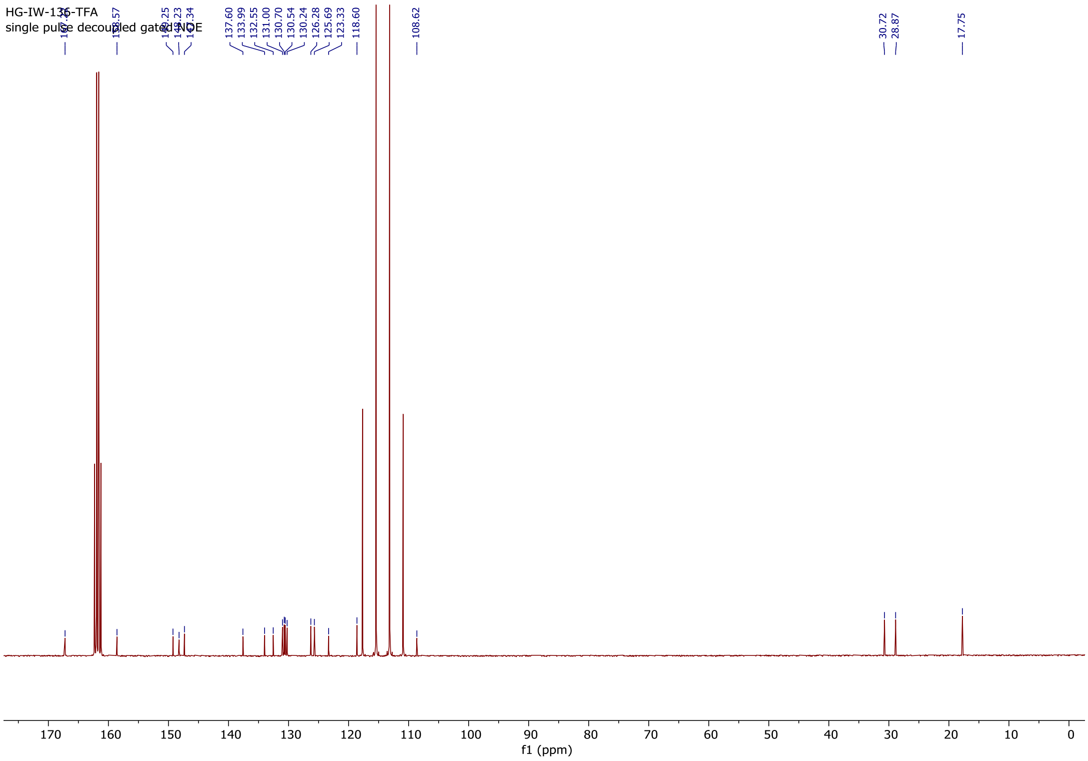


**Figure 23** $^{1}H$ and $^{13}C$ NMR spectra of **2u**

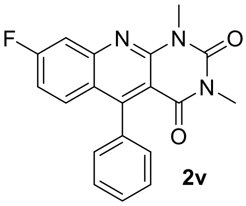

**2v**

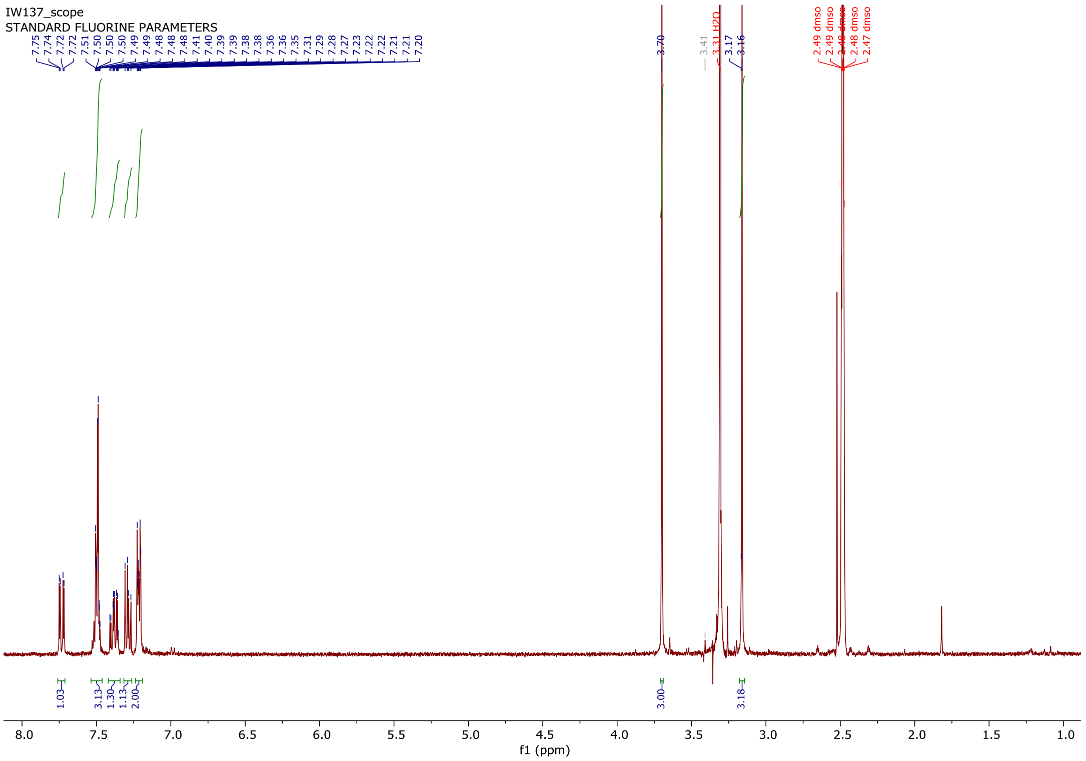
IW137_scope
STANDARD FLUORINE PARAMETERS
f1 (ppm)

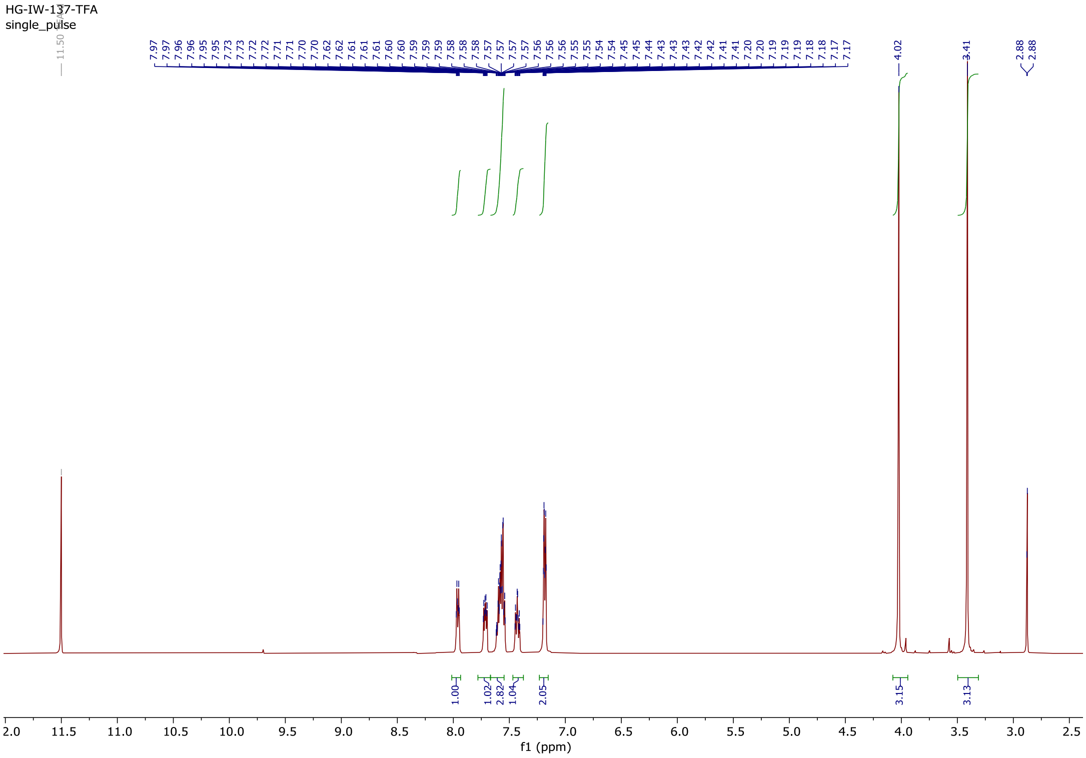
HG-IW-137-TFA
single_pulse
f1 (ppm)

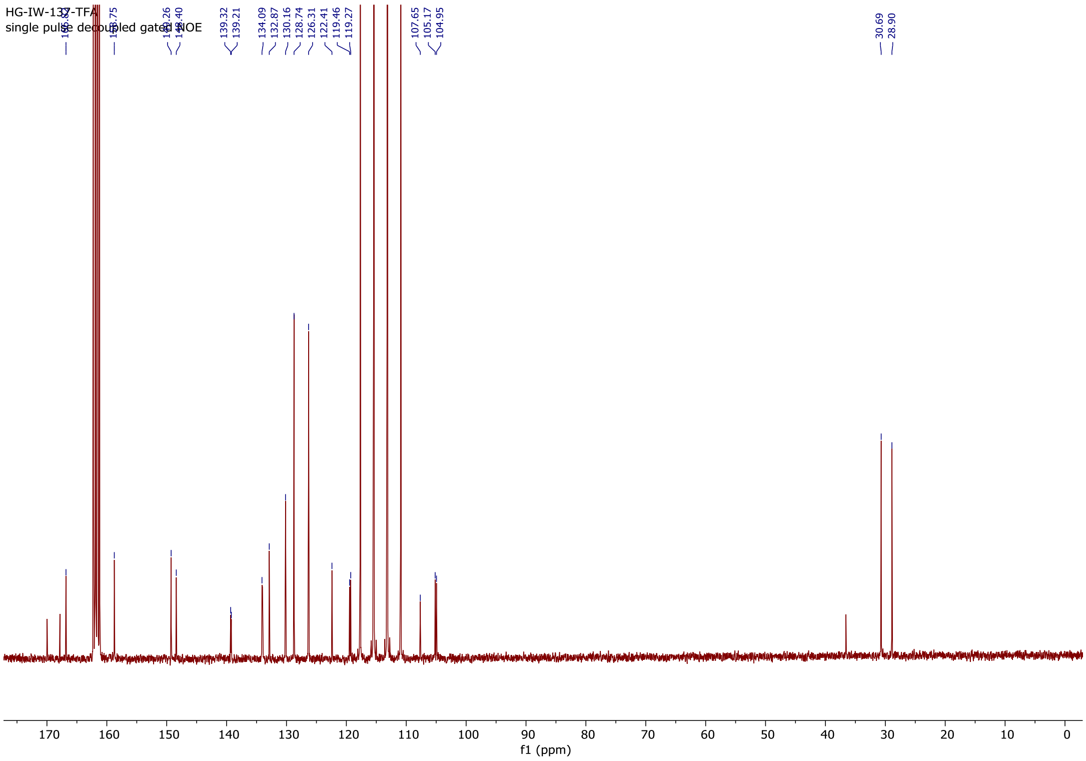


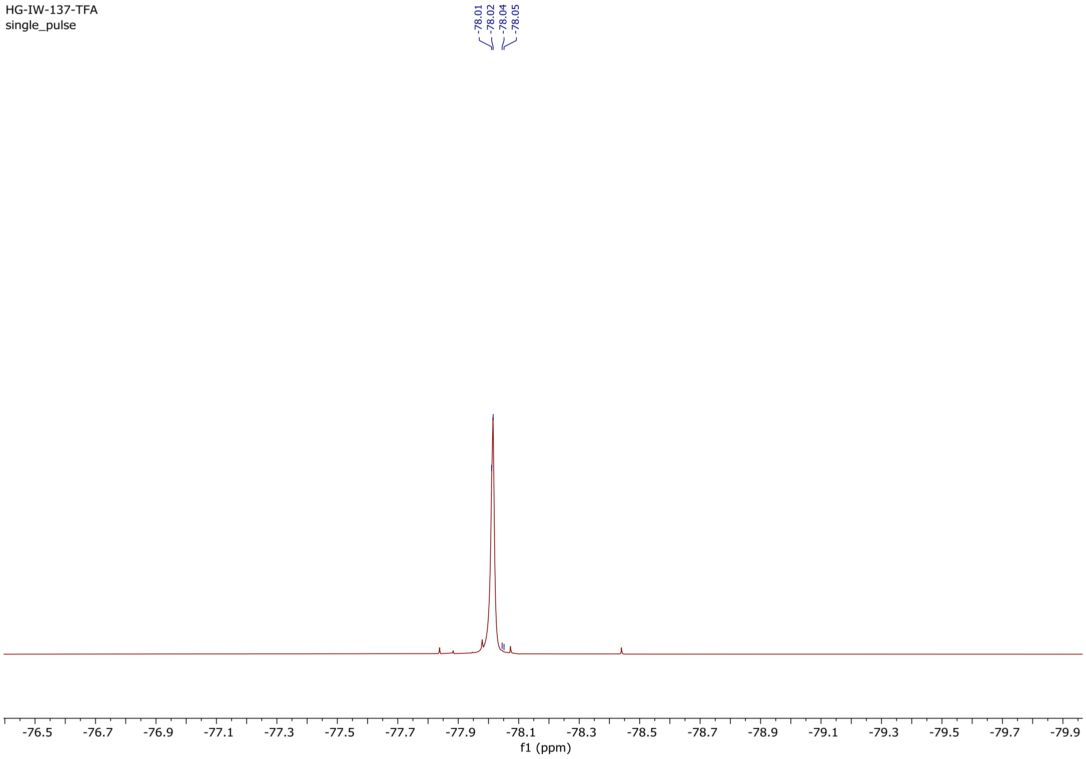


**Figure 24** $^{1}H$, $^{13}C$ NMR and $^{19}F$ spectra of **2v**

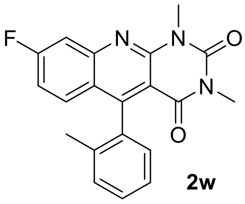

**2w**

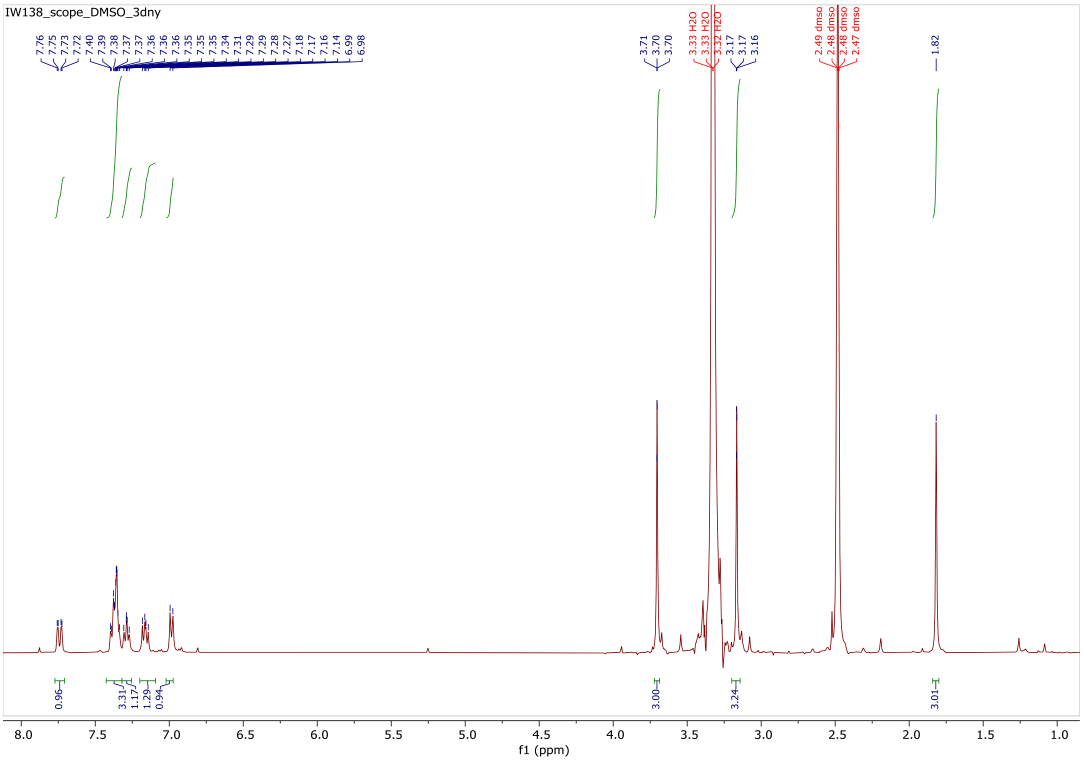
IW138_scope_DMSO_3dny
7.76 7.75 7.73 7.72 7.40 7.39 7.38 7.37 7.37 7.36 7.36 7.36 7.35 7.35 7.35 7.34 7.31 7.29 7.29 7.28 7.27 7.18 7.17 7.16 7.14 6.99 6.98
3.71 3.70 3.70
3.33 H2O 3.33 H2O 3.32 H2O
3.17 3.17 3.16
2.49 dmso 2.48 dmso 2.48 dmso 2.47 dmso
1.82
0.96 3.31 1.17 1.29 0.94 3.00 3.24 3.01
f1 (ppm)

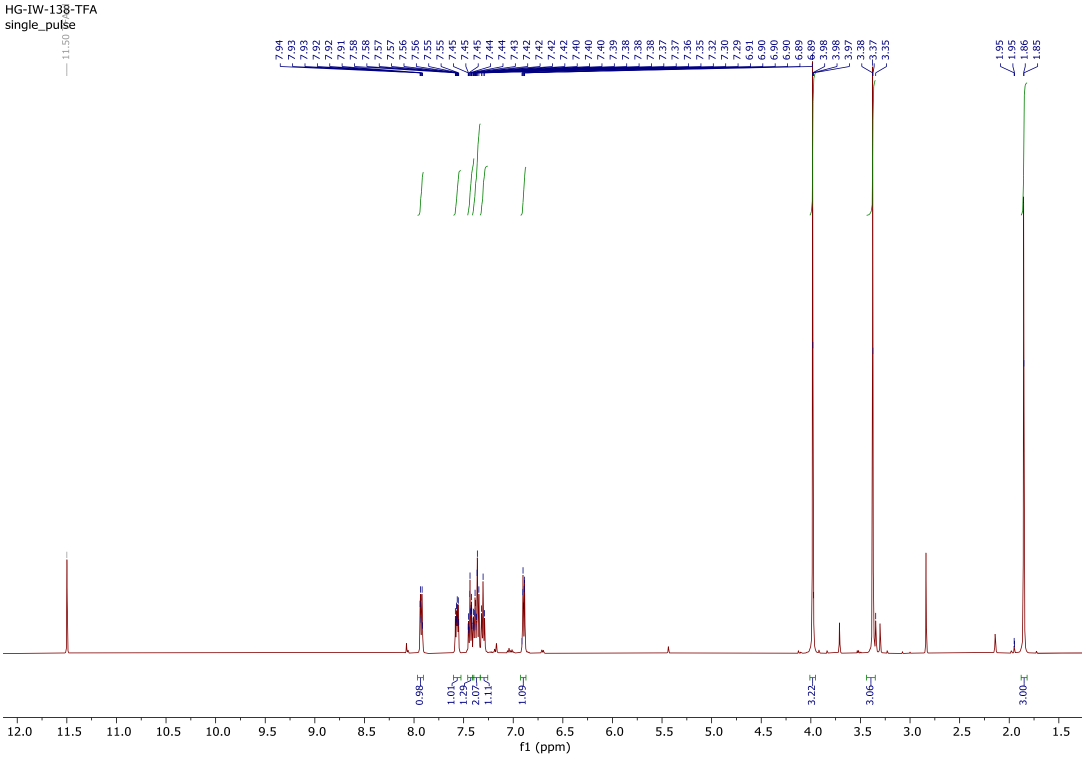
HG-IW-138-TFA
single_pulse
11.50
7.94 7.93 7.92 7.92 7.91 7.58 7.57 7.57 7.56 7.56 7.55 7.45 7.45 7.45 7.44 7.43 7.42 7.42 7.42 7.42 7.40 7.40 7.40 7.39 7.38 7.38 7.38 7.37 7.37 7.36 7.35 7.32 7.30 7.29 6.91 6.90 6.90 6.89 6.89 3.98 3.98 3.97 3.38 3.37 3.35
1.95 1.95 1.86 1.85
0.98 1.01 1.29 2.07 1.11 1.09 3.22 3.06 3.00
f1 (ppm)

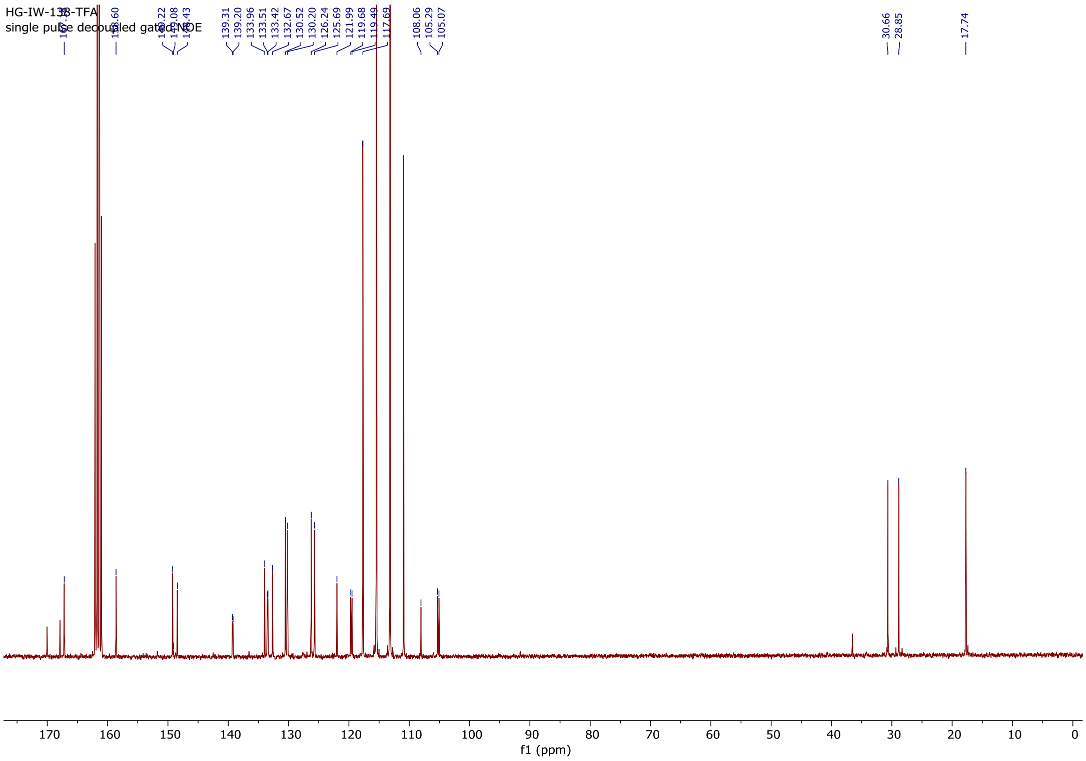


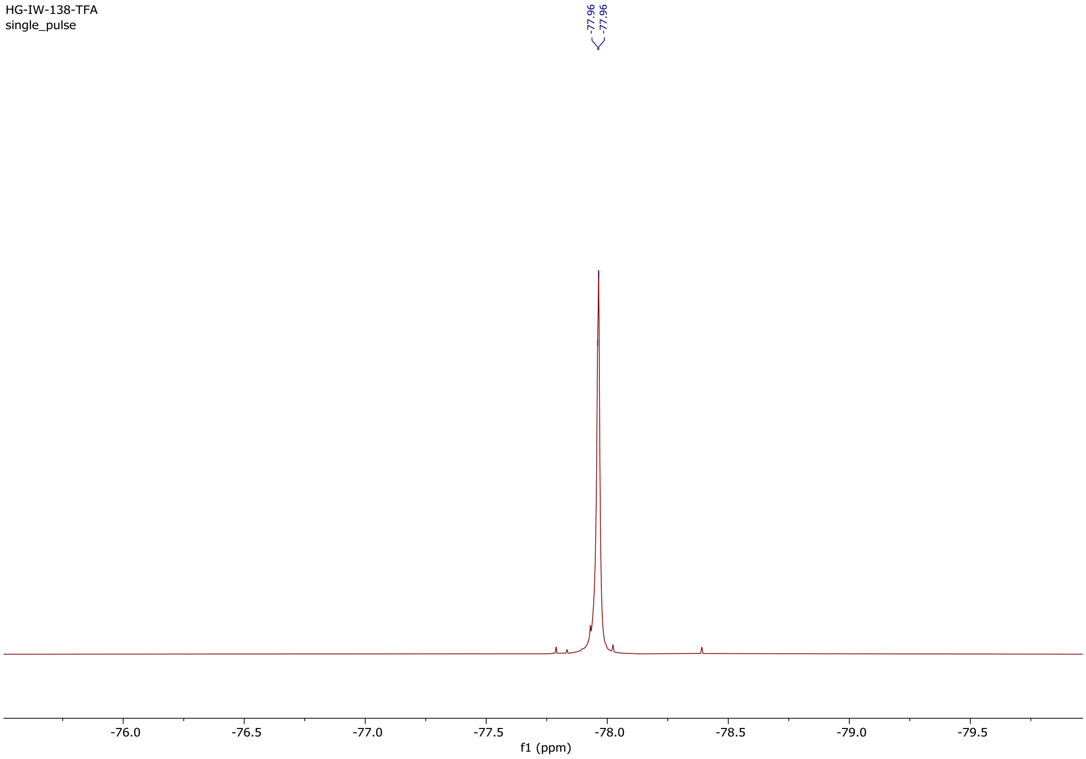


**Figure 25** $^{1}$H, $^{13}$C NMR and $^{19}$F spectra of **2w**

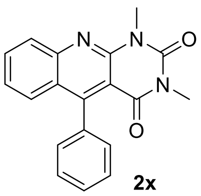

**2x**

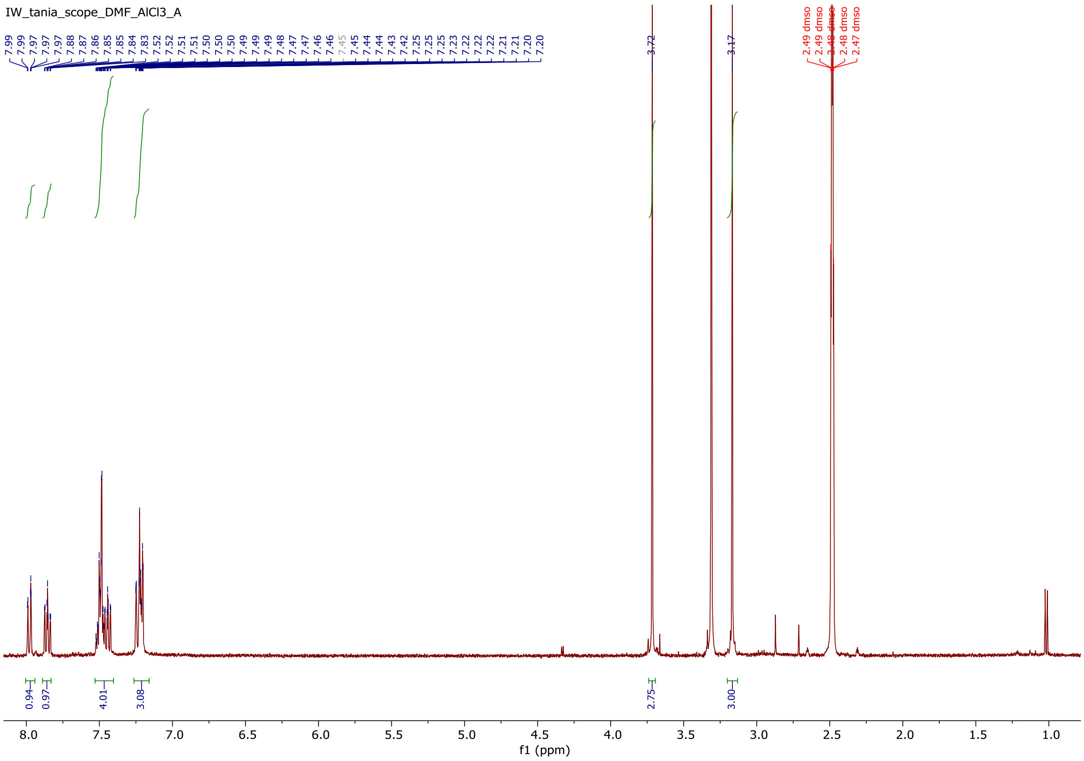

IW_tania_scope_DMF_AlCl3_A
3.72
3.17
2.49 dmso
2.49 dmso
2.48 dmso
2.48 dmso
2.47 dmso
0.94
0.97
4.01
3.08
2.75
3.00
f1 (ppm)


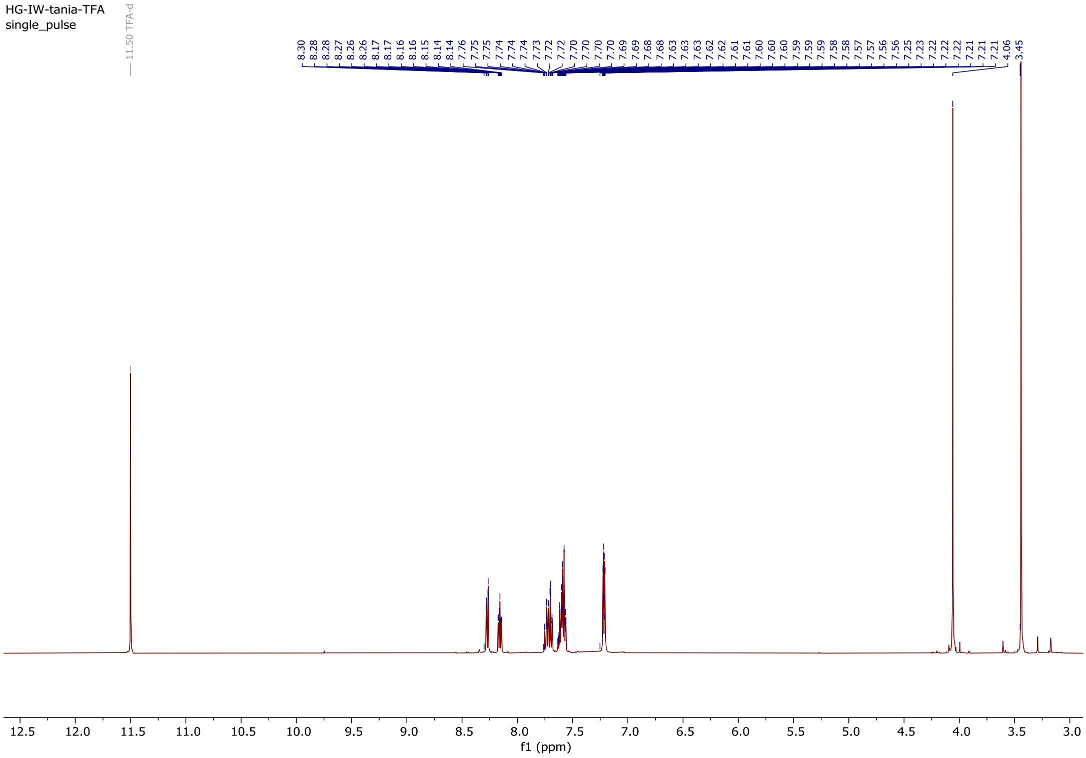

HG-IW-tania-TFA
single_pulse
11.50 TFA-d
4.06
3.45
f1 (ppm)

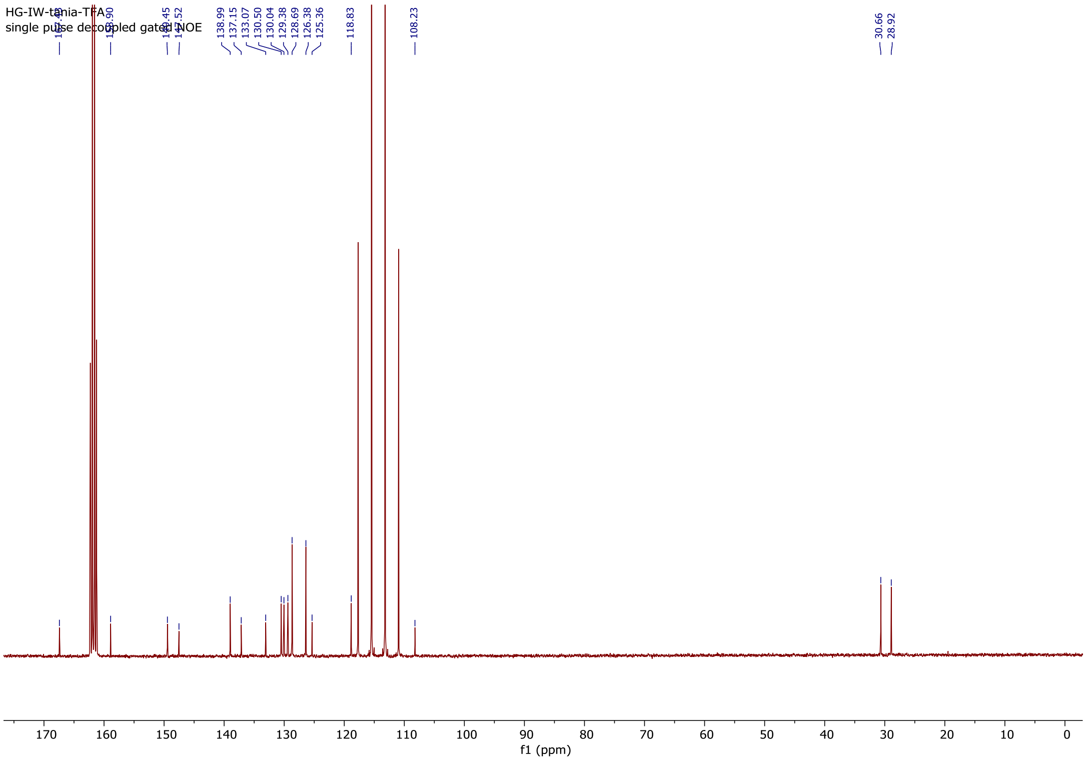


**Figure 26** $^{1}H$ and $^{13}C$ NMR spectra of **2x**

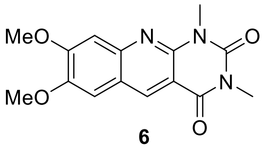


**6**

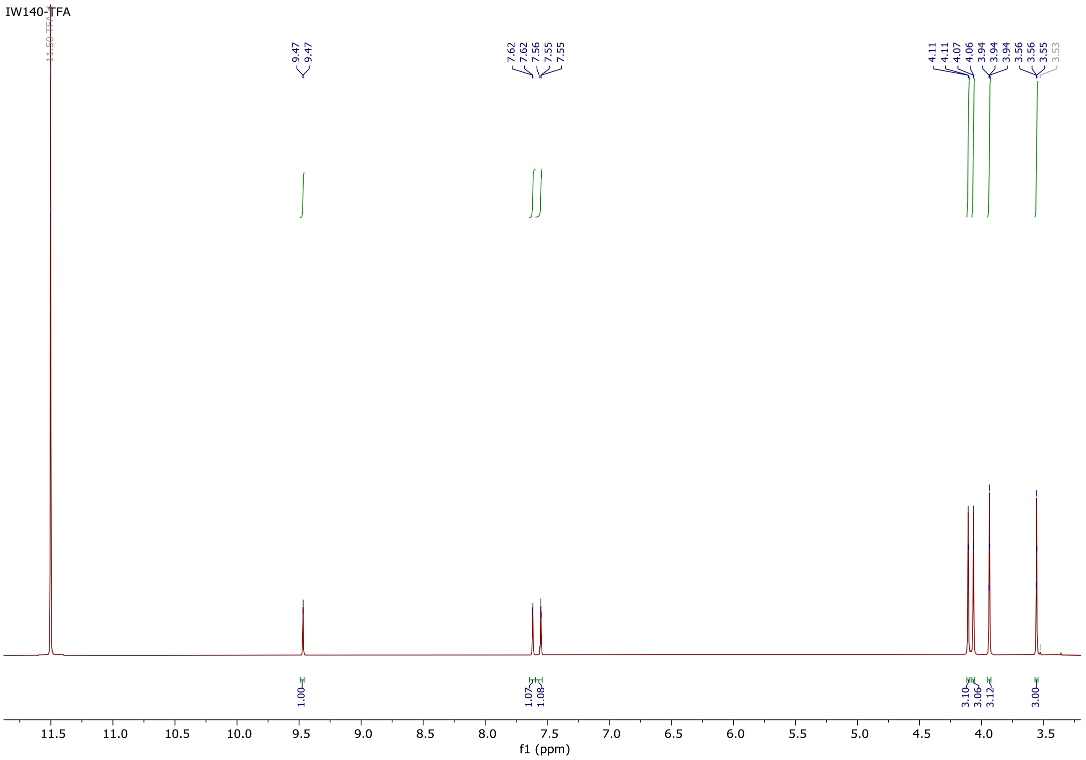


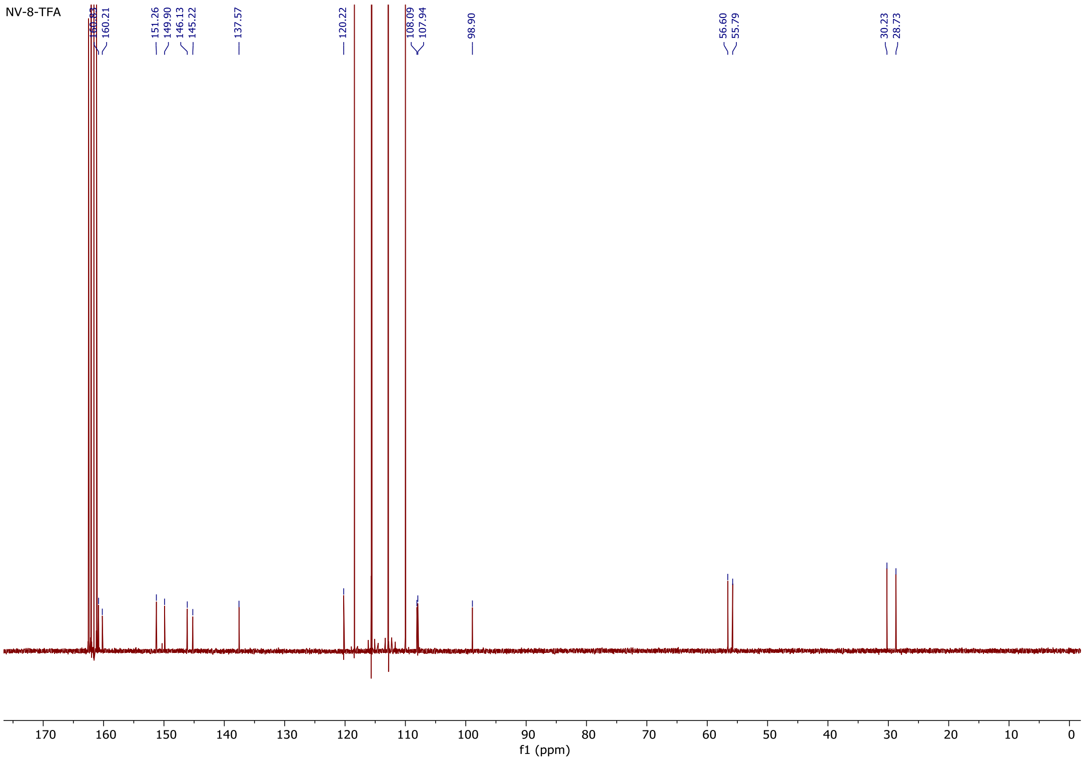


**Figure 27** $^{1}$H and $^{13}$C NMR spectra of **6**

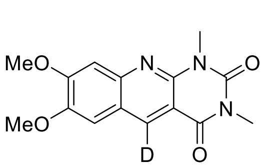
MeO
MeO
N
N
O
N
D
O

**6**

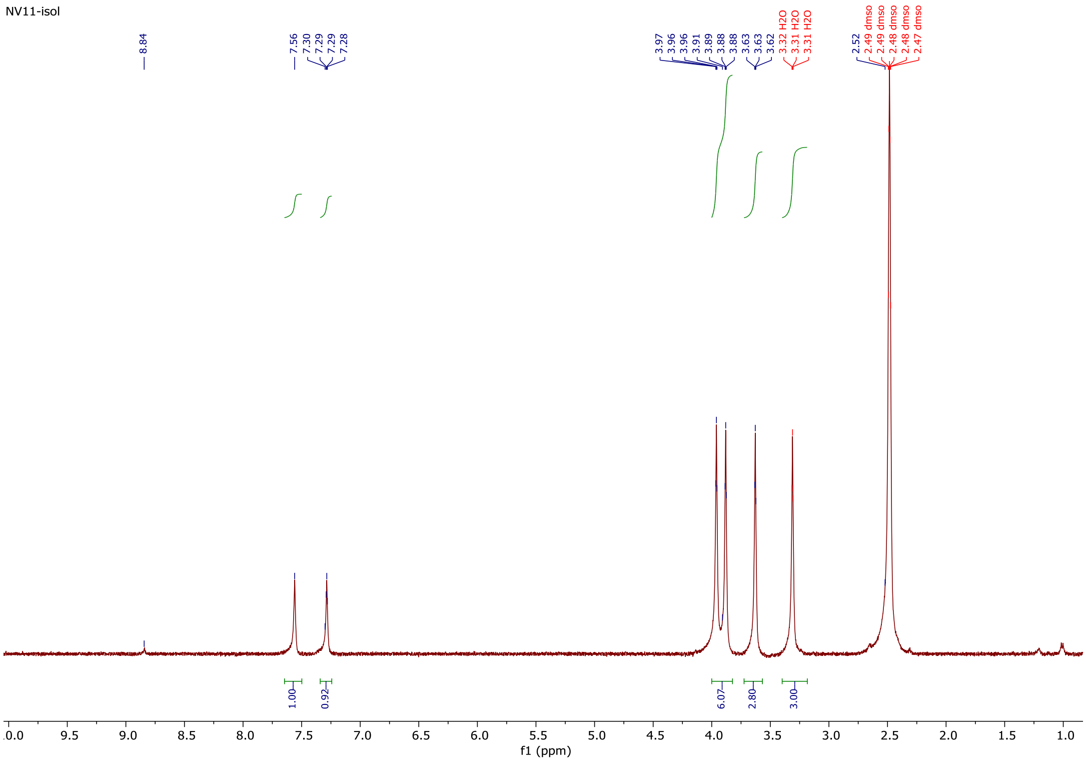
NV11-isol
8.84
7.56
7.30
7.29
7.29
7.28
3.97
3.96
3.96
3.91
3.89
3.88
3.88
3.63
3.63
3.62
3.32 H2O
3.31 H2O
3.31 H2O
2.52
2.49 dmso
2.49 dmso
2.48 dmso
2.48 dmso
2.47 dmso
1.00
0.92
6.07
2.80
3.00
f1 (ppm)

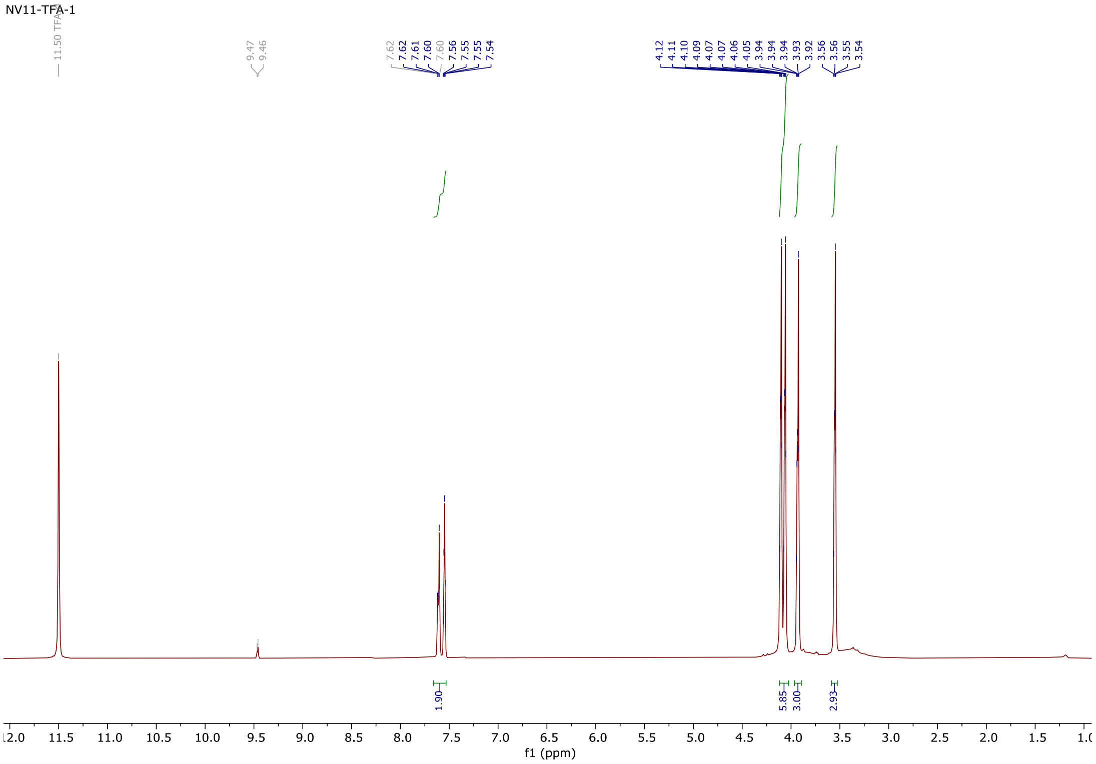
NV11-TFA-1
11.50 TFA
9.47
9.46
7.62
7.62
7.61
7.60
7.60
7.56
7.55
7.55
7.54
4.12
4.11
4.10
4.09
4.07
4.07
4.06
4.05
3.94
3.94
3.93
3.92
3.56
3.55
3.54
1.90
5.85
3.00
2.93
f1 (ppm)

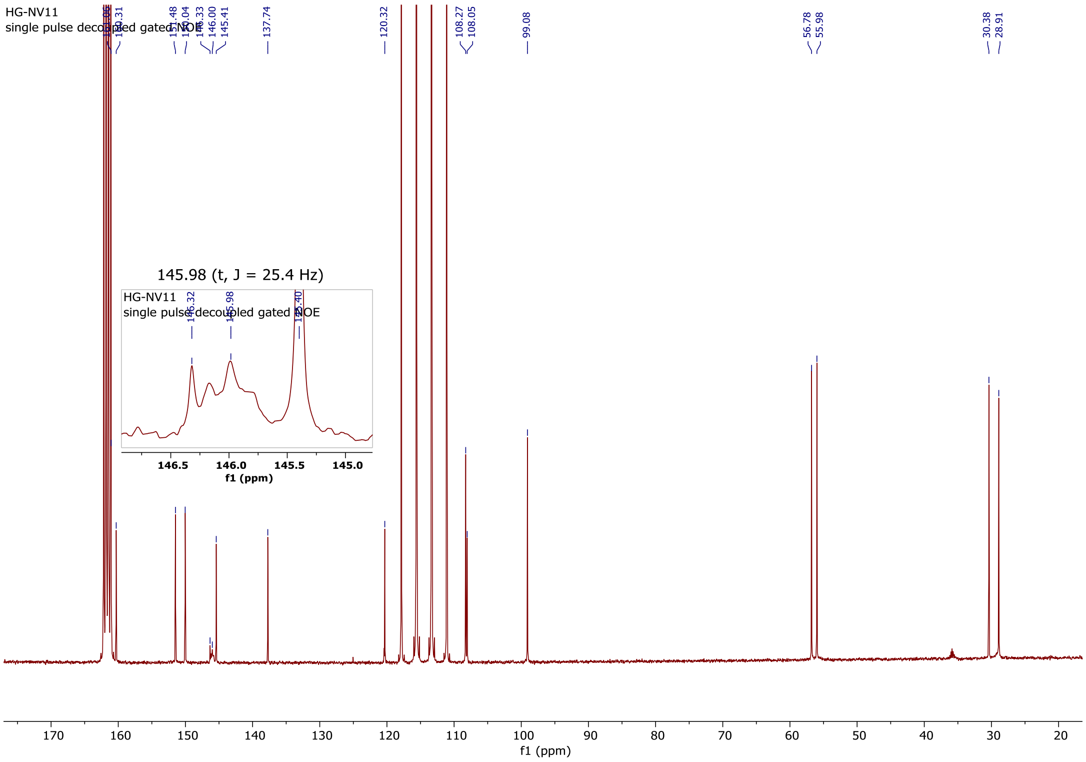


**Figure 28** $^{1}H$ and $^{13}C$ NMR spectra of ***6-d***

## References


1. Pavlovska, T.; Weisheitelová, I.; Pramthaisong, Ch.; Sikorski, mM.; Jahn, U.; Cibulka, R. *Adv. Synth.Catal.* **2023**, *365* (24), 4662-4671. https://doi.org/10.1002/adsc.202300843
2. *Nishigaki,S.; Sato, J.; Shimizu, K.; Furukawa, K.; Senga, K.; Yoneda, F. Chem. Pharm. Bull. 1980, 28 (1), 142-149. https://doi.org/10.1248/cpb.28.142*
3. *Pallabi Saikia, P.; Sharma, G.; Gogoi, S.; Boruah, R. C. RSC Adv., 2015, 5, 23210-23212. https://doi.org/10.1039/C5RA00725A*
4. *Panday, A. K.; Mishra, R.; Jana, A.; Parvin, T.; Choudhury, L. H. J. Org. Chem. 2018, 83 (7), 3624-3632. https://doi.org/10.1021/acs.joc.7b03272*
5. Yadav, P.; Awasthi, A.; Gokulnath, S.; Tiwari, D. K. *J. Org. Chem.* **2021**, *86* (3), 2658-2666. https://doi.org/10.1021/acs.joc.0c02696
6. Shi, X.; Zhang, Q.; Wang, A.; Jang, T.-Sh. *Adv. Synth. Catal.* **2022**, *364*, 2086–2090. https://doi.org/10.1002/adsc.202200284
